# The role of cuproptosis-related gene in the classification and prognosis of melanoma

**DOI:** 10.3389/fimmu.2022.986214

**Published:** 2022-10-19

**Authors:** Jin-Ya Liu, Le-Ping Liu, Ze Li, Yan-Wei Luo, Fang Liang

**Affiliations:** ^1^ Department of Plastic Surgery, The Third Xiangya Hospital of Central South University, Changsha, China; ^2^ Department of Blood Transfusion, The Third Xiangya Hospital of Central South University, Changsha, China; ^3^ Department of Pediatrics, The Third Xiangya Hospital, Central South University, Changsha, China; ^4^ Department of Hematology and Critical Care Medicine, The Third Xiangya Hospital, Central South University, Changsha, China

**Keywords:** melanoma, subtype, machine learning, prognostic model, metastasis model, cuproptosis

## Abstract

**Background:**

Melanoma, as one of the most aggressive and malignant cancers, ranks first in the lethality rate of skin cancers. Cuproptosis has been shown to paly a role in tumorigenesis, However, the role of cuproptosis in melanoma metastasis are not clear. Studying the correlation beteen the molecular subtypes of cuproptosis-related genes (CRGs) and metastasis of melanoma may provide some guidance for the prognosis of melanoma.

**Methods:**

We collected 1085 melanoma samples in The Cancer Genome Atlas(TCGA) and Gene Expression Omnibus(GEO) databases, constructed CRGs molecular subtypes and gene subtypes according to clinical characteristics, and investigated the role of CRGs in melanoma metastasis. We randomly divide the samples into train set and validation set according to the ratio of 1:1. A prognostic model was constructed using data from the train set and then validated on the validation set. We performed tumor microenvironment analysis and drug sensitivity analyses for high and low risk groups based on the outcome of the prognostic model risk score. Finally, we established a metastatic model of melanoma.

**Results:**

According to the expression levels of 12 cuproptosis-related genes, we obtained three subtypes of A_1_, B_1_, and C_1_. Among them, C_1_ subtype had the best survival outcome. Based on the differentially expressed genes shared by A_1_, B_1_, and C_1_ genotypes, we obtained the results of three gene subtypes of A_2_, B_2_, and C_2_. Among them, the B_2_ group had the best survival outcome. Then, we constructed a prognostic model consisting of 6 key variable genes, which could more accurately predict the 1-, 3-, and 5-year overall survival rates of melanoma patients. Besides, 98 drugs were screened out. Finally, we explored the role of cuproptosis-related genes in melanoma metastasis and established a metastasis model using seven key genes.

**Conclusions:**

In conclusion, CRGs play a role in the metastasis and prognosis of melanoma, and also provide new insights into the underlying pathogenesis of melanoma.

## Introduction

Melanoma is a malignant tumor caused by aberrant melanocyte proliferation. It has a high fatality rate and is prone to metastasis. According to the 2020 global cancer statistics, skin melanoma ranks 19th among the most common cancers in the world (1), with the number of new cases rising to 324,635 and the number of deaths rising to 57,043. Melanoma is one of the malignant tumors with an extremely high metastasis rate. Its metastasis is characterized by local metastasis through lymphatics first, and then systemic metastasis through blood. Local surgery is the main treatment for early melanoma, while palliative remission is the main treatment for aggressive metastatic melanoma due to poor treatment effects (2). Second, as the most heterogeneous tumor, melanoma is prone to misdiagnosis and treatment failure (3). Melanoma can be classified into nine types according to epidemiology, clinical and histologic morphology, and genomic characteristics, namely low-cumulative solar damage (CSD) melanoma, high-CSD melanoma, Desmoplastic melanoma, Spitz melanomas, Acral melanoma, Mucosal melanomas, Melanomas arising in congenital nevi, Melanomas arising in blue nevi, Uveal melanoma (4). Characteristics of precursor lesions of different subtypes play a certain role in the prevention and early treatment of melanoma. Ultraviolet radiation is one of the main risk factors for the formation of melanoma, and sun exposure is also an important criterion for classifying melanoma types (5). However, little research has been done on melanoma subtypes. Due to the high mortality rate of melanoma, subtyping studies are also extremely important for the individualized treatment of patients.

Cuproptosis is a novel form of cell death induced by copper ionophores (6, 7). Under normal circumstances, cells maintain a relatively low level of intracellular copper through homeostatic mechanisms to prevent excessive copper accumulation leading to cellular damage. Copper ions in the body combine with enzymes and play a major role in blood coagulation, hormone maturation, and energy metabolism (8–11). Within tumor tissue, unbalanced copper levels can cause irreversible damage to tumor tissue. It induces various forms of tumor cell death including apoptosis and autophagy through mechanisms such as reactive oxygen species accumulation, proteasome inhibition, and anti-angiogenesis (11, 12). Studies have shown that copper chelate, taken orally with food, has antitumor and antimetastatic benefits in animals and humans (13). Recent studies have identified specific roles of copper in oncogenic signaling pathways and antitumor drug resistance (14).

In recent years, machine learning has been applied more and more deeply in the field of life sciences, and more and more studies have shown that machine learning plays an important role in medical big data and can effectively mine new information (15–18). With the development of microarray and sequencing technology, the gene expression data of various diseases is also increasing, and machine learning has emerged in the processing of gene expression data of various cancers (19). Machine learning can predict the occurrence and prognosis of cancer, as well as unearth new biomarkers of cancer (20, 21). This study aims to use machine learning combined with bioinformatics to classify melanoma based on cuproptosis-related genes (CRGs) and to establish melanoma prognosis and metastasis models.

**Figure 1:**
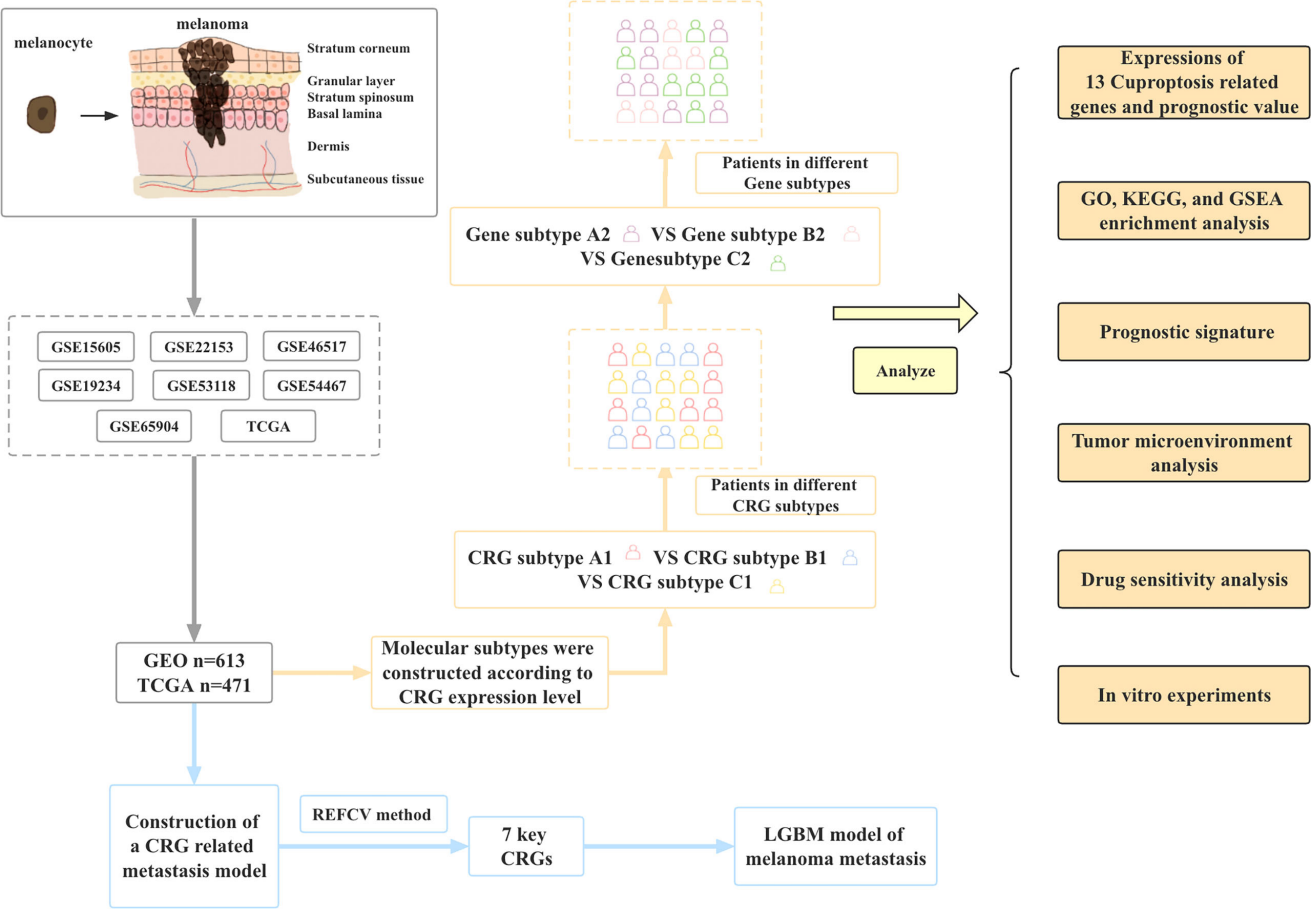
Article framework and workflow.

In this study, we combined the transcriptional information of melanoma samples from seven GEO datasets and TCGA datasets to screen out a total of 12 CRGs. Then the molecular subtypes and gene subtypes of CRGs were constructed according to clinical characteristics and gene expression. Next, we explored the prognostic role of these CGRs between different subtypes, performed functional analysis of differentially expressed genes between different subtypes, and established a prognostic model. In addition, we performed tumor microenvironment analysis and drug sensitivity analysis. Finally, to further understand the role of CRGs in melanoma development, we established metastasis models based on CRGs using 9 different machine learning algorithms. **Figure 1** shows the flow chart of this study.

## Methods

### Patients and datasets

We screened melanoma datasets in two databases, GEO (https://www.ncbi.nlm.nih.gov/geo/.) and TCGA (http://portal.gdc.cancer.gov/). A total of 7 datasets related to prognosis and metastasis were downloaded from the GEO database [datasets containing prognostic information: GSE19234, GSE22153, GSE54467, GSE69504 (394 melanoma samples)]. Datasets containing metastasis information: GSE15605, GSE22153, GSE46517 (219 samples)). Similarly, we screened melanoma samples in the TCGA database and found 472 samples with prognostic information, of which 471 were melanoma samples. We merged datasets containing prognostic information (GSE19234, GSE22153, GSE54467, GSE69504) with the TCGA dataset. Then, the “perl” language was used to convert the probe matrix into a genes matrix based on the annotation information. Next, we converted the TCGA dataset to TPM format, so that the data form of TCGA was more similar to that of GEO. The “merge” package was used to merge the TCGA dataset with the GEO dataset, and the “sva” package in the R language was used to do a batch correction. Finally, we obtained 862 melanoma samples containing prognostic information and 628 samples containing metastasis information, respectively. In subsequent analyses, we used these combined datasets to build melanoma prognostic models and metastasis models.

### Expression of CRGs in melanoma

In the TCGA cohort, “maftools” was used to map the mutation frequencies of CRGs, shown as waterfall plots. Likewise, we analyzed the copy number of CRG in melanoma. The “RCircos” package was used to draw copy number circle diagrams. Next, we constructed a prognostic model based on 12 CRGs (7) in the combined TCGA and GEO cohort. First, we extracted the expression levels of CRGs in datasets with prognostic information and then merged clinical data. The “survival” package was used for survival analysis, cox analysis was used for univariate analysis, and KM analysis was used for survival status analysis.

### Construction of molecular subtypes of CRGs

We obtained 13 CRGs (**Supplementary Table 1**) from previous studies, and after deleting unexpressed CRGs in some samples, we finally selected 12 CRGs for model construction. Consensus Clustering is an unsupervised clustering method and a common research method for cancer subtype classification. It can differentiate samples into different subtypes based on different omics data sets, so as to discover new disease subtypes or perform a comparative analysis of different subtypes. The “ConsensusClusterPlus” R package was used to perform consensus clustering to distinguish different molecular subtypes based on the mRNA expression levels of 12 CRGs. Next, to further analyze the differences between subtypes. We adopted the t-distributed stochastic neighbor embedding (t-SNE) method to explore the distribution of different subtypes, and the “R t sne” R package was used to estimate the effect of classification. Furthermore, we analyzed the extent of immune cell infiltration between different subtypes. The “heatmap” R package was used to analyze the expression levels of CRGs, tumor grade, gender, and age among different subtypes. Finally, the “GSVA” R package was used to analyze the enriched pathways between different subtypes and displayed as heatmaps.

### Survival analysis of gene subtype and differential expression analysis of CRGs

To further understand the correlation between molecular subtypes and differentially expressed genes, we performed gene subtypes. The “limma” R package was used to analyze the differentially expressed genes between different subtypes (logfc > 0.585, p-value < 0.05). After obtaining the differentially expressed genes between each subtype, we took the intersection genes for subsequent analysis. “clusterPrfiler” was used to perform GO enrichment analysis (p-value < 0.05). Similarly, Metascape website (http://metascape.org) (version 2022-04-22) was used to perform enrichment analysis of 71 intergenes.Terms with a P value1.5 are collected and grouped into clusters depended on their membership similarities. The “limma” and “survival” packages were used to analyze the differentially expressed genes associated with prognosis. The Univariate cox regression analyses were used to find intersecting genes associated with prognosis (p-value<0.05). Next, we used the Consensus Clustering method to type the samples according to the expression levels of the intersecting genes. After finding the subtype with the highest internal correlation, survival analysis and clinical trait analysis were performed on different subtypes. We show the above analysis results with KM curve and heat map respectively. Finally, the “limma” package was used to analyze the expression levels of CRGs in different gene types and displayed as boxplots.

### Construction of the prognostic model

We divide the samples into training and validation sets in a 1:1 ratio. In the training set, differentially expressed genes associated with prognosis were used to perform Least Absolute Shrinkage and Selection Operator (LASSO) Cox regression analysis through the “glmnet” R package. The risk score was equal to the LASSO regression coefficient for each mRNA multiplied by the sum of the normalized expression levels for each mRNA. Next, we analyzed the AUC of the training set, the validation set, and all samples. Then, based on the samples with survival information, nomogram plots were constructed using the “rms” R package to predict the 1-, 3-, and 5-year survival probabilities of patients. A calibration plot was constructed to assess the agreement of the probabilities predicted by the nomogram with the actual values.

### Tumor microenvironment and drug sensitivity analysis

The “CIBERSORT” package was used to perform immune cell infiltration analysis. We analyzed the correlation between 6 key variable genes (AIM2, EDNRB, SLC39A6, TMEM117, PTPRC, and KIF14) and immune cells. At the same time, we also analyzed the correlation between the two prognostic risk groups and the tumor microenvironment. The “estimate” package was used to score the tumor microenvironment in the high-risk and low-risk groups and displayed in a violin plot. Then, we performed a drug sensitivity analysis based on the risk score results. We combined the sample’s risk score and drug sensitivity. Then, the high-risk and low-risk groups were analyzed for their sensitivity to the drug, and results with significant differences (p-value > 0.001) were represented by boxplots.

### Construction of metastasis model

We integrated all GEO datasets (GSE15605, GSE21153, GSE46517) with melanoma metastasis information. 70% of the samples were set as the training set, and the remaining 30% of the samples were set as the validation set. We used the REFCV method to screen out key metastatic variables by python 3.7. The main idea of recursive feature elimination (REF) is to build the model iteratively and then select the best (or worst) features (selected according to the coefficients). Set the selected features aside and repeat the process on the remaining features until all features are traversed. The order that is eliminated in this process is the ordering of features. REFCV is REF + CV (cross-validation). Its operating mechanism is first to use REF to obtain the ranking of each feature, and then based on the ranking, select [min_features_to_select, len(feature)] feature subsets for model training in turn and cross-validation, and finally select the feature subset with the highest average score. (python 3.7 sklearn 0.22.1 package).

We then use these key variables to build models using 9 different machine learning algorithms (XGBoost’, ‘Logistic’, ‘LightGBM’, ‘RandomForest’, ‘AdaBoostClassifier’, ‘GaussianNB’, ‘ComplementNB’, ‘SVC’ ‘, ‘KNeighbors). Using the cross-validation method, the random seed is set to 1 and the fold is 15. The performance of each model was compared using multi-model forest plots, AUC, accuracy, and F1 values to screen out the best performing models. All Statistical analyses in the process of construction of the metastasis model were performed using python version 3.7 and the Extreme Smart Analysis platform (https://www.xsmartanalysis.com/) (22).

### Interpretability of the metastasis model

After filtering out the best performing models, use the “SHAP” package (version 0.39.0, python 3.7) to explain the importance and contribution of key variables to the model. At the same time, use the force diagram to illustrate 2 samples to show how different variables contribute in different samples (“SHAP” package version 0.39.0, python 3.7). All Statistical analyses in this part were performed using python version 3.7 and Extreme Smart Analysis platform (https://www.xsmartanalysis.com/).

### Statistical analysis

The “survival” package was used for survival analysis, cox analysis was used for univariate analysis, and KM analysis was used for survival status analysis. Principal Component Analysis (PCA) was used to demonstrate the differences between CRG subtypes.The “ConsensusClusterPlus” package was used for the subtyping of CRG subtypes and gene subtypes. Lasso regression was used to screen for genes associated with prognosis, and prognostic models were developed using multivariate regression analysis. Wilcoxon rank sum test was used to compare TME scores between the high-risk and low-risk groups. The ROC curve was used to assess the predictive power of the prognostic model. There are several R packages, including “RCircos”, “heatmap”, and “ggplot” packages for generating graphs. P < 0.05 is considered statistically significant.The python software (version 3.7) used in the establishment of the melanoma metastasis model was used for statistical analysis. The REFCV method of the sklearn 0.22.1 package was used to screen key variables in the melanoma metastasis model. In the modeling process of various machine learning algorithms, the xgboost 1.2.1 package was used to perform the XGBoost algorithm, the lightgbm 3.2.1 package was used to perform the LightGBM algorithm, and the sklearn 0.22.1 package was used Used to run other machine learning algorithms. The shap 0.39.0 package was used to demonstrate model interpretability (SHAP graph, feature importance ranking graph, force graph).

### Cell lines and constructs for transfection

Human malignant melanoma cell line A375 were cultured in Dulbecco’s modified Eagle’s medium (DMEM, Gibco), supplemented with 10% (v/v) heat-inactivated fetal bovine serum (FBS, Gibco) at 37°C in a humidified incubator containing 5% CO2. FDX1 siRNAs (1#: 5’-CAUUAACAACCAAAGG AAA-3’, 2#: 5’-CAUCUUUGAAGAUCACAUA-3’) and control siRNA (5’-UUCUCCGAACGU GUCACGU-3’) were obtained from Sangon (Shanghai, China). Transfection of siRNAs was performed with Lipofectamine RNAiMAX Transfection Reagent (Thermo Fisher) as recommended.

### Western blot analysis

The protein was extracted using RIPA buffer (Beyotime) and the protein concentration was determined using the BCA Protein Assay Kit (Pierced, Grand Island, NY). Protein samples were separated by 12% SDS-PAGE and transferred onto polyvinylidene difluoride membranes ((PVDF, Millipore). To assess the protein expression, the blots were incubated with the primary rabbit antibodies against FDX1 (Abcam) and anti-rabbit secondary antibodies (Cell Signaling Technology) at a dilution of 1:2000 for 1 h at room temperature. β-Actin(Cell Signaling Technology) served as an endogenous control for equal loading.

### CCK-8 experiment

The CCK-8 reagent was purchased from GLPBIO (GK10001). Briefly, A375 cells transient transfecting FDX1 siRNA (siFDX1) or the control siRNA (siNC) were seeded at 2x104 cells per well in 96-well plates in quintuplicate, the number of viable cells in each well was measured at 0, 12, 24, and 36 hours according to the manufacturer’s instructions.

### Wound healing

For wound healing assay, when the cells were grown to 90% confluence after transfection, a straight scratch in the cell monolayer was created by a 10μL pipette tip. A375 cells were incubated with 2% FBS. Images of the scratched area (wound) were taken at the time point of 0h, 24 h, 36 h, and 48 h under a microscope. Wound closure= (original wound area - existing wound area)/original wound area. The area of wound healing was calculated by Fiji (version Fiji for Mac OS X).

### Vitro experiment statistical analysis

Statistical analysis was performed using software of Graph Pad Prism 5 (GraphPad, La Jolla, CA). Student’s t-tests were used to evaluate significant differences between any two groups of data. All data are represented as means ± SEM. Differences were considered significant if p < 0.05.

## Results

### Article framework and workflow

Flow chart of data collection and data analysis for the article (**Figure 1**).

#### Mutation frequency and prognostic value of CRGs in melanoma

Among the 467 samples in the TCGA dataset, 56 patients had CRGs mutations (S1 A). Meanwhile, CRGs chromosome positions are shown as copy number variant plots (S1 B). Besides, the frequency of CRGs copy number variation in the samples is shown graphically (S1 C), with red representing an increase in copy number and green representing a decrease in mutation. The graphs show a significantly reduced number of mutations in DBT, FDX1, and DLA. Next, we analyzed the association of CRGs with prognosis after combining the TCGA and GEO datasets. 9 of the 13 CRGs were associated with prognosis (S2 A-I). Moreover, Kaplan–Meier analysis results revealed that a higher expression of LIPT1, FDX1, LIAS, and DBT was associated with a better OS (P < 0.05), and a lower expression of ATP7B, SLC31A1, PDHA1, DLD, and DLST was associated with a better OS (P < 0.05).

### Construction of CRGs molecular subtypes of melanoma

**Figure 2:**
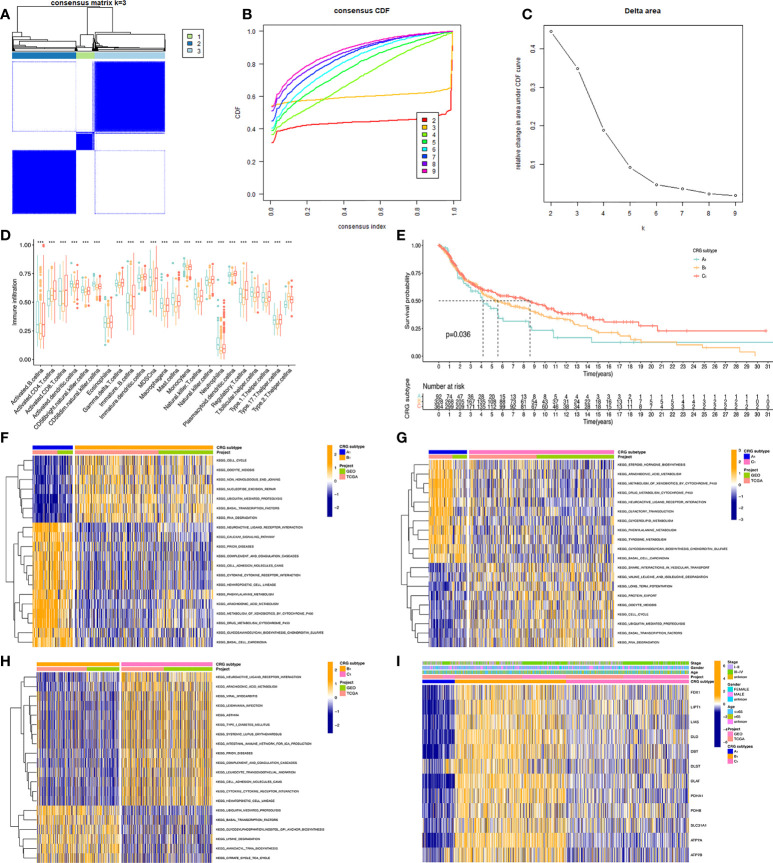
Classification of melanoma based on CRGs. **(A)** Molecular subtypes based on CRGs obtained under unsupervised consensus clustering. **(B)** The empirical cumulative distribution function (CDF) plot depicts the consistent distribution of different K values. **(C)** Relative increase in cluster stability by delta area fraction. **(D)** Comparison of the degree of immune cell infiltration of the three molecular subtypes*, P<0.05; **, P<0.01; ***, P<0.001. **(E)** Kaplan Meier analysis results of three molecular subtypes based on 12 CRGs. **(F, G, H)** pictures show the enriched pathways of differentially expressed genes obtained by comparing A_1_, B_1_, and C_1_ molecular subtypes with each other using the GSVA method. **(I)** Heatmap of clinical information and gene expression profiles of the three molecular subtypes based on 12 CRGs.

To obtain the melanoma subtypes of CRGs, we performed a consensus clustering analysis on the expression level of CRGs on the combined GEO and TCGA datasets. In the cluster analysis of 862 samples, K = 3 was the optimal number of clusters. When K=3, the difference between groups was the smallest, and the difference outside the group was the largest. Therefore, we accurately divided melanoma patients into 3 subtypes, namely A_1_, B_1_, and C1 (**Figure 2A**). When dividing melanoma patients into 3 subtypes, the relative change in the area under the CDF curve indicated that the stable distribution of melanoma patients was close (**Figure 2B, C**). In the Kaplan Meier analysis of A_1_, B_1_, and C_1_ subtypes, the survival outcome of the C_1_ subtype was the best, followed by the B_1_ subtype, and the worst survival outcome of the A_1_ subtype (**Figure 2D**).

### Comparative analysis between three CRGs molecular subtypes

We present the expression level of CRGs and clinical traits, such as Stage, Gender, and Age of the A_1_, B_1_, and C_1_ subtypes in a heat map. CRGs were expressed at the highest level in the B_1_ subtype, followed by the C_1_ subtype, and lowest in the A_1_ subtype. Then, GSVA enrichment pathway analysis was performed on three different subtypes (**Figures 2F–H**). Comparing the A_1_ subtype and the B_1_ subtype, it was found that the B_1_ subtype was significantly more enriched than the A_1_ subtype in cell cycle, non-homologous end linkage, and ubiquitination-mediated hydrolytic protein action. A1 subtype showed significantly higher levels of enrichment in pathways such as neuroactive ligand receptor interactions, cytochrome p450 effects on foreign biometabolism, and drug metabolism of cytochrome p450 than B_1_. Comparing the A_1_ and C_1_ subtypes, the A_1_ subtype showed significantly higher levels of enrichment in the drug metabolism cytochrome p450, glycerolipid, and tyramine metabolism pathways than the C_1_ subtype. The C_1_ subtype was slightly more enriched than the A_1_ subtype in pathways such as trap interactions in vesicle transport, ubiquitin-mediated protein hydrolysis, and protein efflux. Comparing the B_1_ subtype and C_1_ subtype, the enrichment level of the C_1_ subtype is higher than that of the B_1_ subtype in pathways such as neuroactive ligand receptor interaction, complement system, and leukocyte endothelial migration. The B_1_ subtype was significantly more enriched in ubiquitin-mediated protein hydrolysis, aminyl biosynthesis, and citric acid cycle TCA cycle pathways than the C_1_ subtype.

Further, we analyzed the level of immune cell infiltration between three CRGs subtypes. Among the 23 immune cells, most of them differed in their degree of infiltration in the A_1_, B_1_, and C_1_ subtypes. Among them, Myeloid-derived suppressor cells (MDSC), Immature B cells, and active B cells had the highest difference in the degree of infiltration, and only Eosinophilna cells had no difference in the degree of infiltration. Overall, the highest level of immune cell infiltration was found in the C_1_ subtype and the lowest in the B_1_ subtype.

### Enrichment analysis of genes with intersections of CRGs subtypes

**Figure 3:**
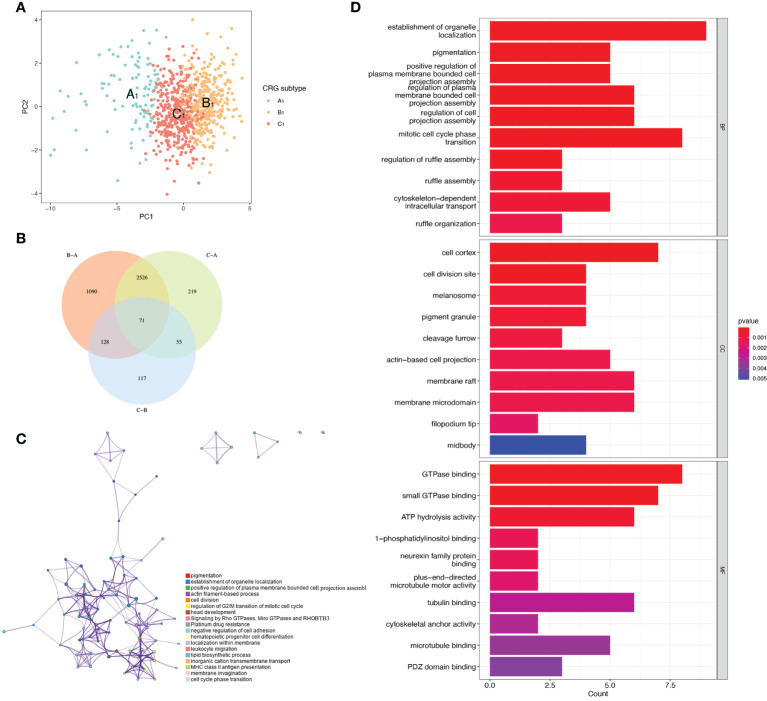
Differentially expressed genes of three CRGs molecular subtypes. **(A)** VENN plot showing 71 intersecting differentially expressed genes across three molecular subtypes. **(B)** t-distributed Stochastic Neighbor Embedding (tSNE) analysis of three CRGs molecular subtypes. **(C)** Metascape enrichment analysis of DEGs with intersections of the three molecular subtypes. **(D)** GO enrichment analysis of DEGs with intersections of the three molecular subtypes.

t-distributed stochastic neighbor embedding(tSNE) analysis showed that the A_1_, B_1_, and C_1_ subtypes are distinguishable from each other. This indicates that our subtype analysis based on CRGs has better typing ability (**Figure 3A**). Next, we analyzed the differentially expressed genes between A_1_, B_1_, and C_1_ subtypes. There were 1090 differentially expressed genes between A_1_ and B1 subtypes, 117 differentially expressed genes between A_1_ and C_1_ subtypes, and there are 219 Differentially expressed genes between the B_1_ and C_1_ subtypes. We intersected the differentially expressed genes of the three subtypes and obtained 71 differentially expressed genes that were co-expressed in the three subtypes (**Figure 3B**). Enrichment analysis in Metascape showed that differentially expressed genes were mainly associated with Signaling by Rho GTPases, Miro GTPases and RHOBTB3, MHC class II antigen presentation, and Platinum drug resistance (**Figure 3C**). GO (Gene ontology) enrichment analysis indicates the results of intersecting genes in BP (Biological Process), CC (Cellular Component), MF (Molecular Function) respectively (**Figure 3D**). BP is primarily associated with the establishment of organelle localization, mitotic cell cycle phase transitions, and cytoskeletal-intracellular transport dependence. CC is associated with cell cortex, cell division sites, and membrane microstructure domains. MF is mainly associated with the guanosine triphosphatase binding region, ATP hydrolysis activity, and microtubule binding proteins.

### Construction of gene subtypes

To further understand the correlation between CRGs subtypes and differentially expressed genes, we constructed gene subtypes. We performed univariate regression analysis on 71 differentially expressed genes co-expressed in the three CRGs subtypes, and obtained 16 differentially expressed genes associated with survival. In the cluster analysis, when K=3, we can see that the difference between groups is small, and the difference outside the group is large (S3 A). The comprehensive analysis of the consistent cumulative distribution function (S3 B) and Delt area(S3 C) also shows that K=3 is more suitable. Kaplan Meier analysis was performed on the three gene subtypes, with B_2_ having the best survival outcome, A_2_ having the second worst survival outcome, and C_2_ having the worst survival outcome (S3 D) (P-value<0.001). Then, we illustrate the clinical traits (stage, gender, age)of both gene subtypes and CRGs molecular subtypes in a heat map (S3 E). Besides, we explored the differences in the expression levels of CRGs among the A_2_, B_2_, and C_2_ subtypes. We found that the expression of CRGs was different in A_2_, B_2_, and C_2_ subtypes (p<0.001) (S3 F).

### Construction of the prognostic model

**Figure 4:**
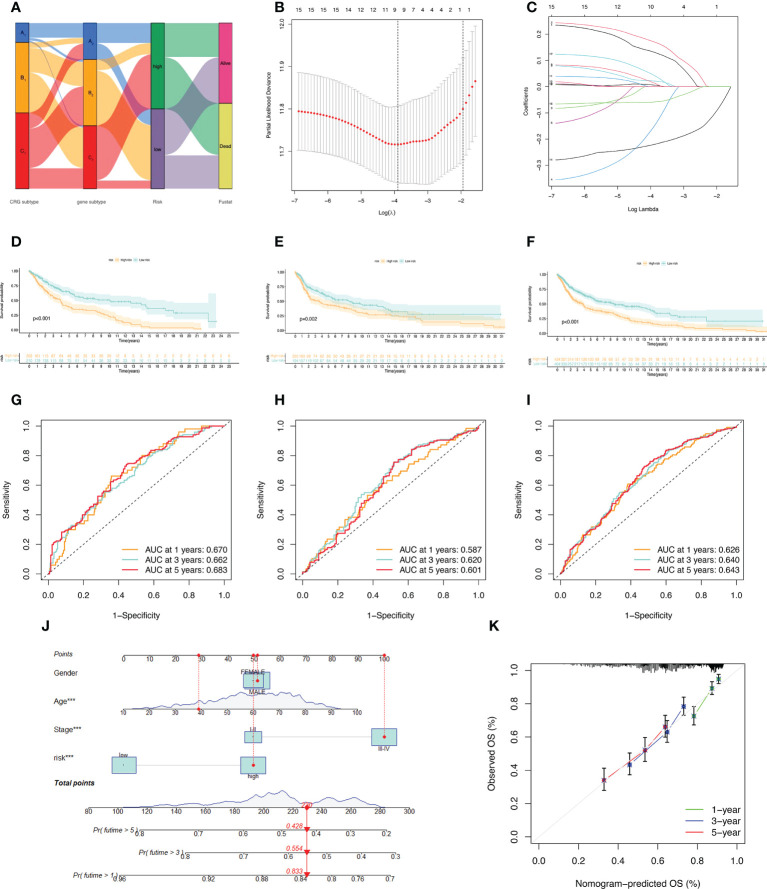
Construction of the prognostic model. **(A)** Sankey diagram to describe the process of constructing a prognostic model based on CRGs-subtypes and gene subtypes. **(B, C)** Prognostic genes were screened using LASSO regression. **(D, G)** Kaplan Meier analysis of OS in melanoma patients in the training set; ROC curves for 6 key variable genes. **(E, H)** OS of melanoma patients in Kaplan Meier analysis validation set; ROC curves of 6 key variable genes. **(F, I)** Kaplan Meier analysis of OS in all melanoma patients; ROC curves of 6 key variable genes. **(J)** Nomograms predicting 1-, 3-, and 5-year OS probabilities in melanoma patients. **(K)** Calibration plots of the nomograms.

A Sankey diagram was used to show our flow chart for two types of melanoma (**Figure 4A**). AIM2, EDNRB, SLC39A6, TMEM117, PTPRC, and KIF14 were screened out by the LASSO regression algorithm to construct a prognostic model (**Figures 4B, C**). In the training set, there was a significant difference in prognostic value between the high-risk and low-risk groups (**Figure 4D**). Survival time was significantly lower in the high-risk group than in the low-risk group. The areas under the time-dependent ROC of the train set are 0.670, 0.662 and 0.683 for 1-, 3-, and 5-year survival. (**Figure 4G**). Next, the prognostic model was applied to the validation set and to the total sample. In the validation set and in the total sample, the prognostic value of the high-risk group was significantly lower than that of the low-risk group (**Figures 4E, F**). The areas under the time-dependent ROC of the validation set are 0.587, 0.620, and 0.601 for 1-, 3-, and 5-year survival (**Figure 4H**). In the total sample, The areas under the time-dependent ROC are 0.626, 0.640 and 0.643 (**Figure 4I**). The ROC of each group shows that our model has better prediction accuracy. Finally, we used nomograms to predict patient survival (**Figure 4J**). Calibration curves showed that our model had high accuracy in predicting patient survival at 1, 3, and 5 years (**Figure 4K**).

### Risk curve and tumor microenvironment

**Figure 5:**
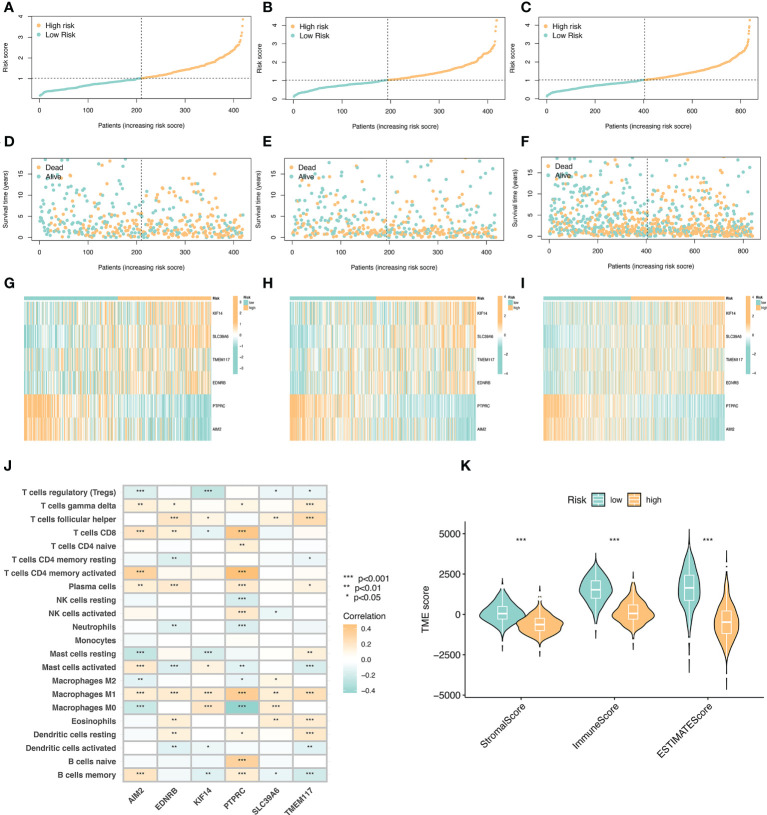
Risk curve and immune microenvironment analysis between high and low immune groups. **(A)** Risk curve in the training set. Take the median of the risk scores and use the median to divide the samples into high-risk and low-risk groups. **(B)** Risk curve in the validation set. **(C)** Risk curves of all samples. **(D)** Survival state diagram of the training set, red for dead and blue for survival. **(E)** The living state diagram of the validation set. **(F)** Survival state diagram of all samples. **(G–I)** Heat map showing the expression of 6 key variable genes in training set, validation set and all samples. **(J)** Correlation of 6 key variable genes with immune cells, red represents positive correlation and blue represents negative correlation. **(K)** Correlation of stroma score, immune score, and ESTIMATE with immune microenvironment.

We arranged the training set, validation set, and all samples according to the prognostic risk model from low to high risk scores, and obtained the risk curve (**Figures 5A–C**).Similarly, we obtained the survival status map between risk scores and death samples (**Figures 5D–F**), and finally, we used heatmaps to show the expression of the model’s key variable genes (AIM2, EDNRB, KIF14, PTPRC, SLC39A6, and TMEM117) in the training set, validation set and all sample (**Figures 5G–I**). Next, we performed tumor microenvironment analysis on 6 key variable genes (**Figure 5J**).

The key variable genes were mainly associated with the degree of infiltration of M1 macrophage, M0 macrophage, and memory B cells. KIF14, SLC39A6, TMEM117, and EDNRB, as high-risk genes, were negatively correlated with the degree of infiltration of memory B cells and regulatory T cells, and positively correlated with the degree of infiltration of M1 macrophage, T follicular helper. AIM2 and PTPRC, as low-risk genes, showed a significant positive correlation with the degree of infiltration of memory B cells, activated memory CD4(+) T cells, and CD8(+) T cells, and a significant negative correlation with the degree of infiltration of M0 macrophage. The stromalscore, immunescore, and ESTIMATEscore scores in the high-risk group were significantly lower than those in the low-risk group (**Figure 5K**).

### Comparison of drug sensitivity, subtypes and expression levels of CRGs between high and low risk groups

**Figure 6:**
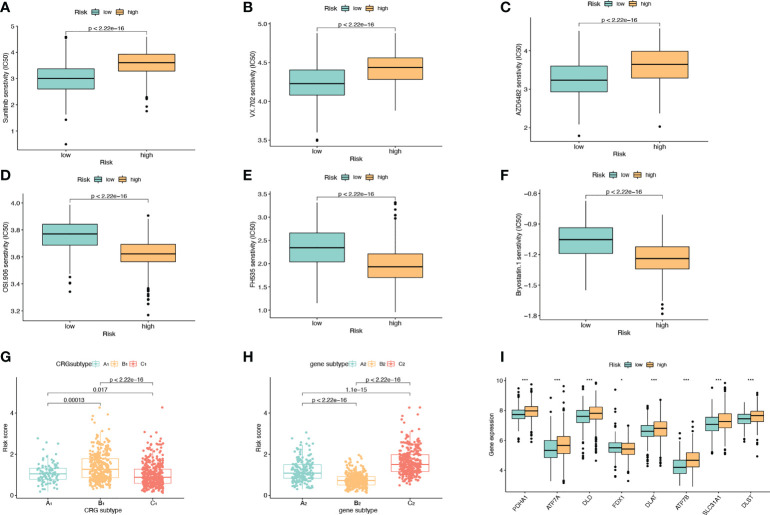
Drug Sensitivity Analysis. **(A–C)** The sensitivity of the low-risk group to Sunitinib, VX.702, AZD6482 was higher than that of the high-risk group. The abscissa is the low-risk group and the high-risk group, and the ordinate is the value of the drug IC50. **(D–F)** The high-risk group had higher sensitivity to OSI.906, FH535, and Bryostatin.1 than the low-risk group. **(G)** Risk scores for A_1_, B_1_, and C_1_ subtypes in CRGs molecular subtypes. **(H)** Risk scores for A_2_, B_2_, C_2_ subtypes in genotyping. **(I)** Expression levels of CRGs in high and low risk groups.

We divided the high-risk group and the low-risk group according to the model. Screening of sensitive drugs was carried out according to the difference in IC50 concentration between the two groups. A total of 98 drugs (S2) were screened, and we selected 3 high-sensitivity drugs in the low-risk group (**Figures 6A–C**) and 3 high-sensitivity drugs in the high-risk group (**Figures 6D–F**). Among the CRG subtypes, B_1_ has the lowest risk score and subtype C_1_ has the highest risk score (**Figure 6G**). Among the genesubtypes, C_2_ had the highest risk score and B_2_ had the lowest risk score (**Figure 6H**). Among the CRGs genes with differential expression in the high-risk and low-risk groups, only FDX1 expression was decreased in the high-risk group (**Figure 6I**).

### Construction of metastasis model

**Figure 7:**
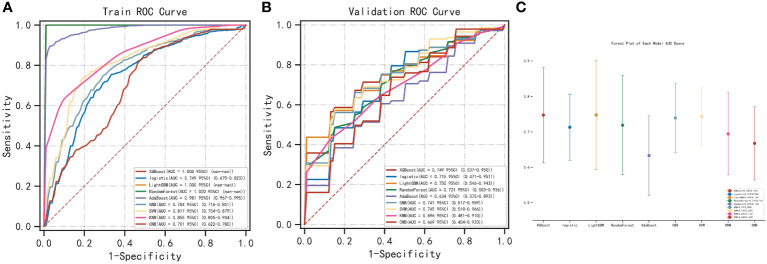
Construction of metastasis model. **(A)** REFCV method to filter out key metastatic variables in train set. **(B)** REFCV method to filter out key metastatic variables in validation set. **(C)** Multi-model forest graph.

We used the REFCV method to filter out key metastatic variables: ‘FDX1’, ‘LIPT1’, ‘LIAS’, ‘DLD’, ‘DBT’, ‘DLAT’, ‘PDHB’. From **Figures 7A, B** we can see that LightGBM has the highest AUC in both training and validation sets, 1 and 0.750, respectively. The values of LightGBM and XGBoost in the multi-model forest graph in **Figure 7C** are also the highest at 0.748. **Tables 1** and **2** show that the AUC, cutoff, accuracy, sensitivity, specificity, positive predictive value, negative predictive value, F1 score, Kappa value of LightGBM are 1.000, 0.637, 0.995, 1.000, 1.000, 1.000, 0.986, 1.000, 0.989. In conclusion, LightGBM is the best performing model, and we choose this model to establish a melanoma metastasis model.

**Table 1:** Multi-model comparison, training set results.

Model	AUC(SD)	cutoff(SD)	accuracy(SD)	sensitivity(SD)	specificity(SD)	positive predictive value (SD)	negative predictive value (SD)	F1 score (SD)	Kappa(SD)
XGBoost	1.000(0.000)	0.863(0.025)	0.995(0.000)	1.000(0.000)	1.000(0.000)	1.000(0.000)	0.986(0.000)	1.000(0.000)	0.989(0.000)
logistic	0.749(0.009)	0.656(0.018)	0.712(0.018)	0.709(0.045)	0.730(0.042)	0.828(0.015)	0.572(0.024)	0.763(0.023)	0.406(0.026)
LightGBM	1.000(0.000)	0.637(0.019)	0.995(0.000)	1.000(0.000)	1.000(0.000)	1.000(0.000)	0.986(0.000)	1.000(0.000)	0.989(0.000)
RandomForest	1.000(0.000)	0.623(0.048)	0.988(0.006)	0.999(0.003)	0.999(0.004)	0.999(0.002)	0.969(0.017)	0.999(0.002)	0.974(0.014)
AdaBoost	0.980(0.004)	0.504(0.001)	0.918(0.014)	0.894(0.025)	0.976(0.017)	0.986(0.010)	0.825(0.031)	0.938(0.012)	0.828(0.028)
GNB	0.783(0.008)	0.629(0.032)	0.744(0.014)	0.772(0.033)	0.706(0.029)	0.828(0.010)	0.620(0.025)	0.798(0.015)	0.456(0.022)
CNB	0.700(0.008)	0.495(0.001)	0.728(0.013)	0.849(0.023)	0.519(0.026)	0.763(0.008)	0.640(0.030)	0.803(0.011)	0.376(0.027)
SVM	0.815(0.006)	0.686(0.014)	0.764(0.010)	0.744(0.024)	0.814(0.025)	0.880(0.011)	0.627(0.016)	0.806(0.012)	0.515(0.016)
KNN	0.856(0.014)	0.760(0.080)	0.610(0.052)	0.688(0.104)	0.853(0.115)	0.982(0.037)	0.478(0.040)	0.802(0.052)	0.315(0.066)

**Table 2:** Multi-model comparison, validation set results.

Model	AUC(SD)	cutoff(SD)	accuracy(SD)	sensitivity(SD)	specificity(SD)	positive predictive value (SD)	negative predictive value (SD)	F1 score (SD)	Kappa(SD)
XGBoost	0.743(0.130)	0.863(0.025)	0.647(0.150)	0.706(0.192)	0.853(0.225)	0.801(0.159)	0.511(0.135)	0.731(0.139)	0.309(0.275)
logistic	0.726(0.130)	0.656(0.018)	0.680(0.094)	0.838(0.143)	0.720(0.138)	0.800(0.089)	0.554(0.120)	0.813(0.099)	0.343(0.180)
LightGBM	0.746(0.126)	0.637(0.019)	0.703(0.101)	0.720(0.178)	0.844(0.181)	0.809(0.118)	0.561(0.126)	0.753(0.142)	0.367(0.227)
RandomForest	0.763(0.115)	0.623(0.048)	0.699(0.097)	0.707(0.150)	0.842(0.149)	0.844(0.075)	0.575(0.121)	0.760(0.101)	0.403(0.166)
AdaBoost	0.701(0.116)	0.504(0.001)	0.668(0.125)	0.712(0.170)	0.804(0.216)	0.766(0.115)	0.537(0.158)	0.727(0.120)	0.296(0.255)
GNB	0.737(0.112)	0.629(0.032)	0.703(0.092)	0.782(0.167)	0.782(0.128)	0.794(0.087)	0.612(0.186)	0.778(0.121)	0.362(0.196)
CNB	0.676(0.140)	0.495(0.001)	0.693(0.144)	0.736(0.253)	0.798(0.184)	0.742(0.101)	0.597(0.348)	0.725(0.178)	0.291(0.346)
SVM	0.737(0.115)	0.686(0.014)	0.689(0.085)	0.768(0.165)	0.789(0.136)	0.843(0.097)	0.551(0.108)	0.793(0.109)	0.375(0.165)
KNN	0.686(0.186)	0.760(0.080)	0.561(0.110)	0.540(0.254)	0.882(0.175)	0.896(0.172)	0.446(0.075)	0.629(0.239)	0.232(0.170)

### Interpretability of the metastasis model

**Figure 8:**
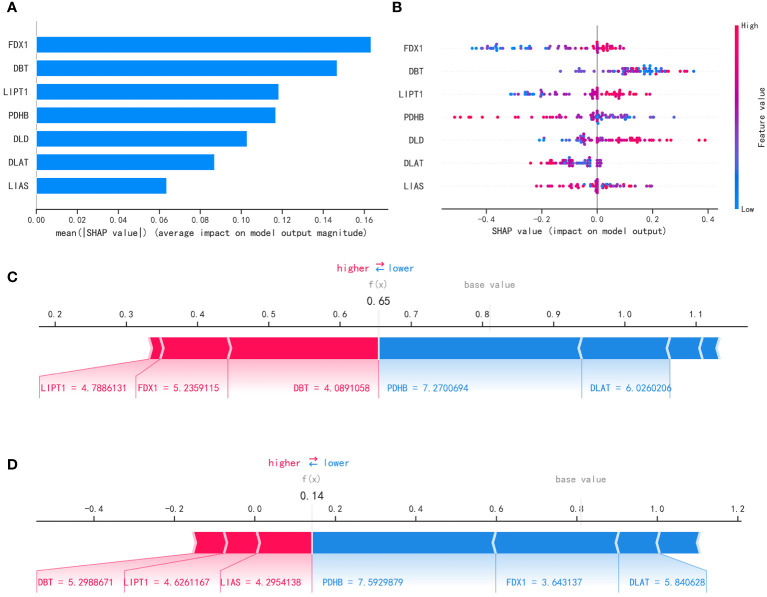
Interpretability of the metastasis model. **(A)** “SHAP” package to explain the importance of key variables to the model. **(B)** Contribution of each variable to the model. **(C, D)** Prediction of model.

After filtering out the best performing LightGBM model, we used the “SHAP” package to explain the importance of key variables to the model. As shown in **Figure 8A**, the importance of 7 variables from high to low is: ‘FDX1’, ‘ DBT’, ‘LIPT1’, ‘PDHB’, ‘DLD’, ‘DLAT’, ‘LIAS’. **Figure 8B** shows the contribution of each variable to the model. The red dots indicate positive contributions, and the blue dots indicate negative contributions. A point closer to the left indicates a smaller value and a point closer to the right indicates a larger value. For example, the higher the FDX1 value, the higher the probability of death from heart failure; the lower the FDX1 value, the lower the probability of heart failure death. At the same time, we use the force diagram to illustrate 2 samples to show how different variables contribute to different samples. **Figures 8C, D** show the model predicts that these two samples are likely to metastasize and not metastasize, respectively, and show the contribution of each gene’s expression to the sample prediction. Red indicates a positive contribution. Blue represents a negative contribution. If f(x) is greater than the cut-off value, the tumor sample is more likely to metastasize; if f(x) is less than the cut-off value, the tumor sample is less likely to metastasize.

### Knockdown of fdx1 inhibits the proliferation of melanoma cells

**Figure 9:**
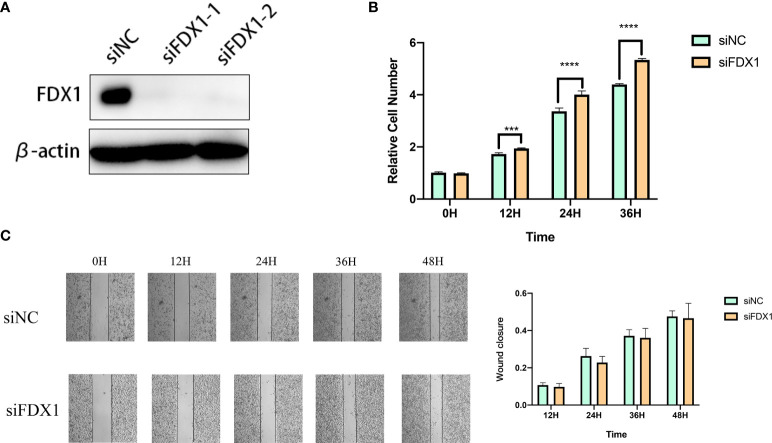
Knockdown of fdx1 inhibits the proliferation of melanoma cells. **(A)** FDX1-targeting siRNAs to knockdown the expression levels of FDX1. **(B)** The proliferation of FDX1 knockdown cells at 12 h, 24 h, and 36 h. **(C)** Wound healing assay at 12 h, 24 h, 36 h, and 48 h.

We used specific FDX1-targeting siRNAs to knockdown the expression levels of FDX1 in the A375 cells (**Figure 9A**). siNC was used as a control group for subsequent comparative analysis. CCK-8 assay results showed that the proliferation of FDX1 knockdown cells at 12h, 24h, and 36h was significantly higher than that of the control group (**Figure 9B**). Wound healing assay results showed that FDX1 knockdown inhibited wound healing (**Figure 9C**). siNC group healed slightly faster than the siFDX1 group. However, this result is not statistically significant.

## Discussion

As one of the deadliest tumors in skin cancer, melanoma is characterized by high invasiveness and high mortality (1). Therefore, a large body of literature has explored the prognosis and metastasis of melanoma. At present, the literature has predicted the prognosis of melanoma patients based on the expression levels of pyroptotic genes, tumor microenvironment status, or m6a-regulated methylation patterns (23–30). Although many bioinformatics studies are predictingognosis of melanoma, the existing cuproptosis-related melanoma research is not abundant.

Recently, Tsvetkov et al. discovered a novel apoptosis-independent cell death pathway, copper-dependent cell death (termed cuproptosis) (7). They proved that copper ions bind directly to the lipoylated components of the tricarboxylic acid cycle. Then, proteotoxic stress and unique cell death were induced. At the same time, the role performed by cuproptosis in tumours is gradually being understood. Zhong Hao et al. discovered that 6 CRGs had good diagnostic efficacy in kidney renal clear cell carcinoma (31). Besides, Liyang et al. developed a safe, mitochondria-targeted, copper-depleted nanoparticle (CDN) and tested its efficacy against triple-negative breast cancer (TNBC) (32). Injection CDN into mice with triple negative breast cancer resulted in a significant reduction in tumour growth and a significantly longer survival time for the mice. Zhang Zheng et al. constructed a prognostic model of HCC using the expression levels of ferredoxin 1 (FDX1) in hepatocellular carcinoma (HCC). They found that the expression level of FDX1 was significantly lower in HCC patients than in the non-HCC population (33). At the same time, survival time was significantly higher in patients with high expression of HCC than in those with low expression of HCC.These studies suggest that cuproptosis has implications for the clinical diagnosis and treatment of tumours.

Therefore, CRGs were used to construct molecular subtypes of melanoma and to construct metastasis models in this research. The molecular subtypes of melanoma based on CRGs can give us a more comprehensive understanding of melanoma. At the same time, the metastasis model established based on CRGs can also fill the gap in melanoma bioinformatics research.

In this study, we explored the effects of CRGs on both survival and metastasis in melanoma patients. We analyzed the expression of 12 CRGs in TCGA and GEO cohorts. First, through bioinformatics analysis, we constructed molecular subtypes of 3 CRGs (A_1_, B_1_, C_1_) based on 12 CRGs. Among the three molecular subtypes of CRGs, the C_1_ subtype had the best survival outcome, and the A_1_ subtype had the worst survival outcome. Next, we obtained 71 Differentially expressed genes that were co-expressed by all three subtypes. Based on 71 Differentially expressed genes, we genotyped melanoma and obtained 3 gene subtypes (A_2_, B_2_, C_2_). Among them, the B_2_ type had the best survival outcome, and the C_2_ type had the worst survival outcome. Then, we screened prognosis-related genes from 71 co-expressed Differentially expressed genes. After obtaining 16 prognosis-related genes, the LASSO algorithm was used to screen out 6 key variable genes(AIM2, EDNRB, SLC39A6, TMEM117, PTPRC, and KIF14) for model construction and validation. Ultimately, our risk score model can distinguish between high-risk and low-risk groups. And KM analysis, AUC analysis, nomogram, and calibration curve indicated that our model could predict the prognosis of melanoma patients more accurately. Finally, in the analysis of metastasis, we used the LightGBM machine learning algorithm to screen out 7 CRGs to establish the metastasis model of melanoma.

In the TCGA cohort, we found that 9 out of 13 CRGs had an impact on the prognosis of melanoma patients. Therefore, this sparked our interest in investigating the role of CRGs in melanoma prognosis and metastasis. The degree of immune cell infiltration was also significantly different among the three molecular subtypes of CRGs. We selected 23 immune cells for analysis, except for Eosinophilna cells, the other 22 immune cells had significantly different infiltration degrees in the three subtypes. This suggests that immune cells play different roles in different subtypes. In many tumors, the immune microenvironment plays an important role in tumor angiogenesis, tumor invasion, and metastasis. Patients with high expression of CXCL9, CXCL10, CXCL13, CCL4, and CCL5 in SKCM (Skin cutaneous melanoma) had better overall survival (27). Some studies have constructed risk models based on immune-related genes and found that immune cell infiltration is different between patients with high and low immune scores, and the survival time of patients with high immune scores is significantly lower than that of patients with low immune scores. Other studies have shown that in melanoma patients, IL27 is closely related to CD8+ cells, and is related to the treatment effect and prognosis of patients (34–36). Enrichment analysis of Metascape shows 71 intergenes were mainly enriched in MHC class II antigen presentation and platinum resistance pathways. Melanoma-specific MHC-II expression predicted anti-pd-1/PD-L1 treatment efficacy (37). Overexpression of BCL2L10 in melanoma has also been shown to promote cisplatin and ABT-737 resistance (38). In a case report, a patient with metastatic melanoma was also associated with hyperprolactinemia (39). Next, we used cox regression analysis and the LASSO algorithm to screen out 6 key variable genes(AIM2, EDNRB, SLC39A6, TMEM117, PTPRC, and KIF14) to construct a risk model. Absent in melanoma 2 (AIM2) is a cytoplasmic sensor that recognizes double-stranded DNA derived from viruses, bacteria, or the host itself, and is a member of the interferon inducible p200-protein (IFI-P200) family of immune-related proteins. AIM2 plays a significant role in autoimmune diseases (40) and the activation of inflammasome (41–44). In the melanoma-related literature, patients with melanoma whose dendritic cells express AIM2 have a significantly lower prognosis than patients with melanoma whose dendritic cells do not express AIM2 (45). In breast cancer treatment, Dihydroartemisinin induces pyroptosis in breast cancer cells by promoting the AIM2/caspase-3/DFNA5 (gasdermin E) axis (46). Endothelin Receptor type B (EDNRB) is widely expressed in vascular endothelial cells of the cardiovascular system, gastrointestinal tract, lung, kidney, adrenal gland, uterus, prostate, and brain. In melanoma-related studies, the prognostic value of patients with high CD8(+) T cell subpopulations expressing EDNRB was significantly reduced (47). This suggests that EDNRB could be a potential therapeutic target for melanoma. Solute carrier family 39 member 6(SLC39A6) is also known as LIV-1, ZIP-6, and Zinc transporter ZIP6. May act as a zinc-influx transporter. Solute Carrier Family 39 Member 6 (SLC39A6) is also known as LIV-1, ZIP-6, and Zinc transporter ZIP6. May act as a zinc-influx transporter. In studies of esophageal cancer, SLC39A6 increases the invasiveness of esophageal cancer cells and reduces patient prognosis by increasing the level of Zinc expression in esophageal cancer cells (48). SLC39A6 can also be used as an indicator for early diagnosis of esophageal cancer (49). However, in luminal breast cancer, the Oestrogen-regulated protein SLC39A6 acts as a benign prognostic indicator (50). One study reported that transmembrane protein 117 (TMEM117) was associated with endoplasmic reticulum stress-mediated mitochondrial-mediated cell death (51). Studies have shown that in primary liver cancer, miR-631 can target the receptor protein tyrosine phosphatase gene (PTPRE) to inhibit the intrahepatic metastasis of liver cancer (52). In kras mutant lung adenocarcinoma, the PTPRE is highly expressed, which can be used as a novel therapeutic target in kras mutant lung adenocarcinoma (53). The kinesin family member 14 (KIF14), is a novel oncogene located on chromosome 1q. When it malfunctions, it can affect the development of the brain and kidneys, and it can lead to many types of cancer (54, 55). In breast cancer, high expression of KIF14 can promote breast cancer metastasis and is associated with poor prognosis of breast cancer patients (56, 57). Similarly, studies have shown that in gastric cancer, when KIF14 mRNA is highly expressed, the prognosis is significantly lower than that with low KIF14 mRNA expression (58). However, the above two genes have not been deeply studied in melanoma research, and the specific functions of PTPRE and KIF14 in melanoma need to be further explored.

Among these CRGs, we also screened out 7 key genes (FDX1, DBT, LIPT1, PDHB, DLD, DLAT, LIAS) as variables in the metastasis model. In other tumor metastasis models, the roles of some of these genes in tumor metastasis have also been found. Chen found that LIPT1 may be a prognostic-related gene for bladder cancer, and then found that this gene has a certain degree of inhibitory effect on the migration ability of bladder cancer cells by transwell method (59). Zhao found that PDHB is associated with ovarian cancer growth and metastasis, and miR-203 can target the 3’-UTR of PDHB to promote glycolysis. Meanwhile, overexpression of PDHB could abolish the promoting effect of miR-203 on ovarian cancer cell growth (60). Regarding the role of these genes in tumor metastasis, we still need further functional tests to verify.

During the occurrence and development of tumor tissue, there are a large number of gene mutations. Mutated genes can provide tumor antigens that can be recognized by the immune system as non-self tissues, inducing immune cells to respond (61). Immunotherapy takes advantage of the fact that immune cells can recognize and eliminate tumor cells, which plays a great role in the treatment of tumors (62, 63). However, tumors effectively suppress immune responses (immune escape) by activating negative regulatory pathways associated with immune homeostasis (checkpoints) or by adopting features that allow them to actively evade detection (64, 65). Effective immunotherapy drugs have been approved in preclinical and clinical phase I-III trials for highly aggressive, highly refractory, and advanced and metastatic melanoma (66). For example, the anti-PD-1 monoclonal antibodies nivolumab and pembrolizumab and the anti-CTLA-4 antibody ipilimumab are being tested in clinical trials to treat melanoma (67). Studies have shown that commonly used immune checkpoint inhibitors (ICIs) can improve progression-free survival and overall survival in melanoma patients (68, 69). In our study, the risk score model showed that the degree of immune cell infiltration in the high-risk group was significantly lower than that in the low-risk group. Interestingly, the survival time of the high-risk group was significantly lower than that of the low-risk group. In other melanoma studies, the survival time of the high immune score group was significantly higher than that of the low immune score group (70, 71). We also propose a hypothesis here, in melanoma, is the degree of immune cell infiltration positively correlated with the survival time of patients? This problem also needs more clinical data or experiments to confirm. It is worth mentioning that in this study, we also analyzed the drug sensitivity between high and low risk groups. We screened 98 drugs (**Supplementary File 1**) with significant differences in IC50 concentrations between high and low risk groups. Among them, worthy of our attention are Sunitinib, VX-702, and Bryostatin.1. Sunitinib is a new class of drugs that can selectively target multiple receptor tyrosine kinases, and is now being used alone or in combination with other antitumor drugs to treat many solid tumors, including liver cancer, renal cancer, and gastric cancer (72–74). VX-702 is a highly selective p38α MAPK inhibitor targeting nimokinase for the treatment of primary and acquired endocrine-resistant breast cancer (75). Bryostatin-1 is a protein kinase C (PKC) inhibitor that inhibits cell entry into mitosis, lowers pH and energy metabolism, and reduces tumor blood flow, thereby inhibiting tumor cell growth (76, 77). We screened 98 drugs to guide the development of melanoma drugs.

Furthermore, our *in vitro* experiments showed that FDX1 promoted the growth, and migration of melanoma cells. Therefore, we speculate that FDX1, as a CRG, is a marker of melanoma. People with high expression of this gene need to be more alert to the occurrence of melanoma. At the same time, it is also a prognostic marker for melanoma patients. The prognosis of cancer patients may be better than that of other patients. Taken together, our results suggest that FDX1 is aberrantly expressed in melanoma and may be associated with patient prognosis. In the future, we need to conduct more in-depth functional experiments to explore how this gene acts on the occurrence and development of melanoma.

This study established prognostic and metastatic models of CRGs in melanoma. But there are still some limitations. Although the sample size of our sequencing data is relatively large, it is mainly based on the data of the network database, and we also need our own sequencing data to verify. In our *in vitro* experiments, knockdown of FDX1 reduced the ability of cells to migrate, but there was no difference compared to the control group. However, this gene was selected in our model, which may be due to the joint effect of multiple genes in the establishment of the metastasis model. In the future, we will conduct more in-depth experiments to explore its transfer mechanism. We analyzed the enriched pathways and functions of these key genes, and functional assays are needed to verify them. Finally, the drugs we screened also need to be verified by drug resistance experiments.

## Conclusion

In this study, melanoma was classified based on 12 CRGs and clinical features, and three subtypes, A_1_, B_1_, and C_1_, were established. Among them, the C_1_ subtype had the best survival outcome and the highest immune cell infiltration. Then, A_2_, B_2_, and C_2_ subtypes were established based on genotyping, with the B_2_ subtype having the best survival outcome. We performed functional analysis on the intergenes between different types, and the results showed that these intergenes were mainly enriched in cell cycle and drug metabolism pathways. We also established a prognostic model using 6 key variable genes and analyzed the tumor microenvironment according to the high and low risk scores of prognosis. In addition, we screened drugs for high and low risk groups and found that 98 drugs had significant differences in IC50 concentrations in high and low risk groups. Finally, we used the LightGBM algorithm to screen out 7 CRGs to build the transfer model of melanoma. These results help us to understand the role of CRGs in the occurrence and development of melanoma, and provide us with new therapeutic ideas and potential treatment methods.

## Data availability statement

The original contributions presented in the study are included in the article/**Supplementary Material**. Further inquiries can be directed to the corresponding author.

## Author contributions

JL was responsible for the study concept and design. Y-WL and FL revised the manuscript and made final approval of the version. JL, ZL, and LL analyzed the data. ZL, JL, and LL helped with the experimental section. LL helped to write the manuscript. All authors contributed to the article and approved the submitted version.

## Funding

This work was supported by National Key R&D Program of China (No.2020YFC2005000), the National Natural Science Foundation of China (No. 81802668, 82172832), the Wisdom Accumulation and Talent Cultivation Project of the Third Xiangya Hospital of Central South University (YX202108), and the Postgraduate Research and Innovation Project of Central South University (No.2020zzts892).

## Acknowledgments

The authors would like to acknowledge the GEO databases (https://www.ncbi.nlm.nih.gov/gds/) and TCGA database (https://tcga-data.nci.nih.gov/tcga/) for providing their platforms and those contributors for uploading their valuable datasets. And we also thanks for Extreme Smart Analysis (https://www.xsmartanalysis.com/) for providing the platforms to analyse our metastasis model.

## Conflict of interest

The authors declare that the research was conducted in the absence of any commercial or financial relationships that could be construed as a potential conflict of interest.

## Publisher’s note

All claims expressed in this article are solely those of the authors and do not necessarily represent those of their affiliated organizations, or those of the publisher, the editors and the reviewers. Any product that may be evaluated in this article, or claim that may be made by its manufacturer, is not guaranteed or endorsed by the publisher.

## Supplementary material

Supplementary Figure 1 Mutations and copy number variations of CRGs in the TCGA cohort. **(A)** The proportion of mutation frequency of CRGs in melanoma. **(B)** The chromosome where the mutated CRGs are located. Red represents an increase in copy number and green represents a decrease in copy number. **(C)** CRGs copy number variation graph. The ordinate of the red circle is the number of samples with increased copy number, and the ordinate of the green circle is the number of samples with reduced copy number.

Supplementary Figure 2 9 CRGs associated with prognosis. **(A–I)** Comparison of the overall survival time of samples with high expression of CRGs genes (indicated in red) and samples with low expression of CRGs (indicated in blue).

Supplementary Figure 3 Melanoma gene subtypes constructed based on prognostic-related intersection differentially expressed genes. **(A)** Three gene subtypes obtained by unsupervised consensus clustering method. **(B)** Consistent distribution of different K values described by a consistent cumulative distribution function (CDF) plot. **(C)** The delta area score displayed the relative growth in cluster stability. **(D)** Kaplan Meier analysis results of three gene subtypes. **(E)** Comparison of CRGs expression levels among the three gene subtypes. **(F)** Heatmap showing clinical information and gene expression profiles for the three gene subtypes.

Supplementary Table 1 Names of 13 cuproptosis-related genes

Supplementary File 1 98 drugs were with significant differences in IC50 concentrations between high and low risk groups.
