# Supplementary figures and images for: The role of cuproptosis-related gene in the classification and prognosis of melanoma
Source: Front Immunol. 2022 Oct 19;13:986214. doi: 10.3389/fimmu.2022.986214 (PMC9632664; doi:10.3389/fimmu.2022.986214)

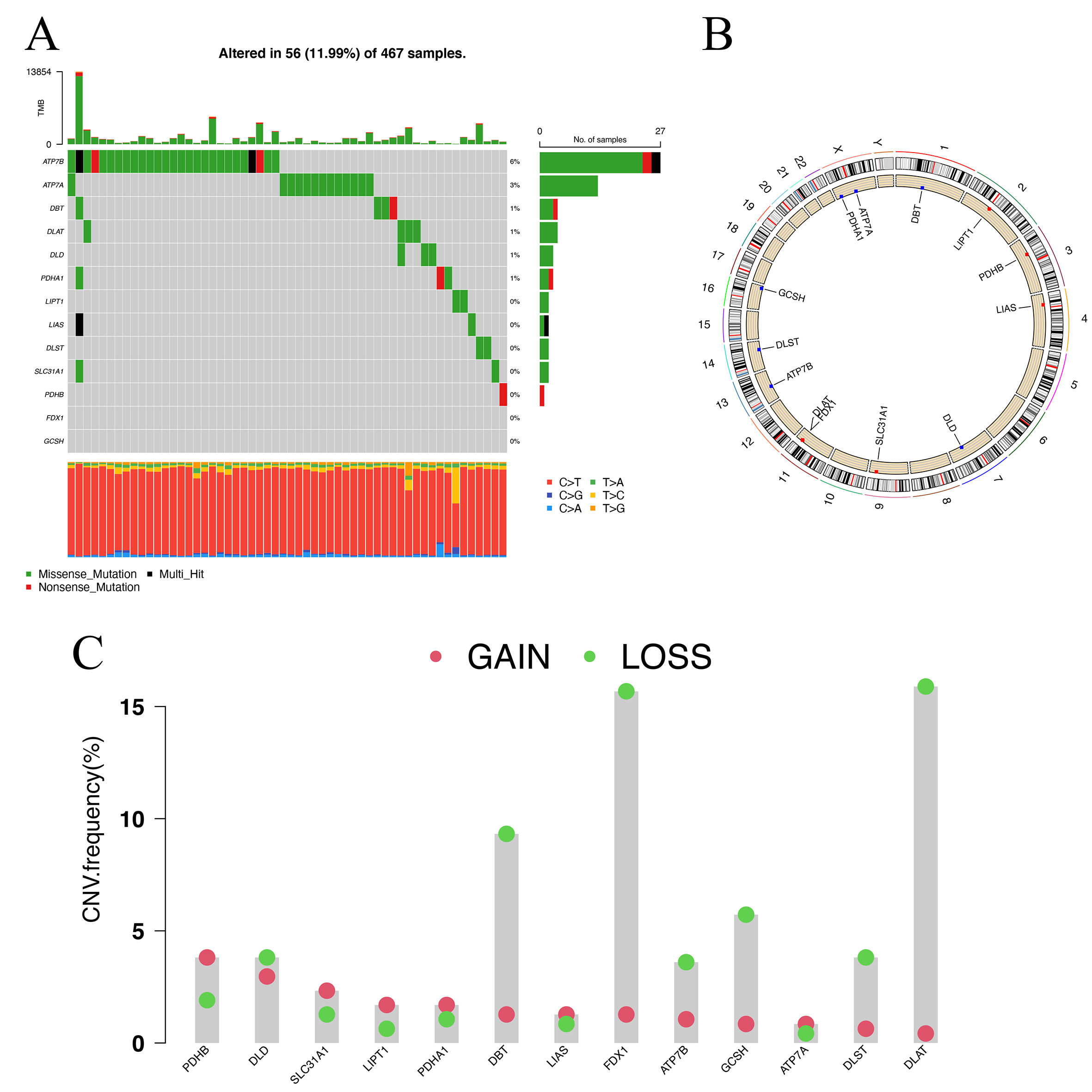

Supplement: Supplementary Figure 1 — Mutations and copy number variations of CRGs in the TCGA cohort. (A) The proportion of mutation frequency of CRGs in melanoma. (B) The chromosome where the mutated CRGs are located. Red represents an increase in copy number and green represents a decrease in copy number. (C) CRGs copy number variation graph. The ordinate of the red circle is the number of samples with increased copy number, and the ordinate of the green circle is the number of samples with reduced copy number. [file Image_1.jpeg]

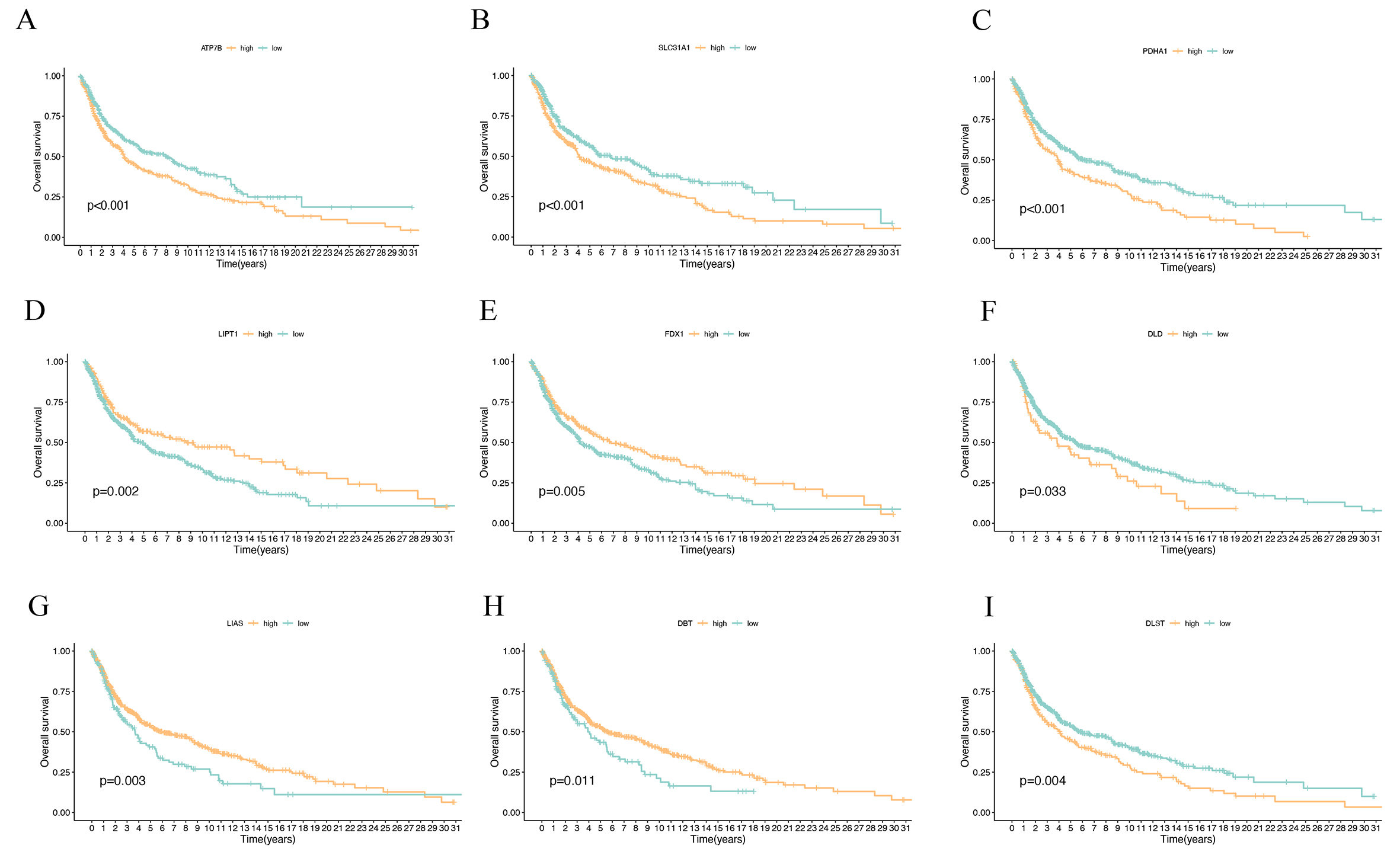

Supplement: Supplementary Figure 2 — 9 CRGs associated with prognosis. (A–I) Comparison of the overall survival time of samples with high expression of CRGs genes (indicated in red) and samples with low expression of CRGs (indicated in blue). [file Image_2.jpeg]

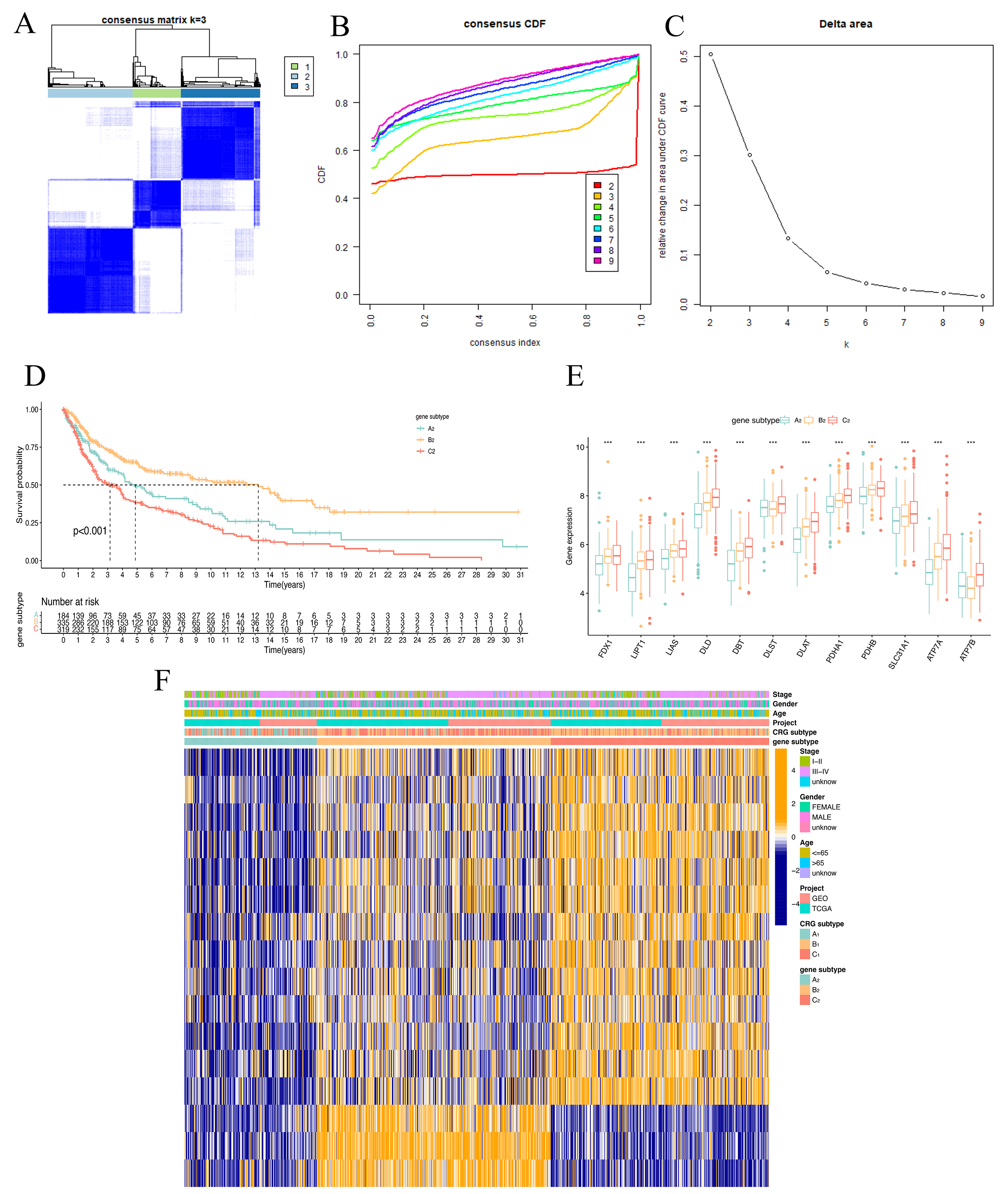

Supplement: Supplementary Figure 3 — Melanoma gene subtypes constructed based on prognostic-related intersection differentially expressed genes. (A) Three gene subtypes obtained by unsupervised consensus clustering method. (B) Consistent distribution of different K values described by a consistent cumulative distribution function (CDF) plot. (C) The delta area score displayed the relative growth in cluster stability. (D) Kaplan Meier analysis results of three gene subtypes. (E) Comparison of CRGs expression levels among the three gene subtypes. (F) Heatmap showing clinical information and gene expression profiles for the three gene subtypes. [file Image_3.jpeg]

Risk 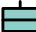 low 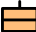 high

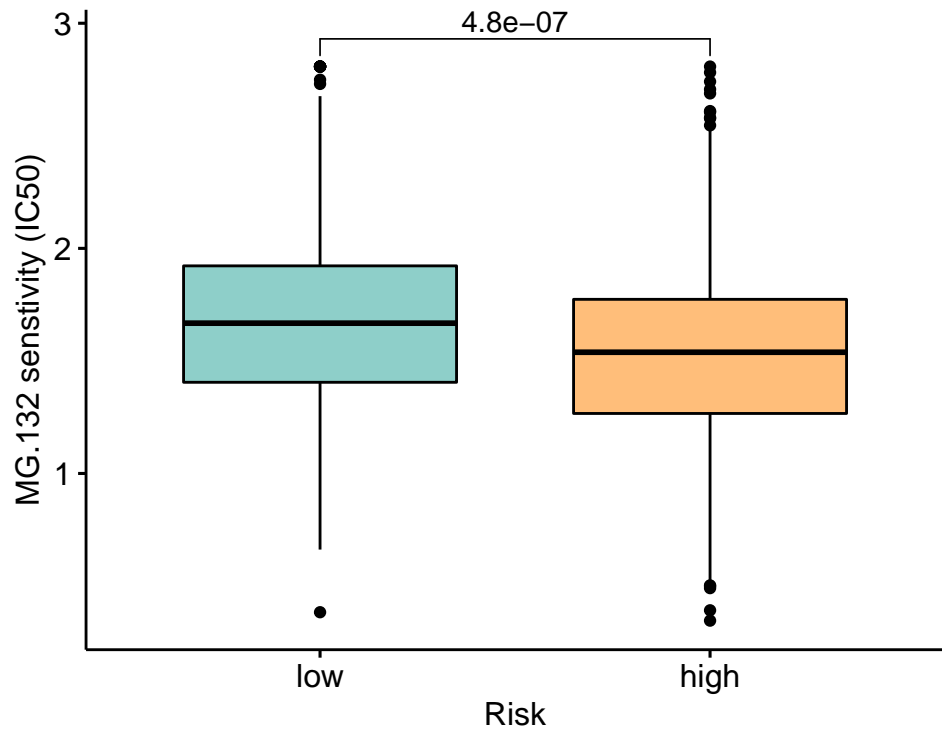

Supplement: Supplementary File 1 — 98 drugs were with significant differences in IC50 concentrations between high and low risk groups. [file DataSheet_1.zip › 1.durgSenstivity/durgSenstivity.MG.132.pdf]

Risk 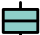 low 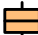 high

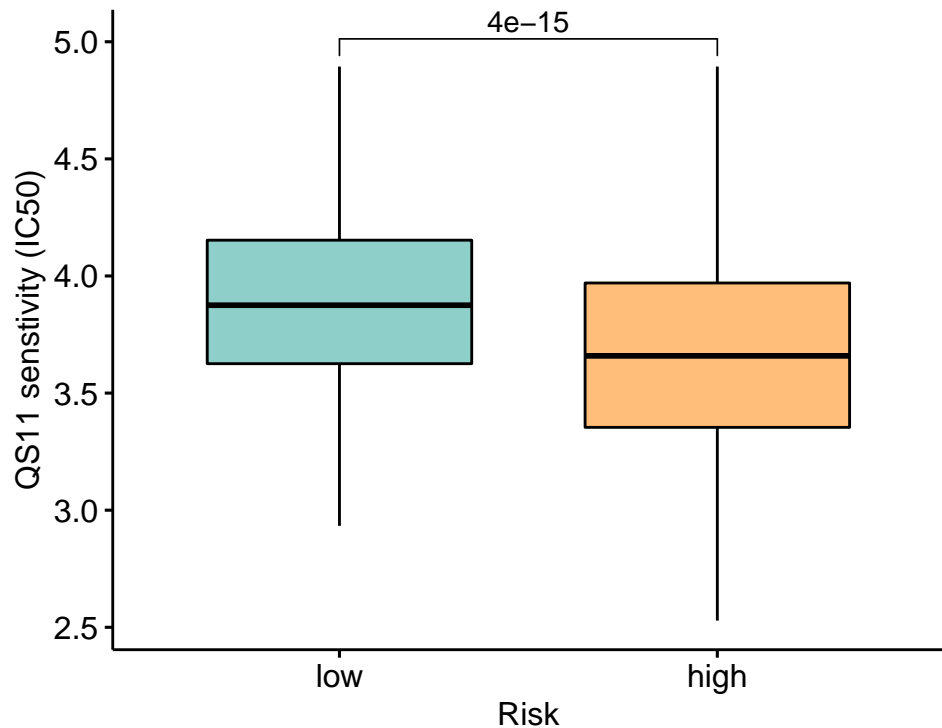

Supplement: Supplementary File 1 — 98 drugs were with significant differences in IC50 concentrations between high and low risk groups. [file DataSheet_1.zip › 1.durgSenstivity/durgSenstivity.QS11.pdf]

Risk 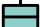 low 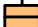 high

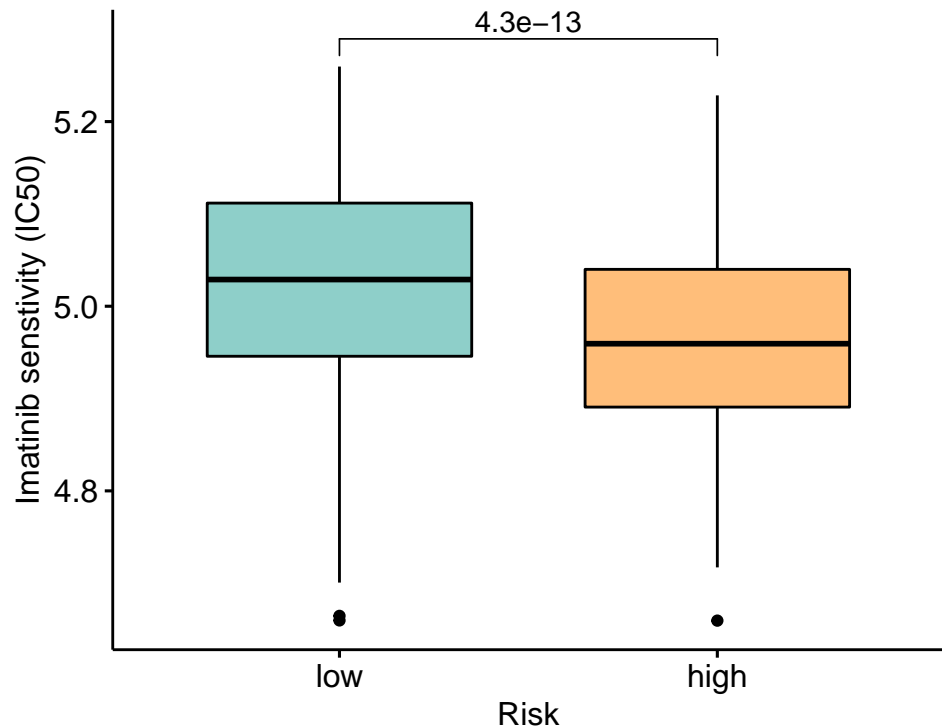

Supplement: Supplementary File 1 — 98 drugs were with significant differences in IC50 concentrations between high and low risk groups. [file DataSheet_1.zip › 1.durgSenstivity/durgSenstivity.Imatinib.pdf]

Risk 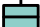 low 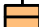 high

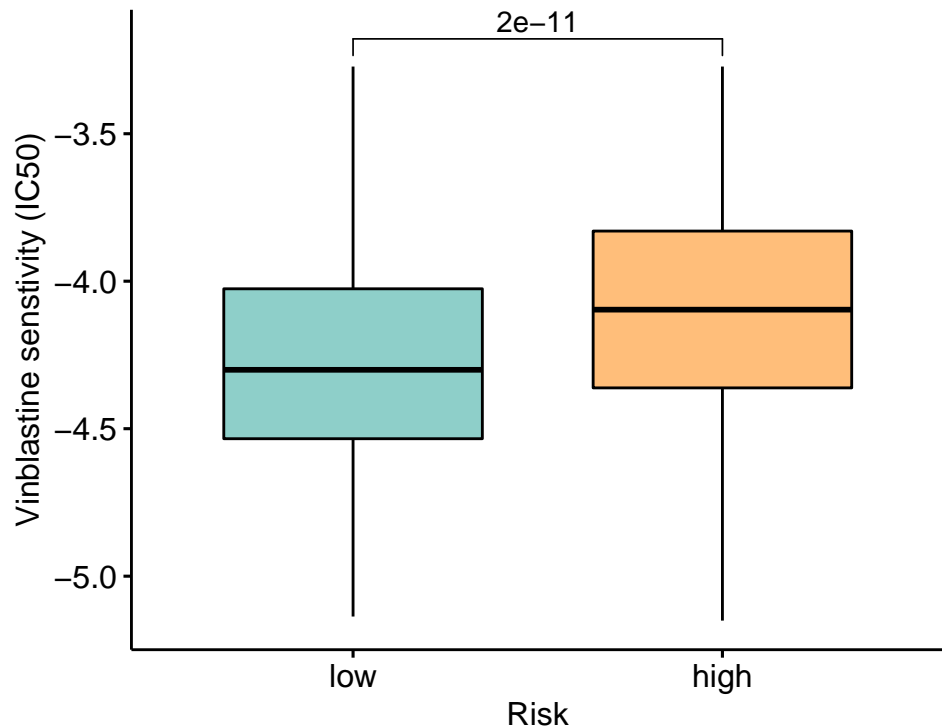

Supplement: Supplementary File 1 — 98 drugs were with significant differences in IC50 concentrations between high and low risk groups. [file DataSheet_1.zip › 1.durgSenstivity/durgSenstivity.Vinblastine.pdf]

Risk 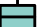 low 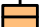 high

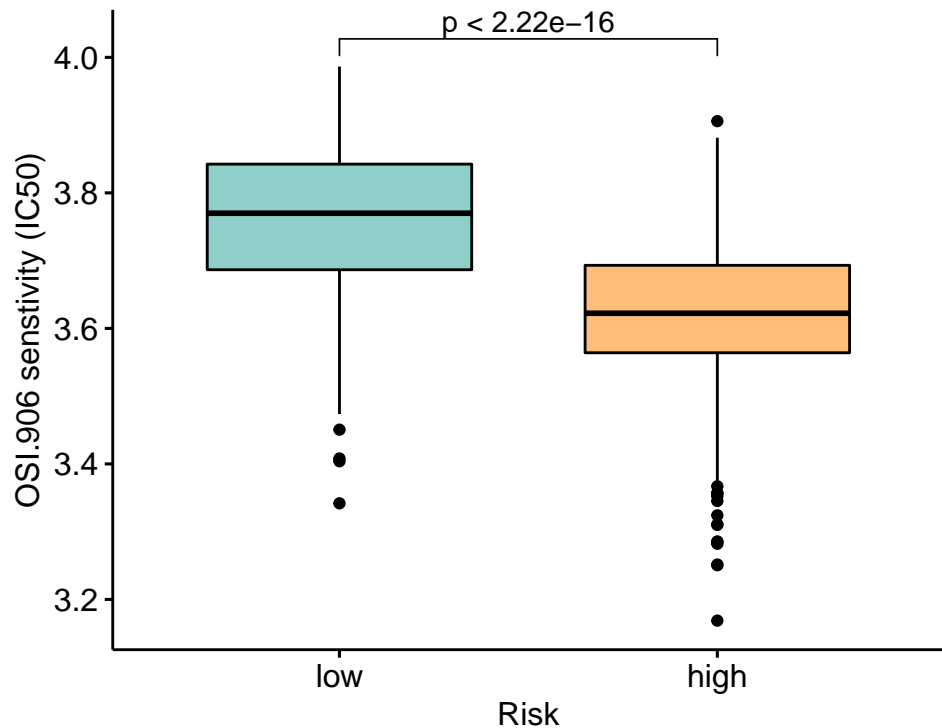

Supplement: Supplementary File 1 — 98 drugs were with significant differences in IC50 concentrations between high and low risk groups. [file DataSheet_1.zip › 1.durgSenstivity/durgSenstivity.OSI.906.pdf]

Risk 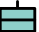 low 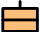 high

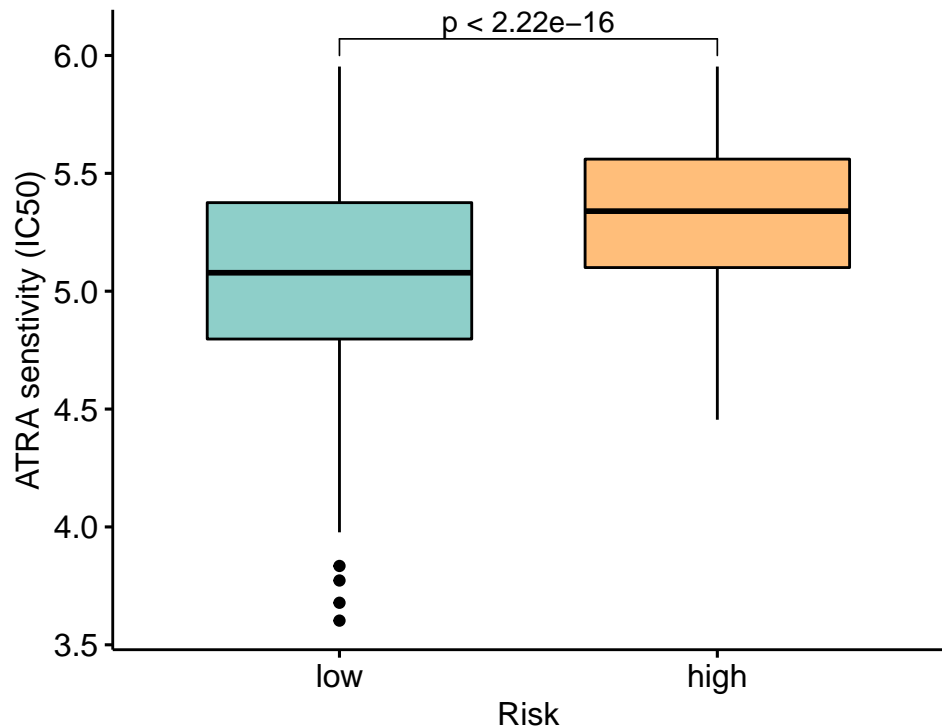

Supplement: Supplementary File 1 — 98 drugs were with significant differences in IC50 concentrations between high and low risk groups. [file DataSheet_1.zip › 1.durgSenstivity/durgSenstivity.ATRA.pdf]

Risk 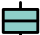 low 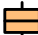 high

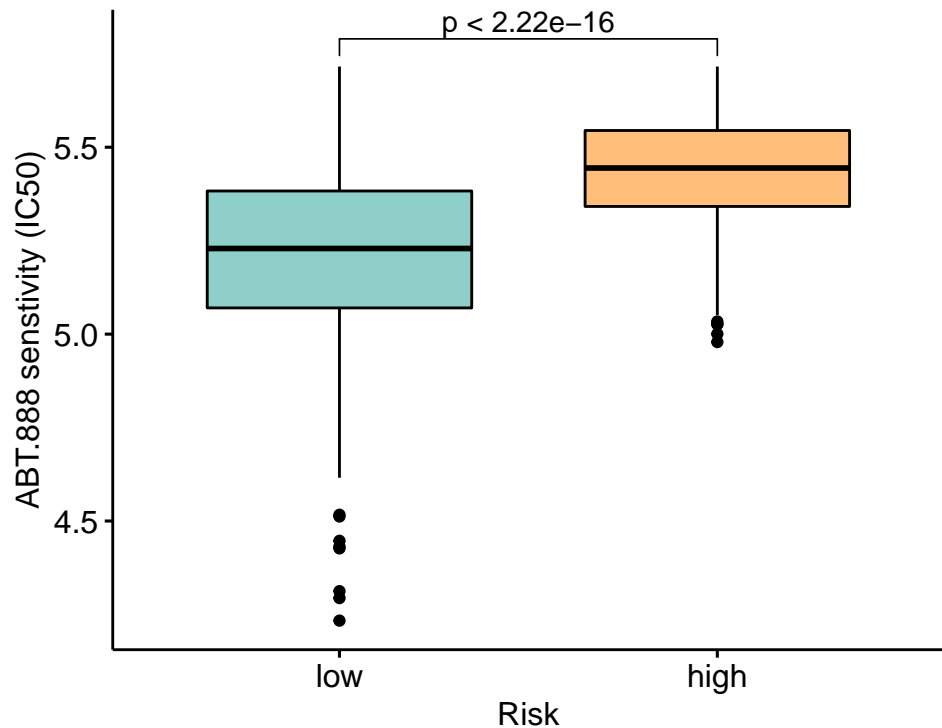

Supplement: Supplementary File 1 — 98 drugs were with significant differences in IC50 concentrations between high and low risk groups. [file DataSheet_1.zip › 1.durgSenstivity/durgSenstivity.ABT.888.pdf]

Risk 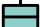 low 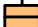 high

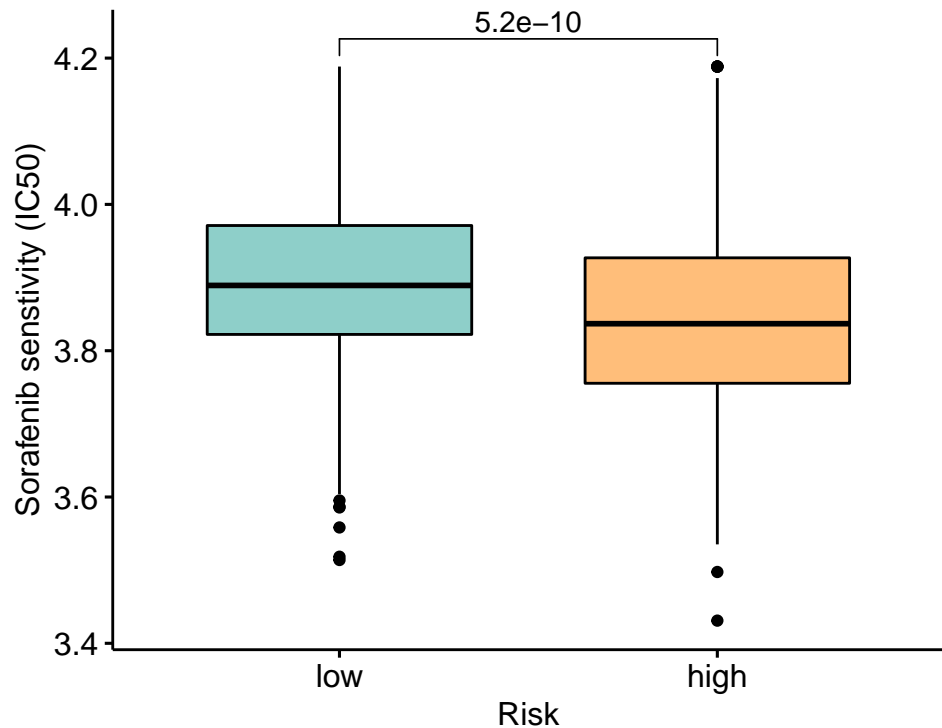

Supplement: Supplementary File 1 — 98 drugs were with significant differences in IC50 concentrations between high and low risk groups. [file DataSheet_1.zip › 1.durgSenstivity/durgSenstivity.Sorafenib.pdf]

Risk 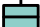 low 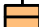 high

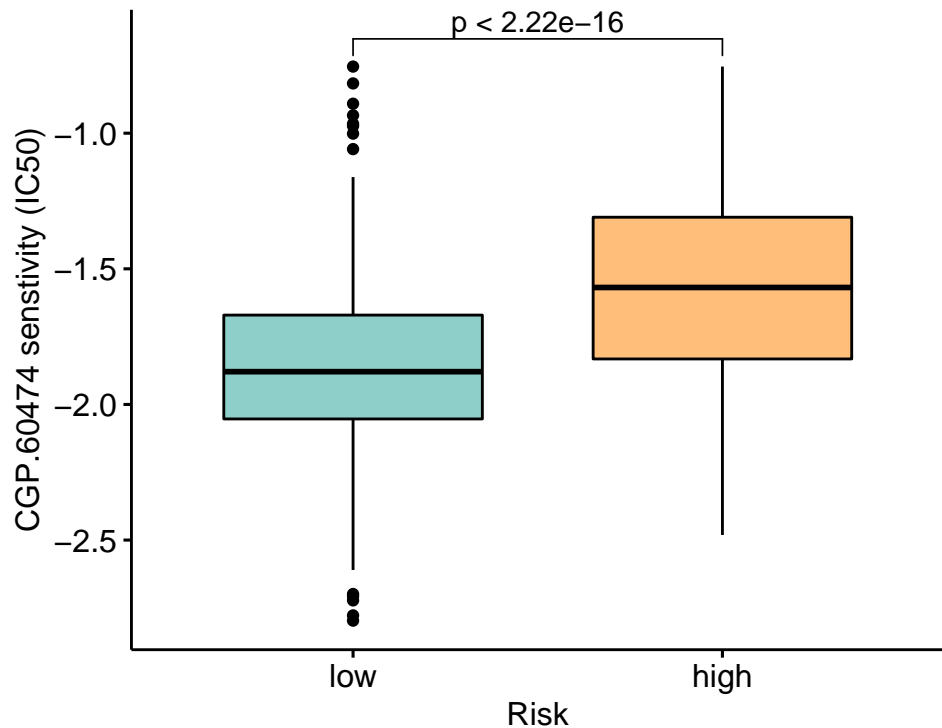

Supplement: Supplementary File 1 — 98 drugs were with significant differences in IC50 concentrations between high and low risk groups. [file DataSheet_1.zip › 1.durgSenstivity/durgSenstivity.CGP.60474.pdf]

Risk 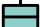 low 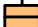 high

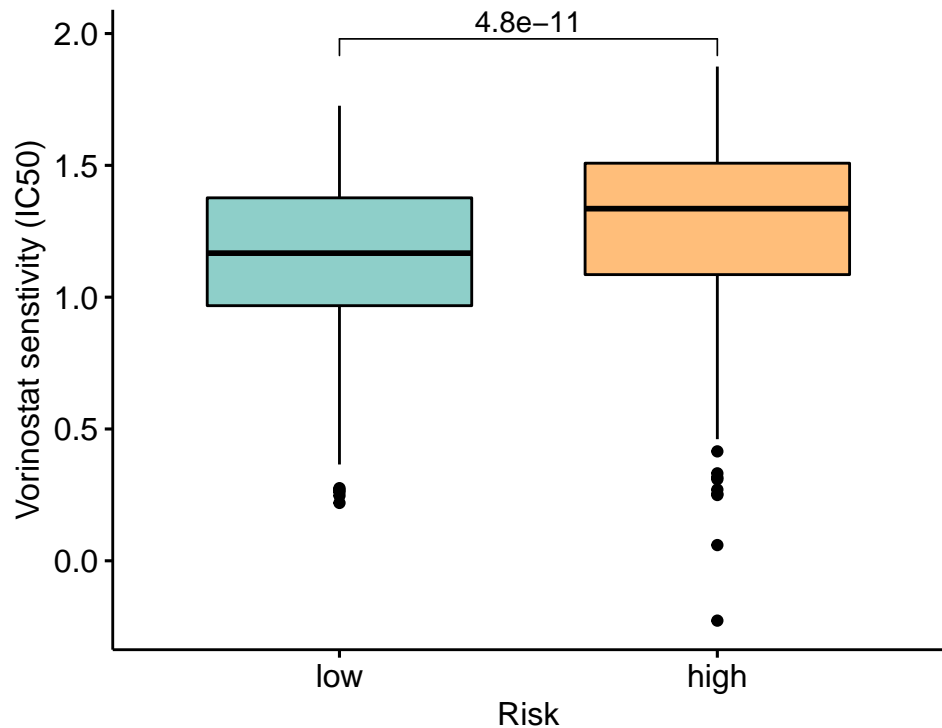

Supplement: Supplementary File 1 — 98 drugs were with significant differences in IC50 concentrations between high and low risk groups. [file DataSheet_1.zip › 1.durgSenstivity/durgSenstivity.Vorinostat.pdf]

Risk 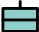 low 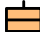 high

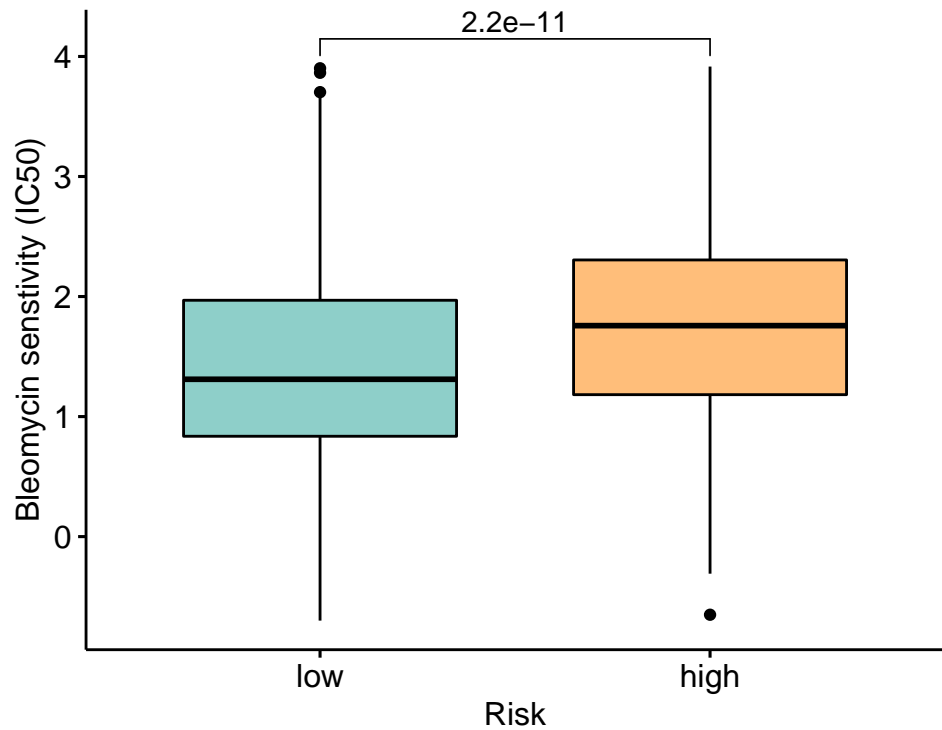

Supplement: Supplementary File 1 — 98 drugs were with significant differences in IC50 concentrations between high and low risk groups. [file DataSheet_1.zip › 1.durgSenstivity/durgSenstivity.Bleomycin.pdf]

IPA.3 sensitivity (IC50)

Risk 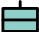 low 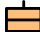 high

$2.6e-13$

low

high

Risk

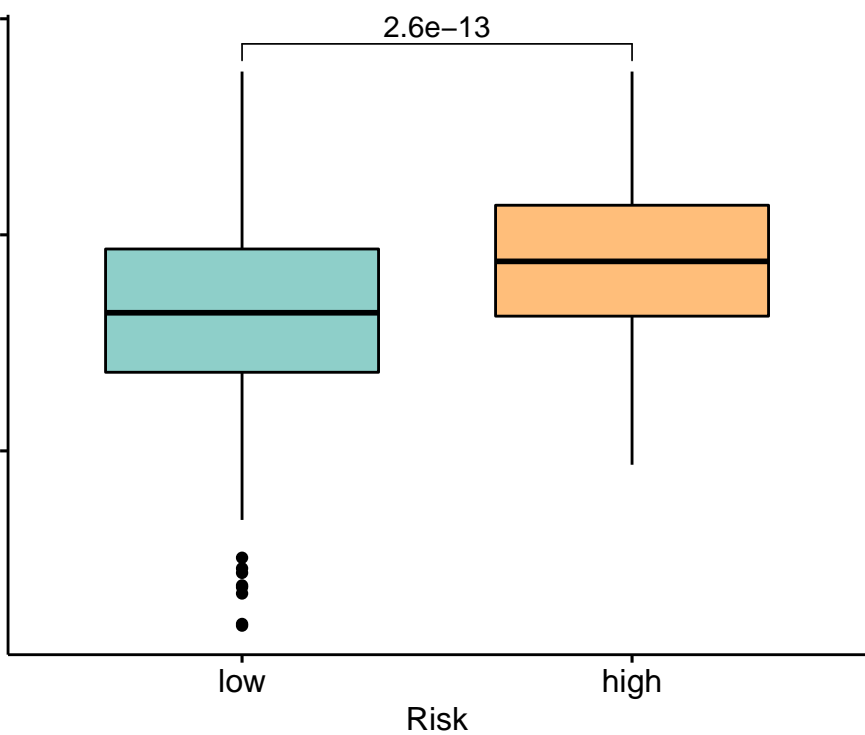

Supplement: Supplementary File 1 — 98 drugs were with significant differences in IC50 concentrations between high and low risk groups. [file DataSheet_1.zip › 1.durgSenstivity/durgSenstivity.IPA.3.pdf]

Risk 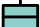 low 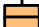 high

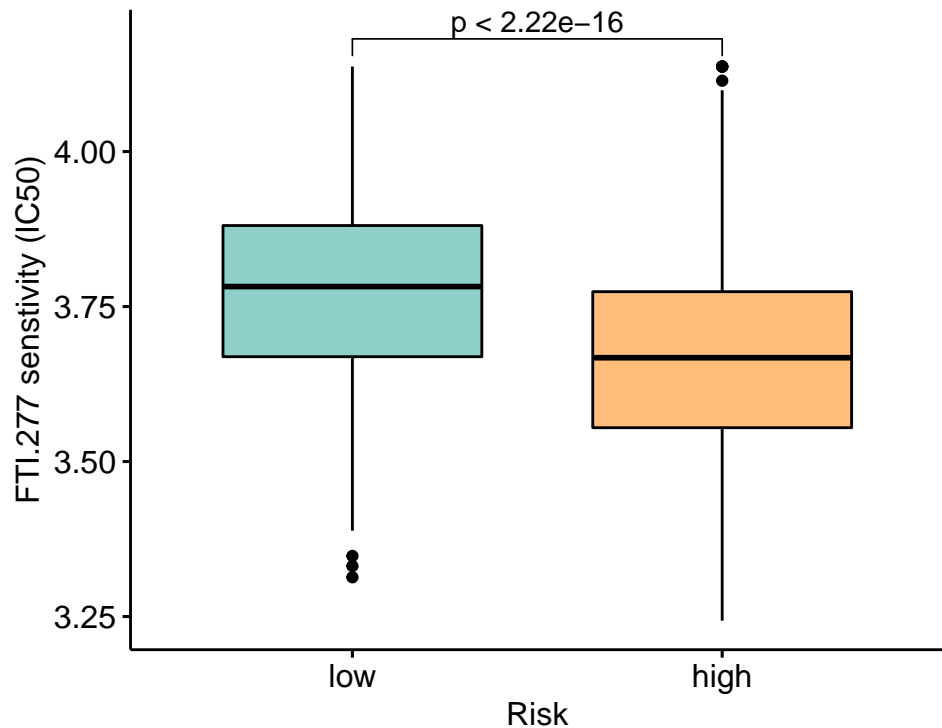

Supplement: Supplementary File 1 — 98 drugs were with significant differences in IC50 concentrations between high and low risk groups. [file DataSheet_1.zip › 1.durgSenstivity/durgSenstivity.FTI.277.pdf]

Risk 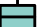 low 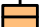 high

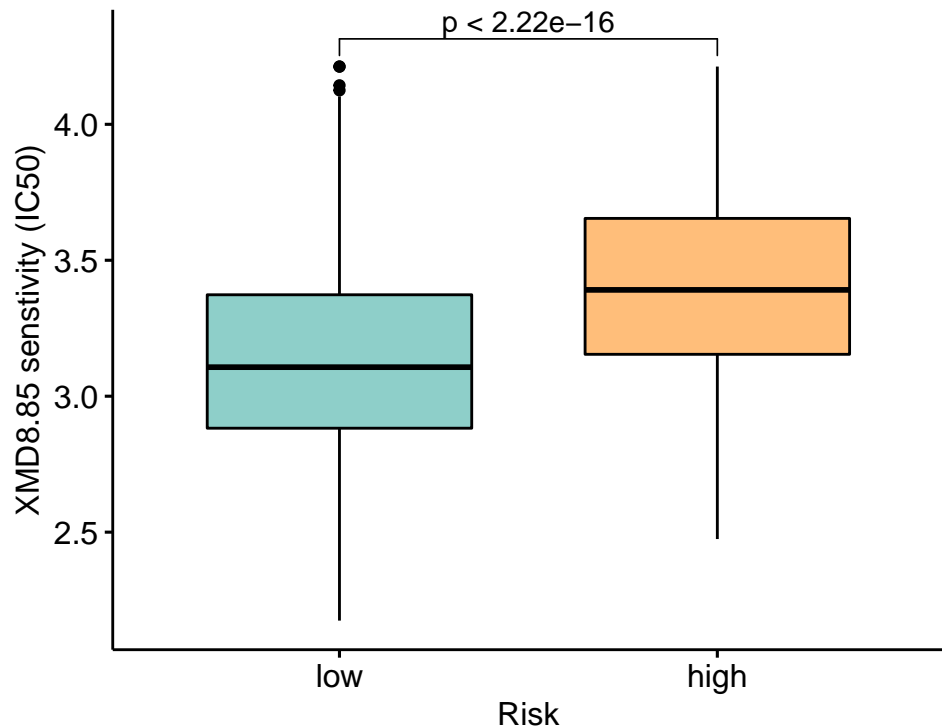

Supplement: Supplementary File 1 — 98 drugs were with significant differences in IC50 concentrations between high and low risk groups. [file DataSheet_1.zip › 1.durgSenstivity/durgSenstivity.XMD8.85.pdf]

Risk 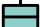 low 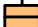 high

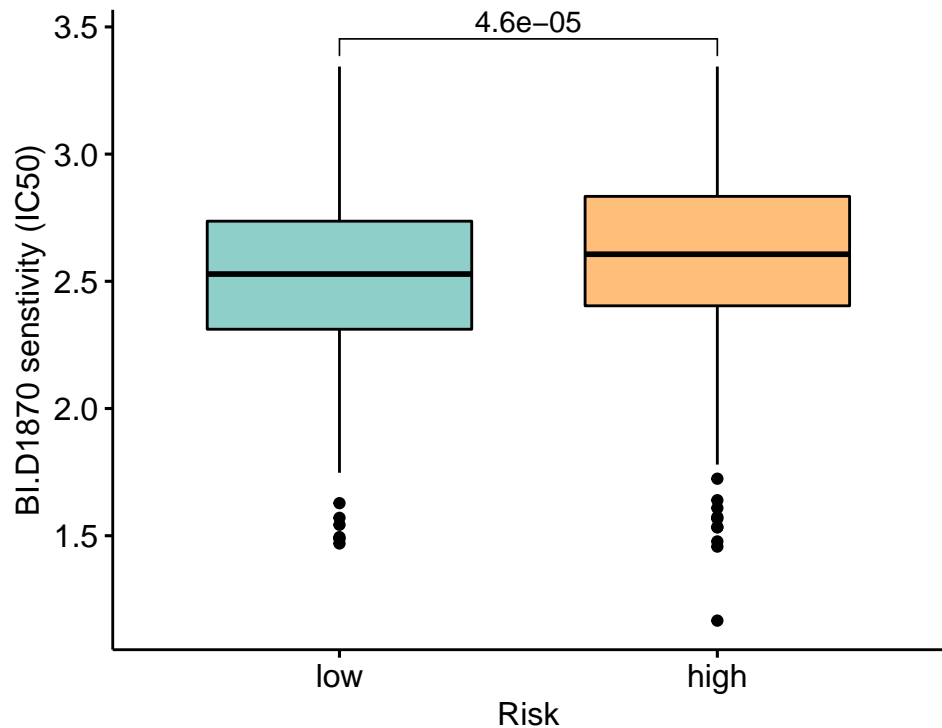

Supplement: Supplementary File 1 — 98 drugs were with significant differences in IC50 concentrations between high and low risk groups. [file DataSheet_1.zip › 1.durgSenstivity/durgSenstivity.BI.D1870.pdf]

Risk 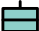 low 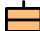 high

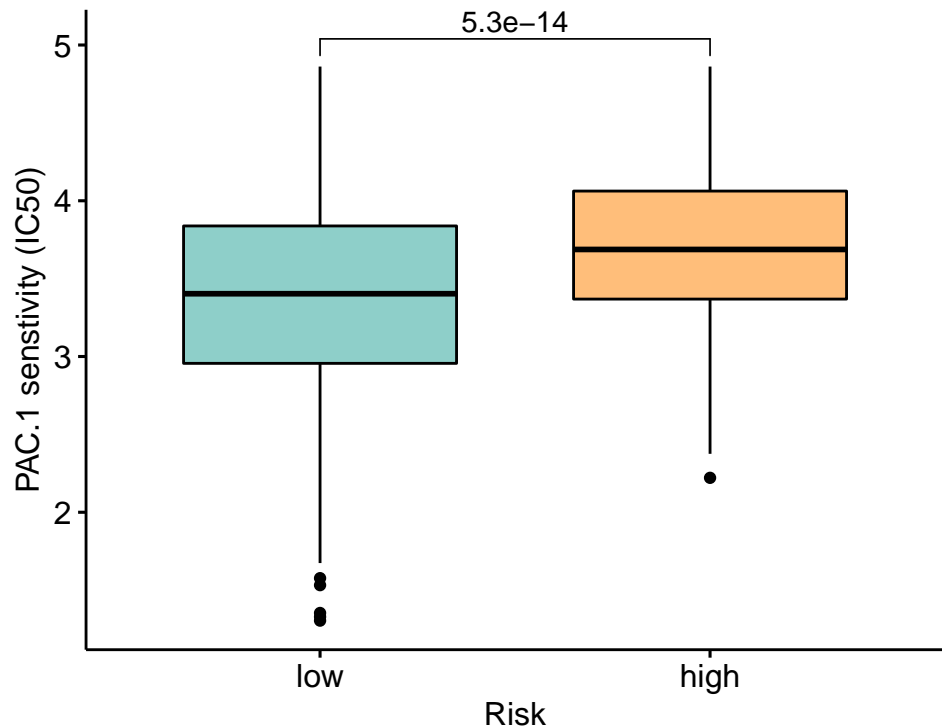

Supplement: Supplementary File 1 — 98 drugs were with significant differences in IC50 concentrations between high and low risk groups. [file DataSheet_1.zip › 1.durgSenstivity/durgSenstivity.PAC.1.pdf]

Risk 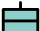 low 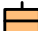 high

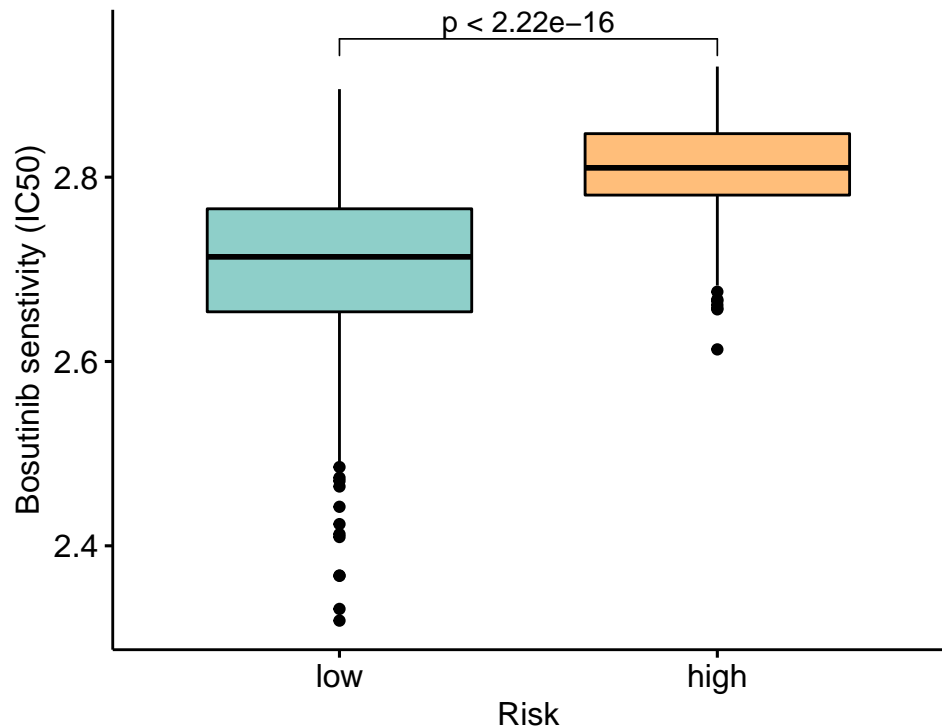

Supplement: Supplementary File 1 — 98 drugs were with significant differences in IC50 concentrations between high and low risk groups. [file DataSheet_1.zip › 1.durgSenstivity/durgSenstivity.Bosutinib.pdf]

Risk low high

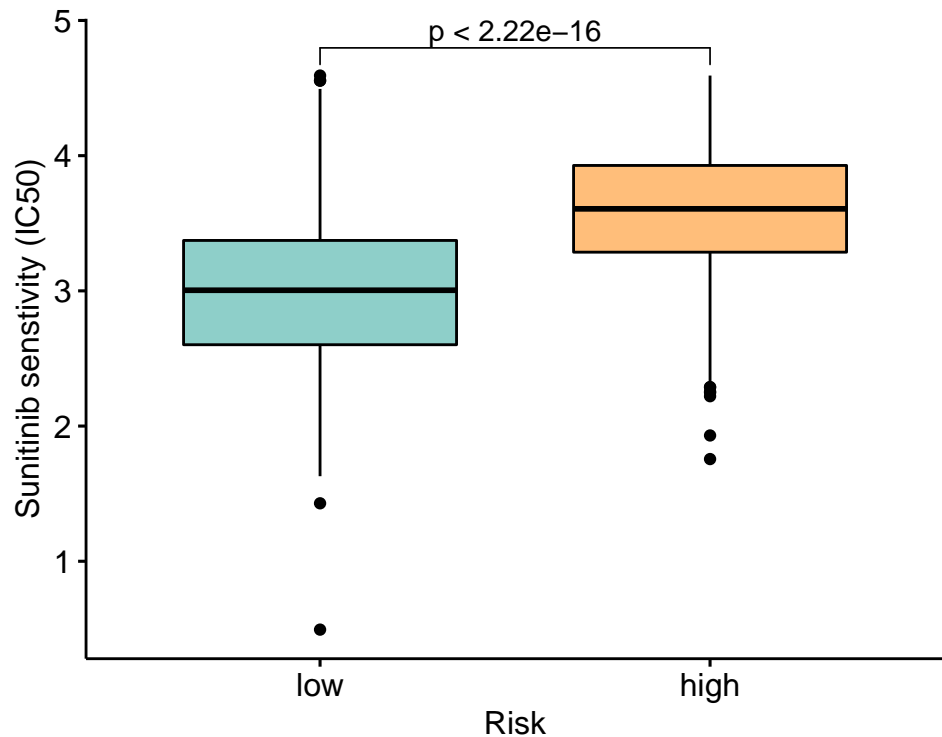

Supplement: Supplementary File 1 — 98 drugs were with significant differences in IC50 concentrations between high and low risk groups. [file DataSheet_1.zip › 1.durgSenstivity/durgSenstivity.Sunitinib.pdf]

Risk 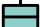 low 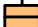 high

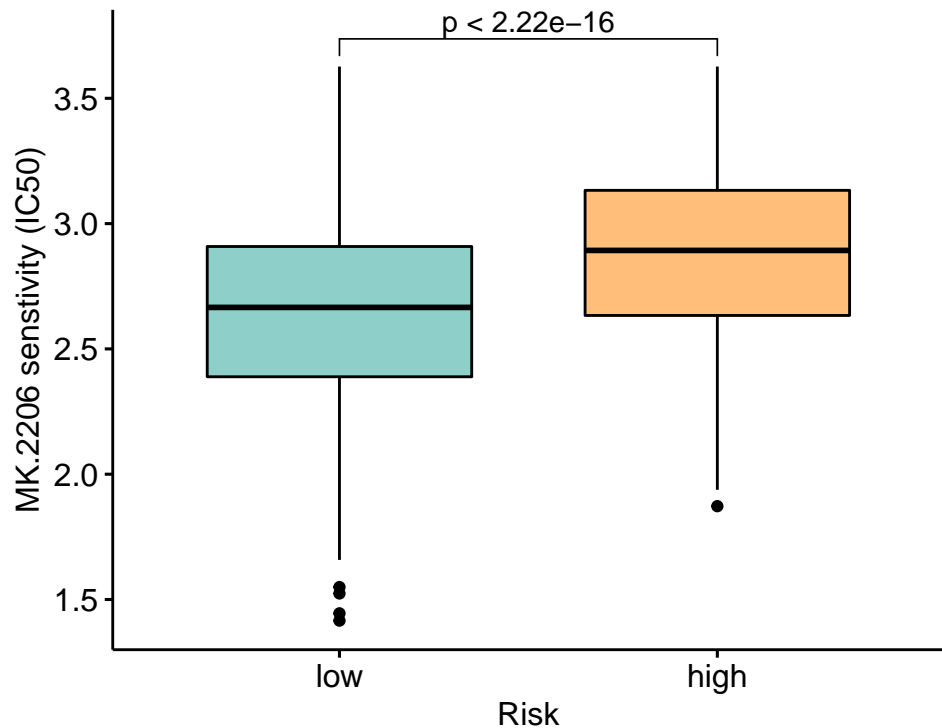

Supplement: Supplementary File 1 — 98 drugs were with significant differences in IC50 concentrations between high and low risk groups. [file DataSheet_1.zip › 1.durgSenstivity/durgSenstivity.MK.2206.pdf]

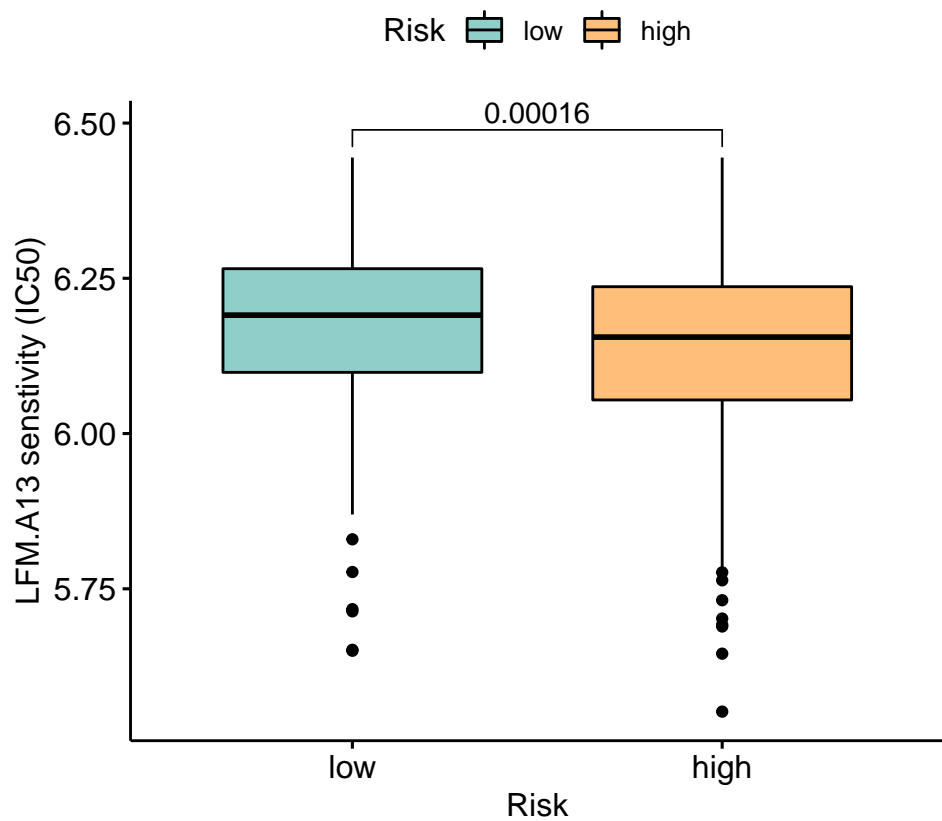

Supplement: Supplementary File 1 — 98 drugs were with significant differences in IC50 concentrations between high and low risk groups. [file DataSheet_1.zip › 1.durgSenstivity/durgSenstivity.LFM.A13.pdf]

Risk 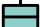 low 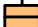 high

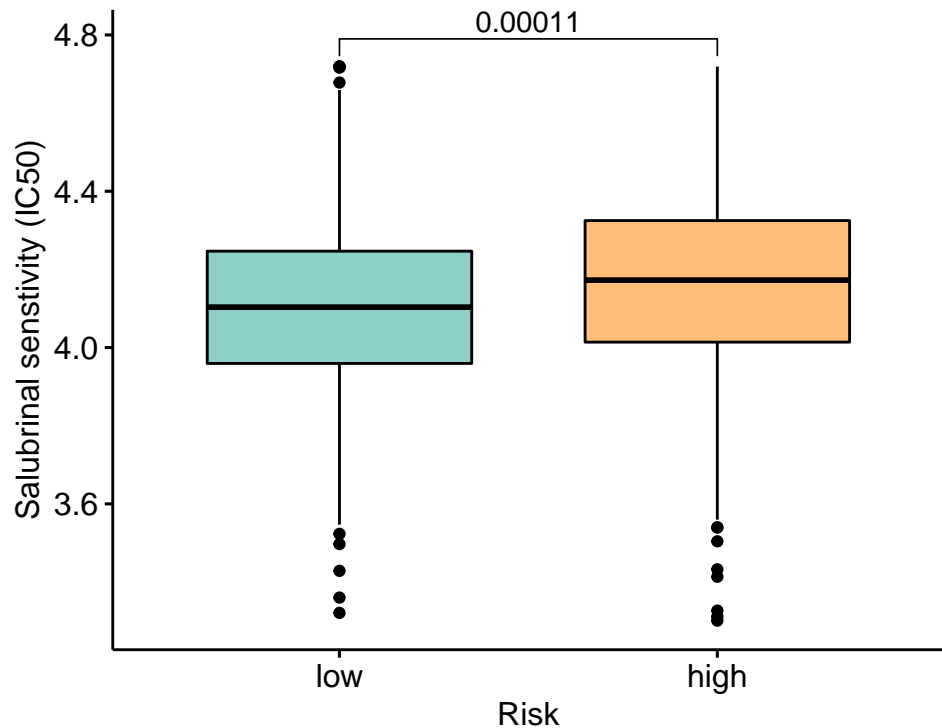

Supplement: Supplementary File 1 — 98 drugs were with significant differences in IC50 concentrations between high and low risk groups. [file DataSheet_1.zip › 1.durgSenstivity/durgSenstivity.Salubrinal.pdf]

Risk 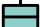 low 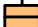 high

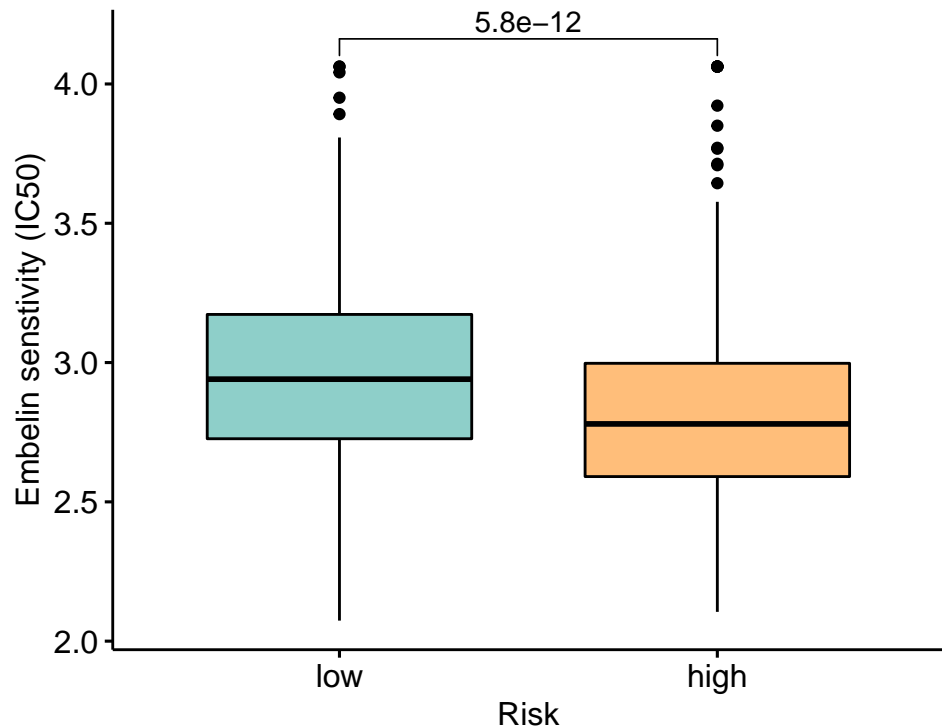

Supplement: Supplementary File 1 — 98 drugs were with significant differences in IC50 concentrations between high and low risk groups. [file DataSheet_1.zip › 1.durgSenstivity/durgSenstivity.Embelin.pdf]

Risk 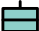 low 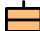 high

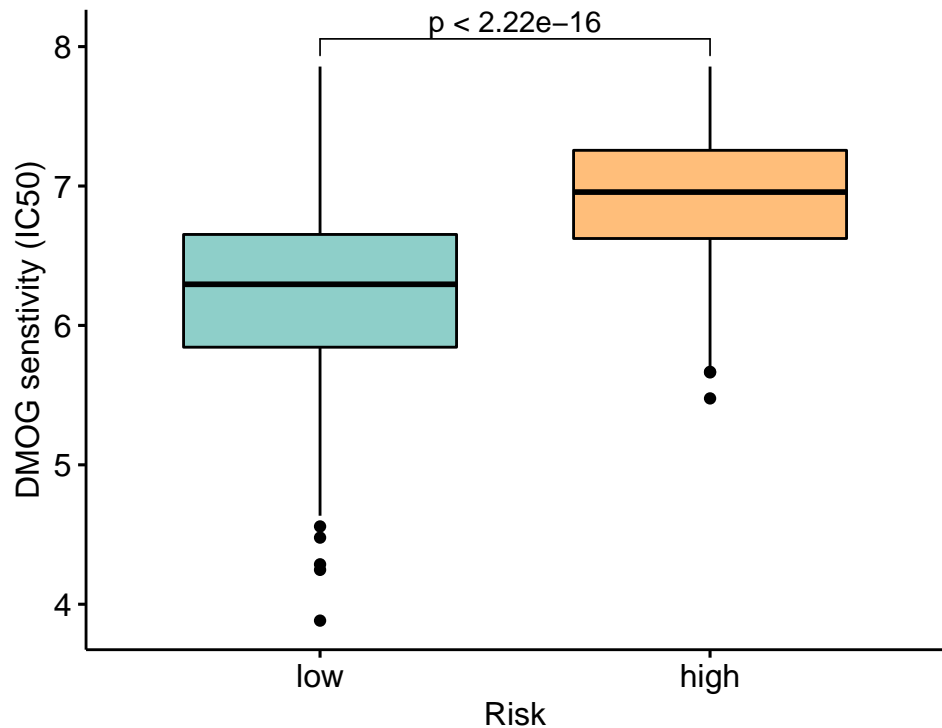

Supplement: Supplementary File 1 — 98 drugs were with significant differences in IC50 concentrations between high and low risk groups. [file DataSheet_1.zip › 1.durgSenstivity/durgSenstivity.DMOG.pdf]

Risk 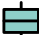 low 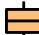 high

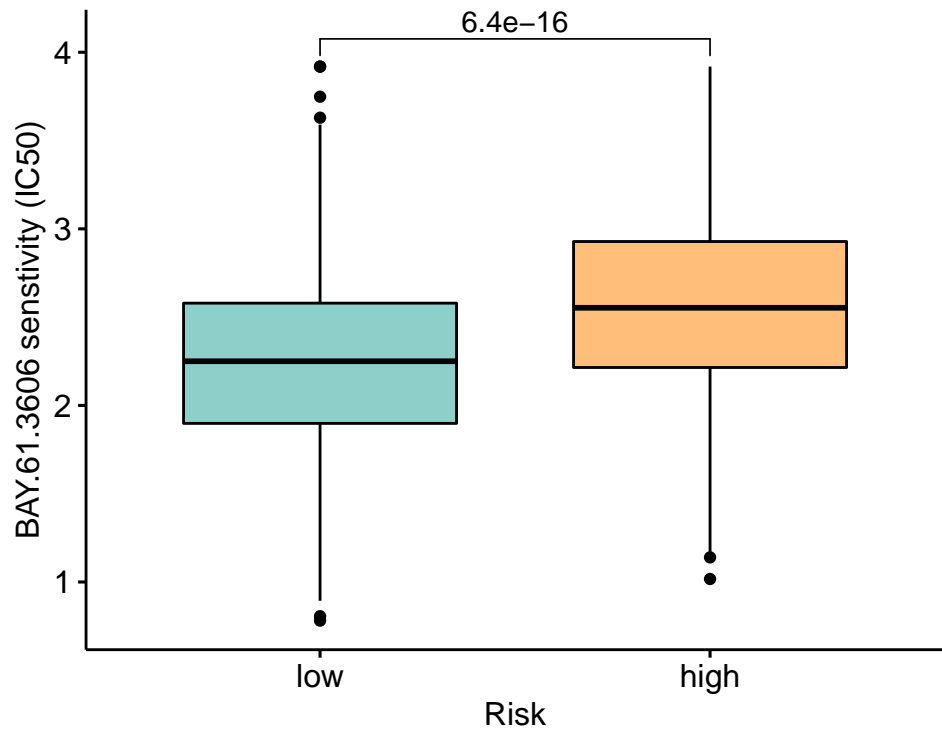

Supplement: Supplementary File 1 — 98 drugs were with significant differences in IC50 concentrations between high and low risk groups. [file DataSheet_1.zip › 1.durgSenstivity/durgSenstivity.BAY.61.3606.pdf]

Risk 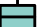 low 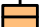 high

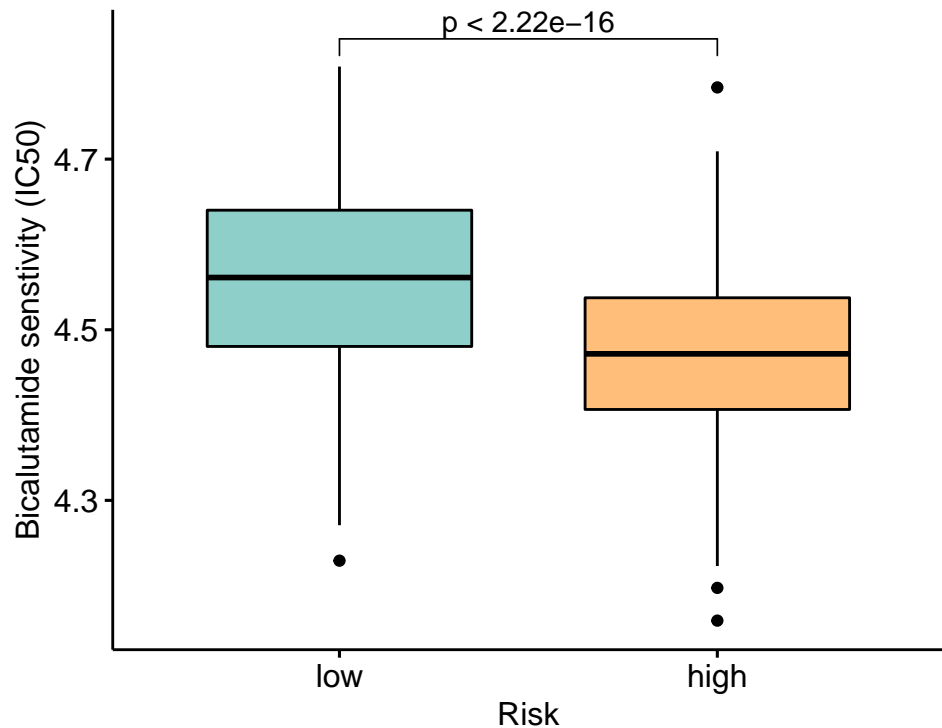

Supplement: Supplementary File 1 — 98 drugs were with significant differences in IC50 concentrations between high and low risk groups. [file DataSheet_1.zip › 1.durgSenstivity/durgSenstivity.Bicalutamide.pdf]

Risk 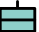 low 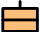 high

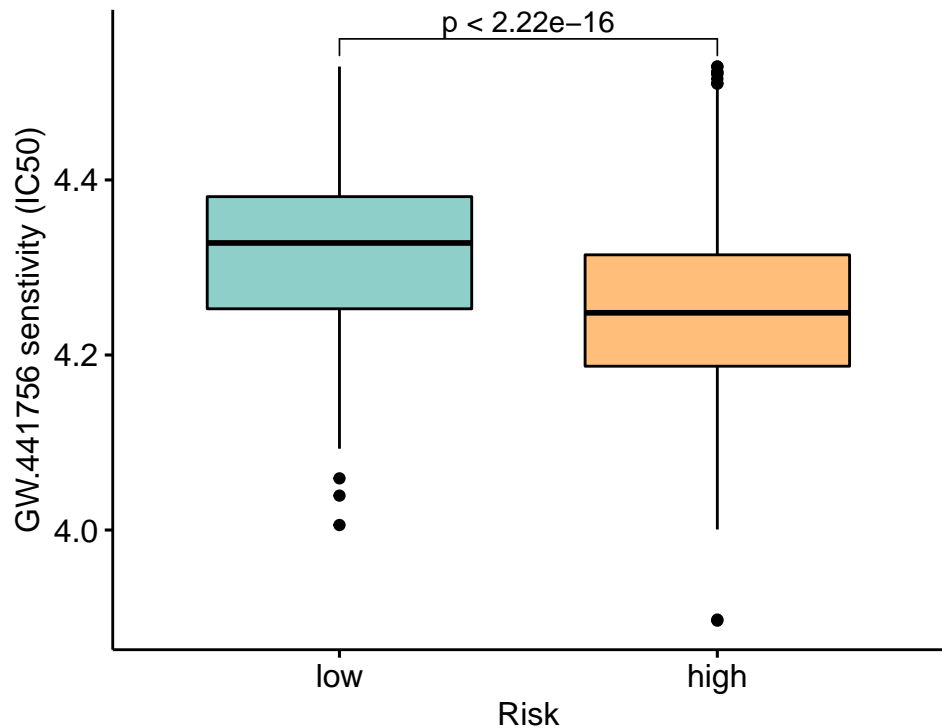

Supplement: Supplementary File 1 — 98 drugs were with significant differences in IC50 concentrations between high and low risk groups. [file DataSheet_1.zip › 1.durgSenstivity/durgSenstivity.GW.441756.pdf]

Risk 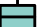 low 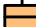 high

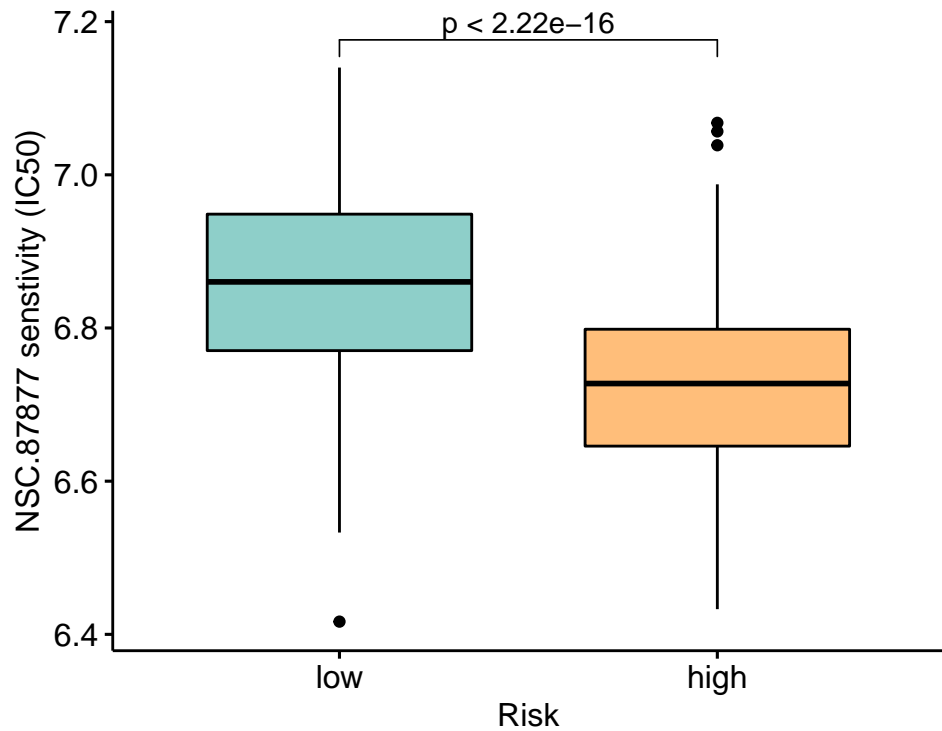

Supplement: Supplementary File 1 — 98 drugs were with significant differences in IC50 concentrations between high and low risk groups. [file DataSheet_1.zip › 1.durgSenstivity/durgSenstivity.NSC.87877.pdf]

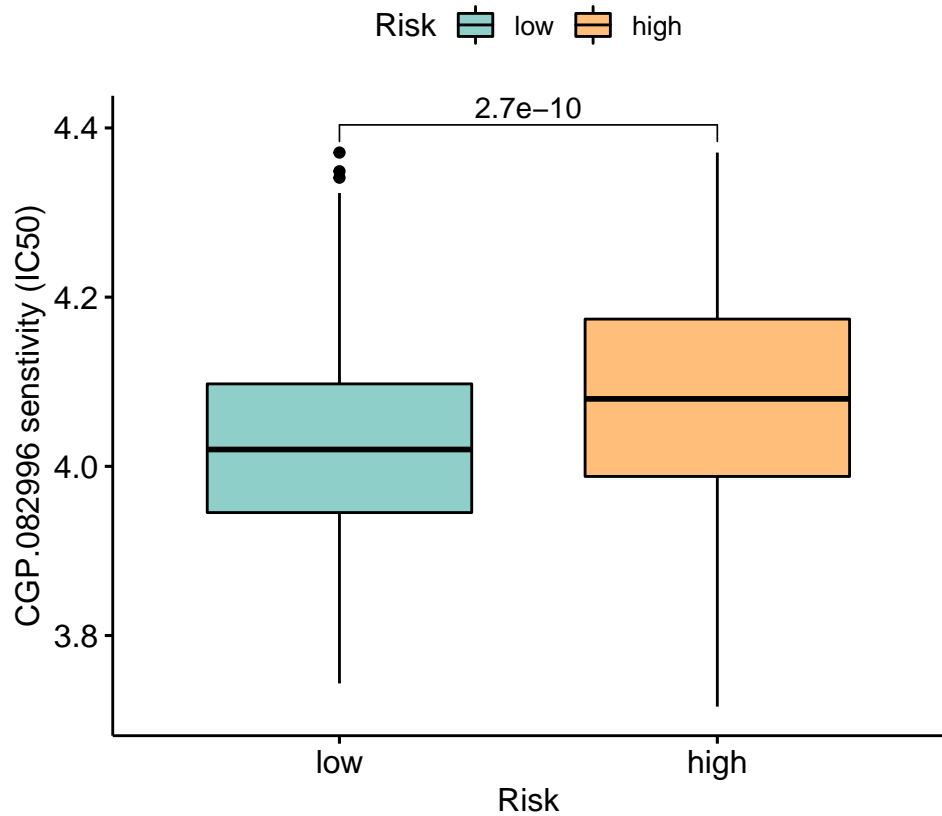

Supplement: Supplementary File 1 — 98 drugs were with significant differences in IC50 concentrations between high and low risk groups. [file DataSheet_1.zip › 1.durgSenstivity/durgSenstivity.CGP.082996.pdf]

Risk 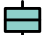 low 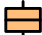 high

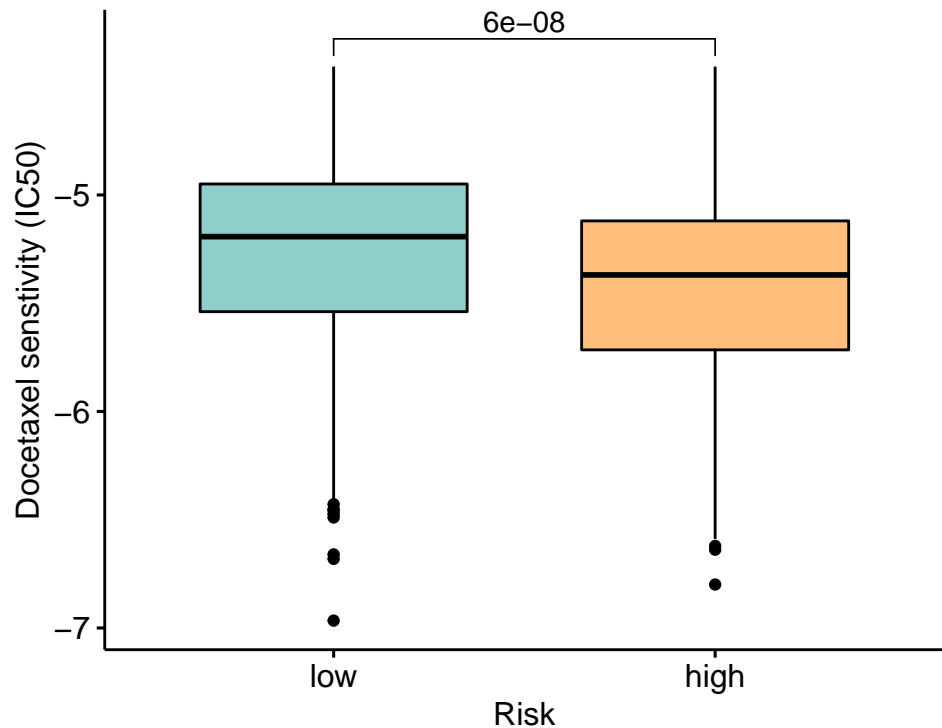

Supplement: Supplementary File 1 — 98 drugs were with significant differences in IC50 concentrations between high and low risk groups. [file DataSheet_1.zip › 1.durgSenstivity/durgSenstivity.Docetaxel.pdf]

Risk 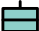 low 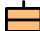 high

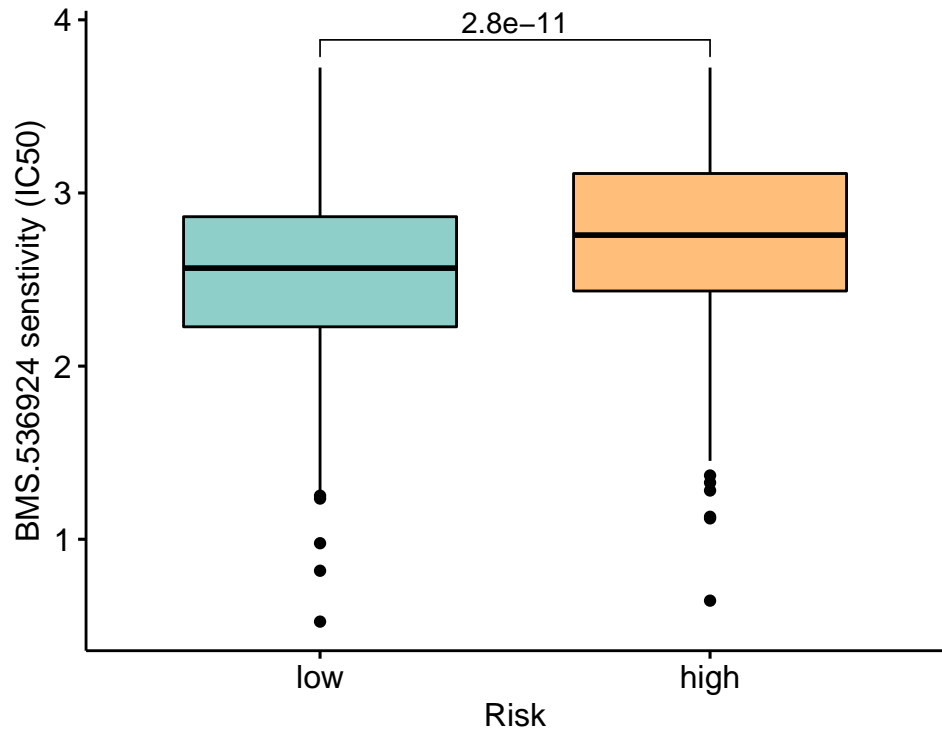

Supplement: Supplementary File 1 — 98 drugs were with significant differences in IC50 concentrations between high and low risk groups. [file DataSheet_1.zip › 1.durgSenstivity/durgSenstivity.BMS.536924.pdf]

Risk 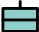 low 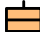 high

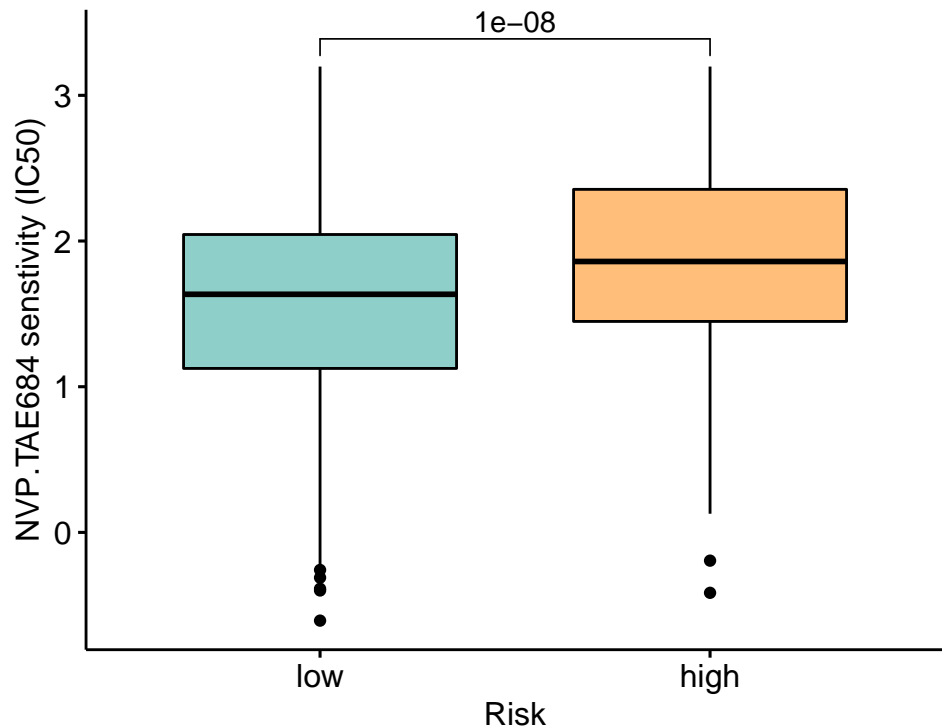

Supplement: Supplementary File 1 — 98 drugs were with significant differences in IC50 concentrations between high and low risk groups. [file DataSheet_1.zip › 1.durgSenstivity/durgSenstivity.NVP.TAE684.pdf]

Risk 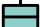 low 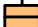 high

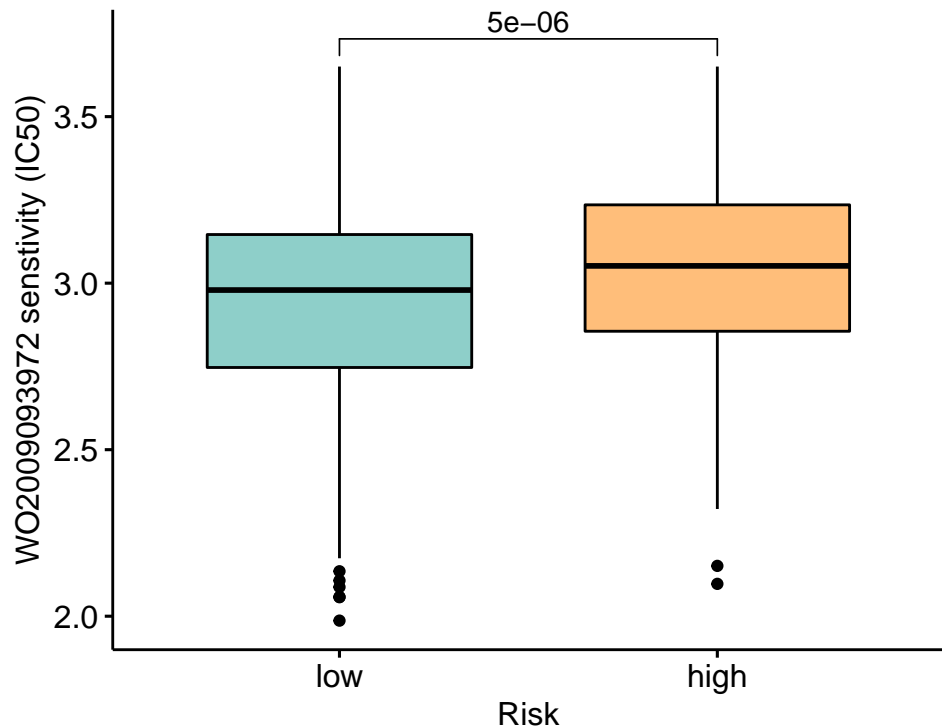

Supplement: Supplementary File 1 — 98 drugs were with significant differences in IC50 concentrations between high and low risk groups. [file DataSheet_1.zip › 1.durgSenstivity/durgSenstivity.WO2009093972.pdf]

Risk 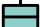 low 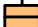 high

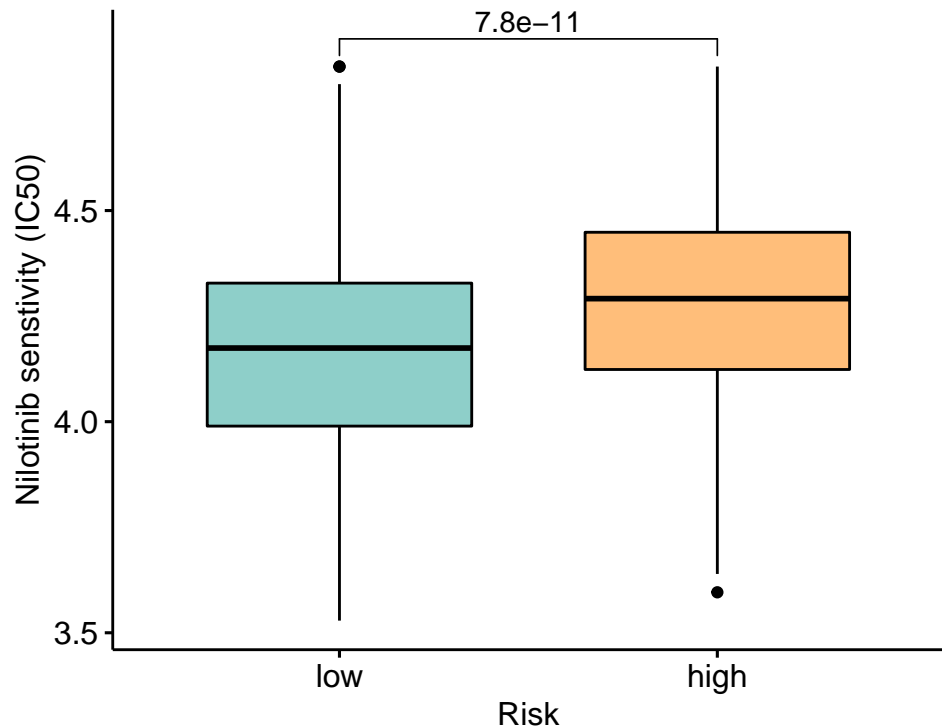

Supplement: Supplementary File 1 — 98 drugs were with significant differences in IC50 concentrations between high and low risk groups. [file DataSheet_1.zip › 1.durgSenstivity/durgSenstivity.Nilotinib.pdf]

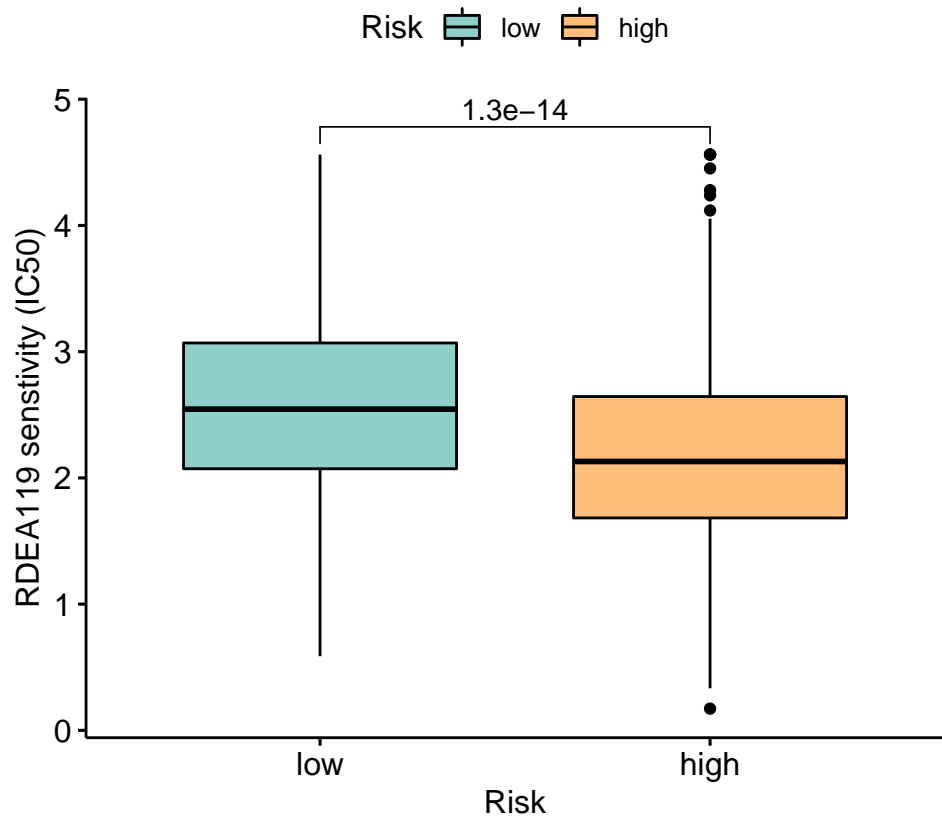

Supplement: Supplementary File 1 — 98 drugs were with significant differences in IC50 concentrations between high and low risk groups. [file DataSheet_1.zip › 1.durgSenstivity/durgSenstivity.RDEA119.pdf]

Risk 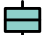 low 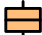 high

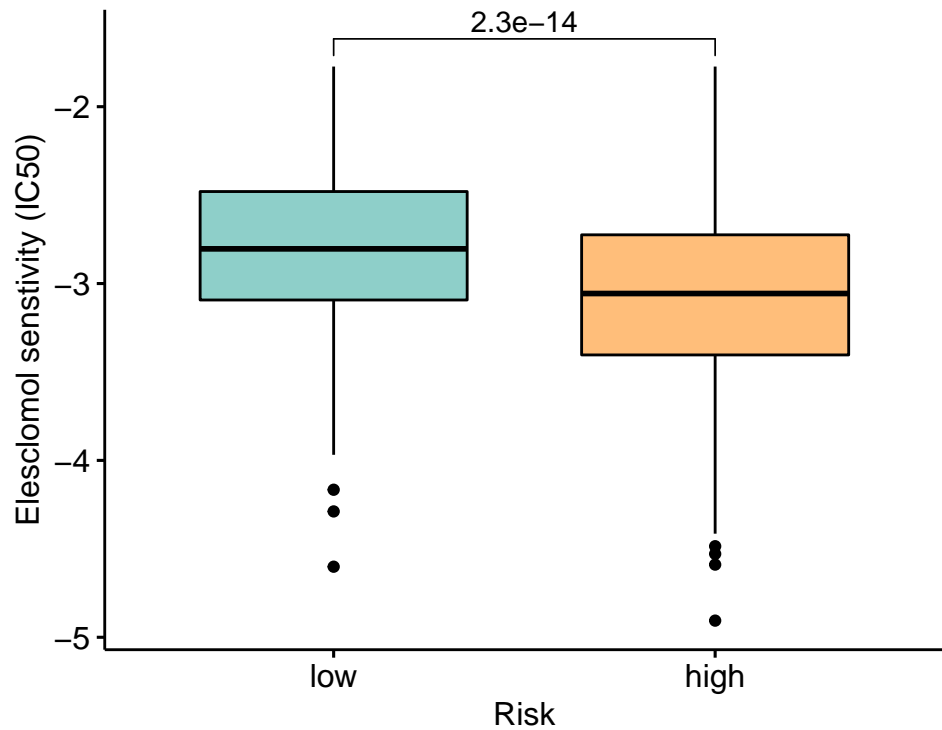

Supplement: Supplementary File 1 — 98 drugs were with significant differences in IC50 concentrations between high and low risk groups. [file DataSheet_1.zip › 1.durgSenstivity/durgSenstivity.Elesclomol.pdf]

Risk 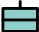 low 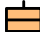 high

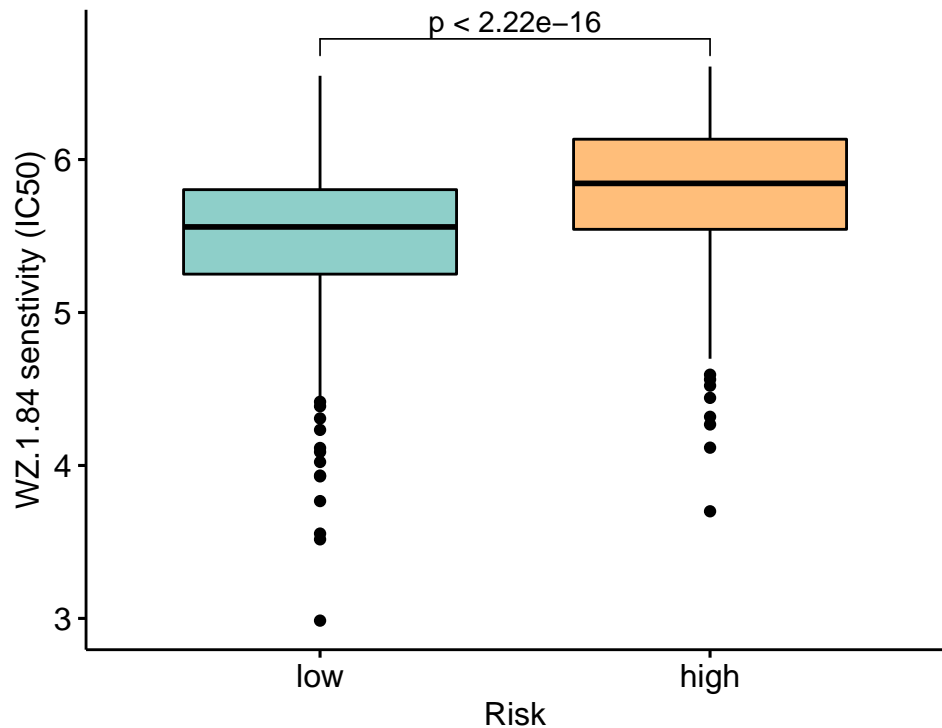

Supplement: Supplementary File 1 — 98 drugs were with significant differences in IC50 concentrations between high and low risk groups. [file DataSheet_1.zip › 1.durgSenstivity/durgSenstivity.WZ.1.84.pdf]

Risk 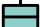 low 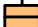 high

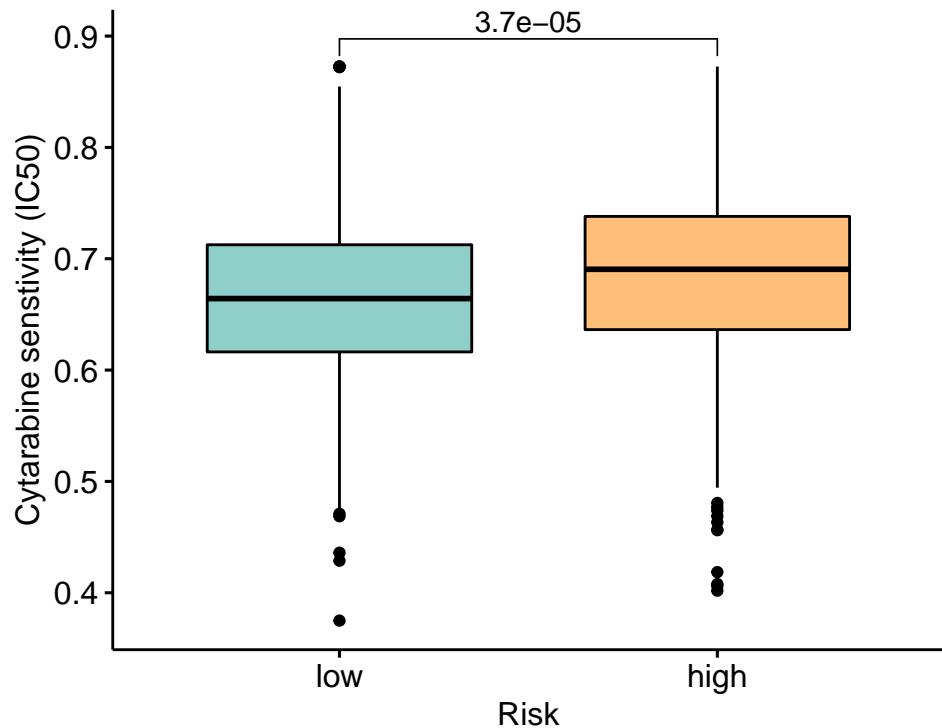

Supplement: Supplementary File 1 — 98 drugs were with significant differences in IC50 concentrations between high and low risk groups. [file DataSheet_1.zip › 1.durgSenstivity/durgSenstivity.Cytarabine.pdf]

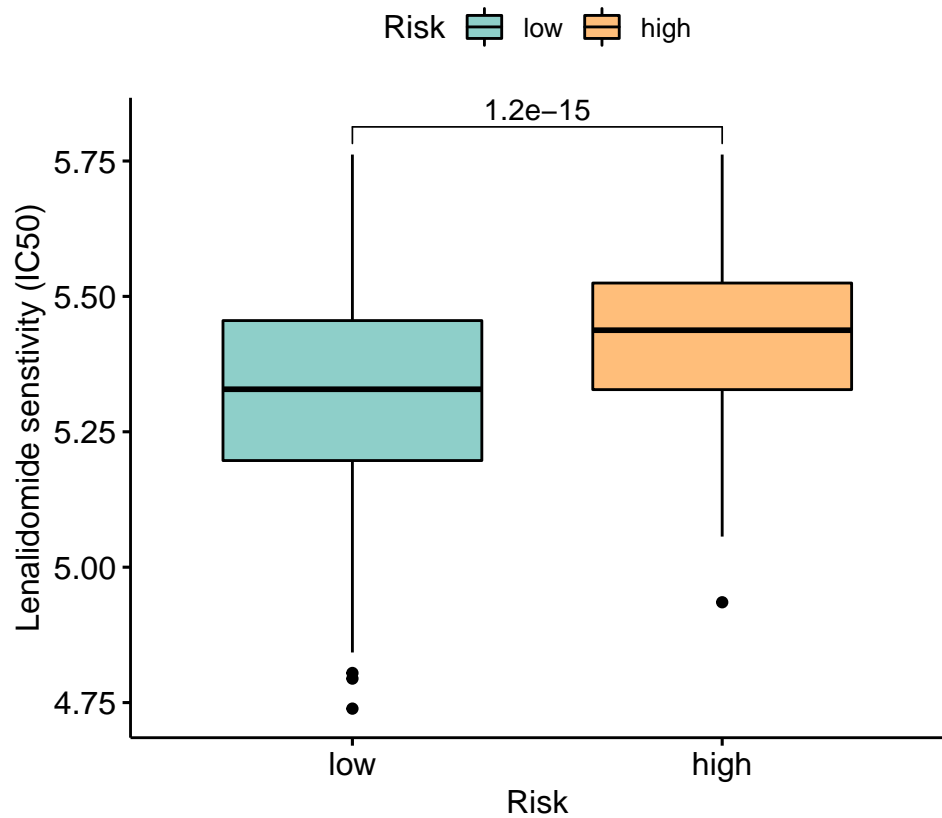

Supplement: Supplementary File 1 — 98 drugs were with significant differences in IC50 concentrations between high and low risk groups. [file DataSheet_1.zip › 1.durgSenstivity/durgSenstivity.Lenalidomide.pdf]

Risk 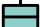 low 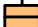 high

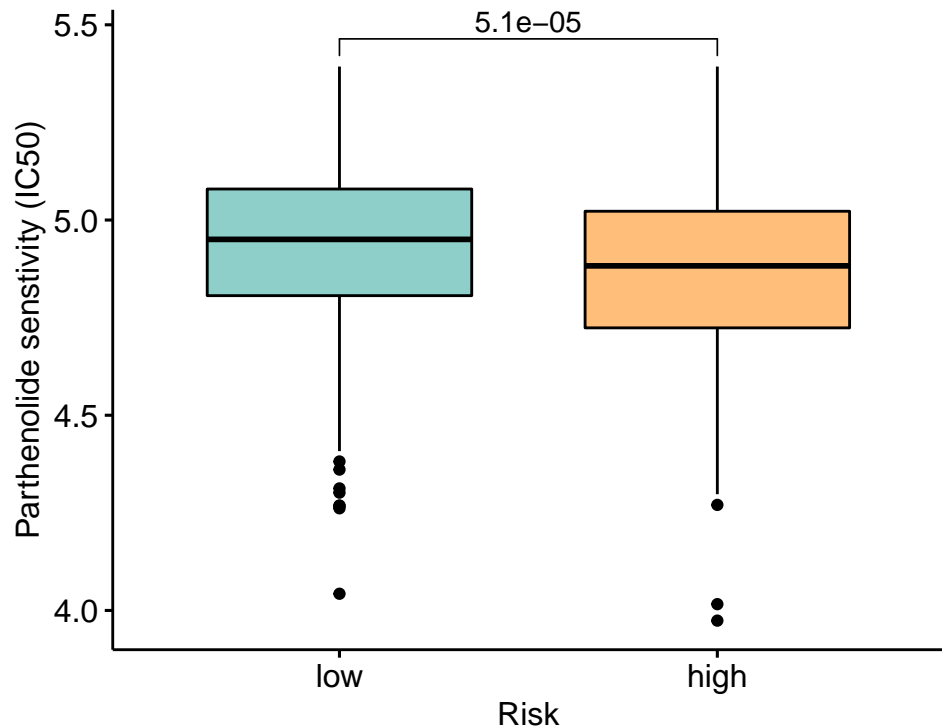

Supplement: Supplementary File 1 — 98 drugs were with significant differences in IC50 concentrations between high and low risk groups. [file DataSheet_1.zip › 1.durgSenstivity/durgSenstivity.Parthenolide.pdf]

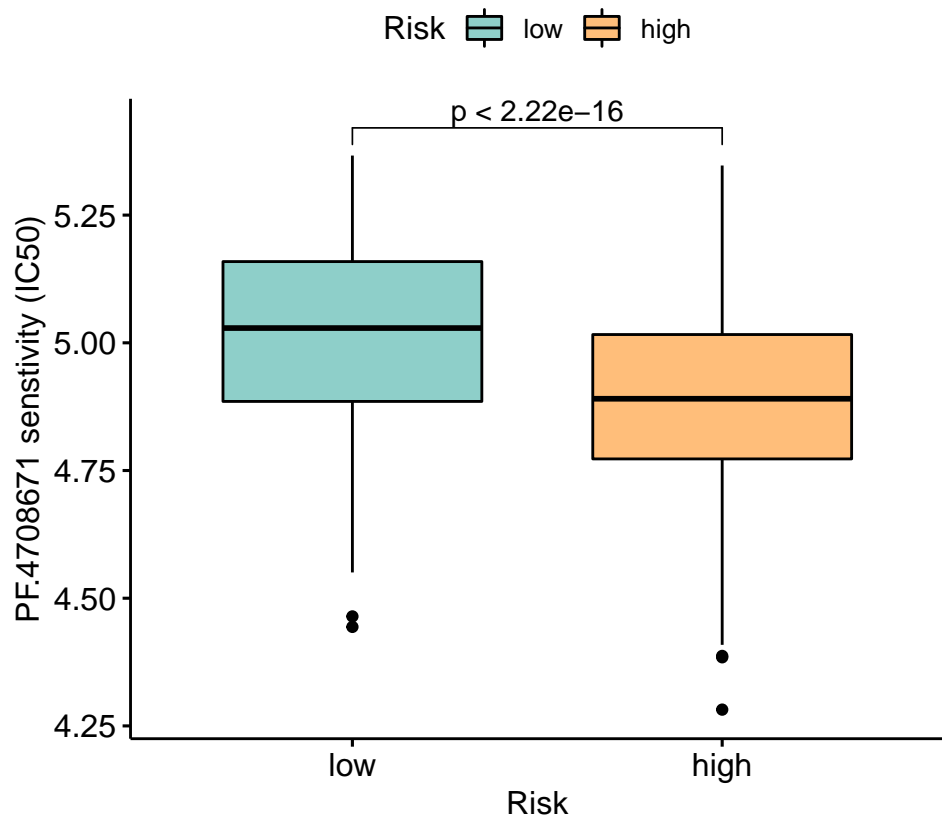

Supplement: Supplementary File 1 — 98 drugs were with significant differences in IC50 concentrations between high and low risk groups. [file DataSheet_1.zip › 1.durgSenstivity/durgSenstivity.PF.4708671.pdf]

Risk 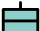 low 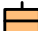 high

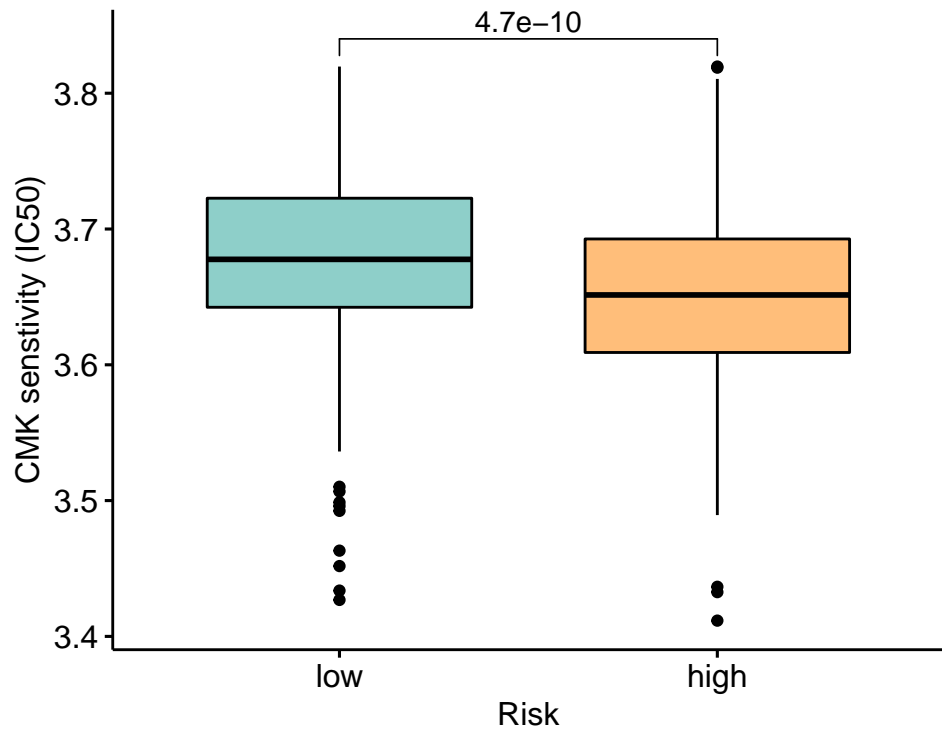

Supplement: Supplementary File 1 — 98 drugs were with significant differences in IC50 concentrations between high and low risk groups. [file DataSheet_1.zip › 1.durgSenstivity/durgSenstivity.CMK.pdf]

Risk 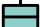 low 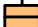 high

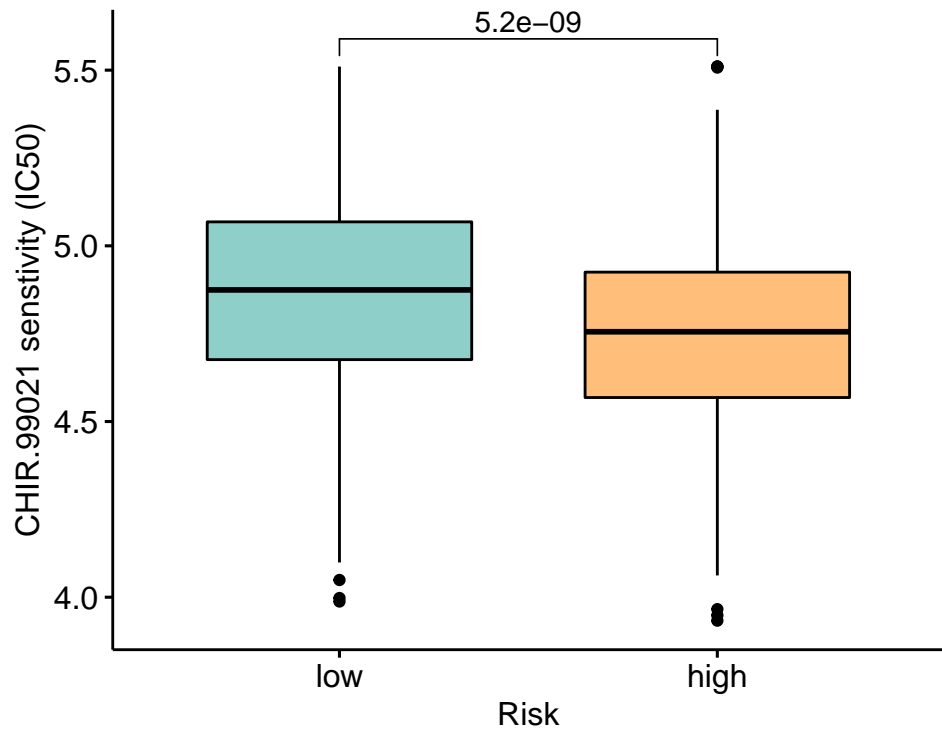

Supplement: Supplementary File 1 — 98 drugs were with significant differences in IC50 concentrations between high and low risk groups. [file DataSheet_1.zip › 1.durgSenstivity/durgSenstivity.CHIR.99021.pdf]

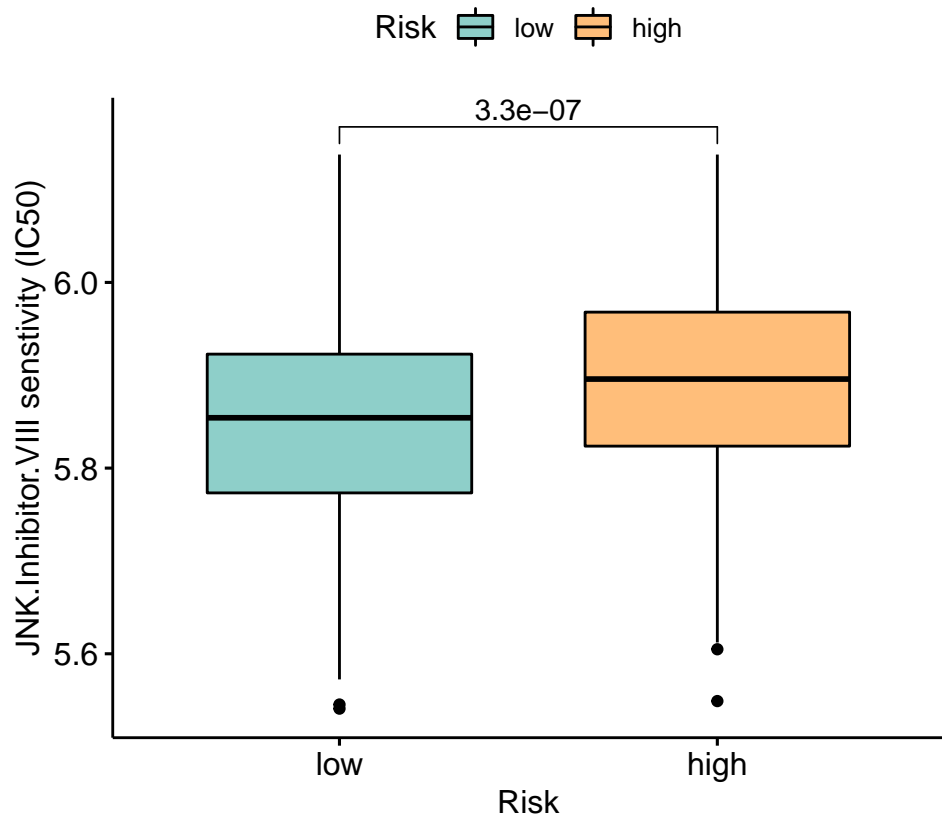

Supplement: Supplementary File 1 — 98 drugs were with significant differences in IC50 concentrations between high and low risk groups. [file DataSheet_1.zip › 1.durgSenstivity/durgSenstivity.JNK.Inhibitor.VIII.pdf]

JNK.9L sensitivity (IC50)

Risk 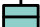 low 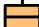 high

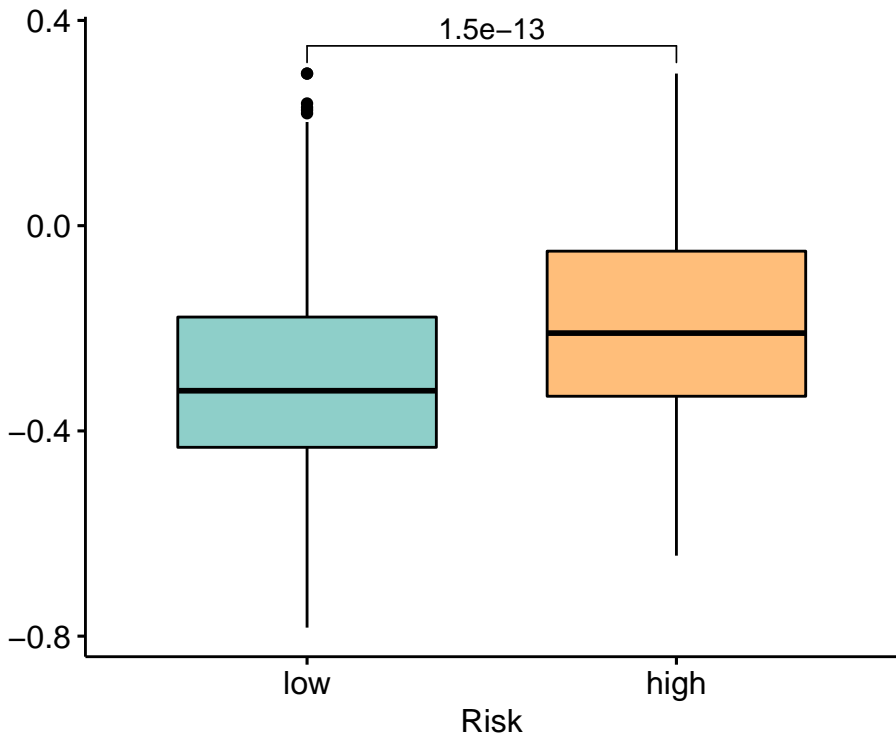

Supplement: Supplementary File 1 — 98 drugs were with significant differences in IC50 concentrations between high and low risk groups. [file DataSheet_1.zip › 1.durgSenstivity/durgSenstivity.JNK.9L.pdf]

Risk 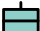 low 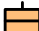 high

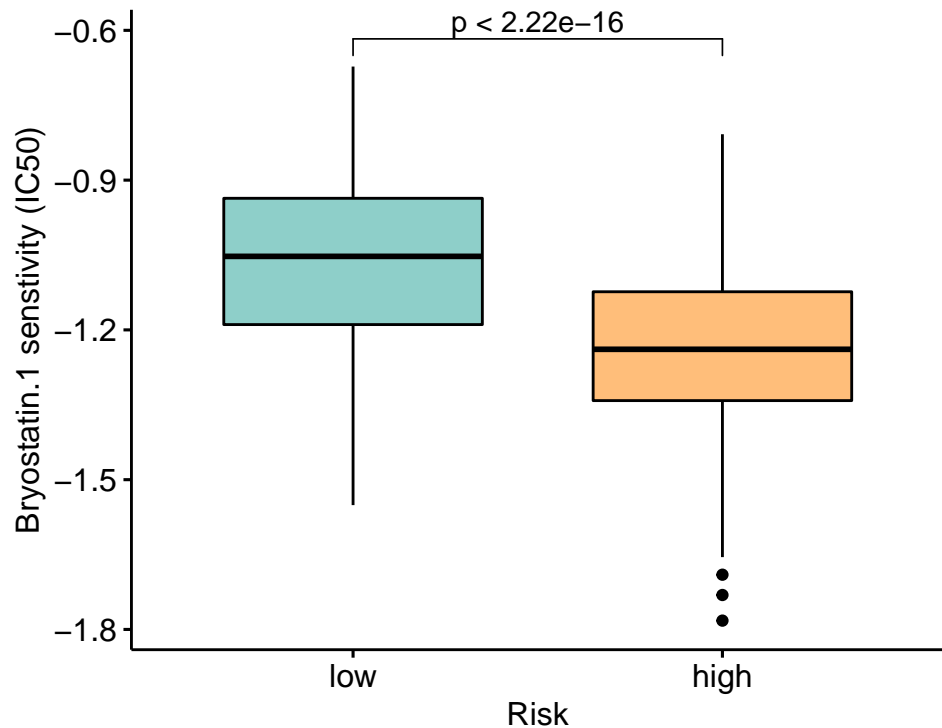

Supplement: Supplementary File 1 — 98 drugs were with significant differences in IC50 concentrations between high and low risk groups. [file DataSheet_1.zip › 1.durgSenstivity/durgSenstivity.Bryostatin.1.pdf]

Risk 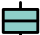 low 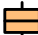 high

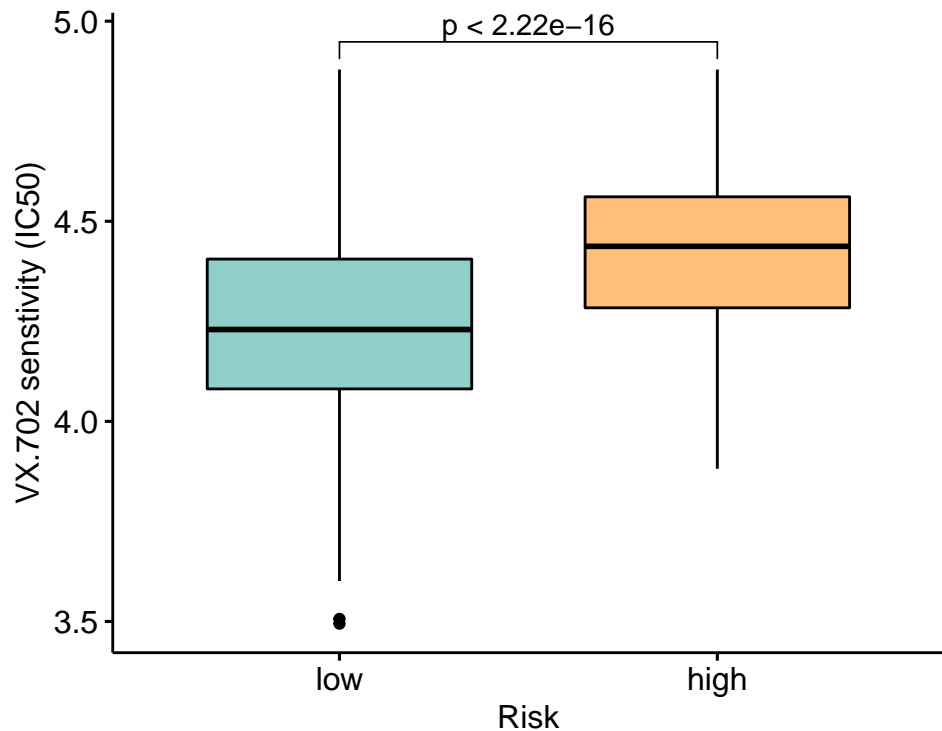

Supplement: Supplementary File 1 — 98 drugs were with significant differences in IC50 concentrations between high and low risk groups. [file DataSheet_1.zip › 1.durgSenstivity/durgSenstivity.VX.702.pdf]

Risk 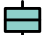 low 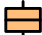 high

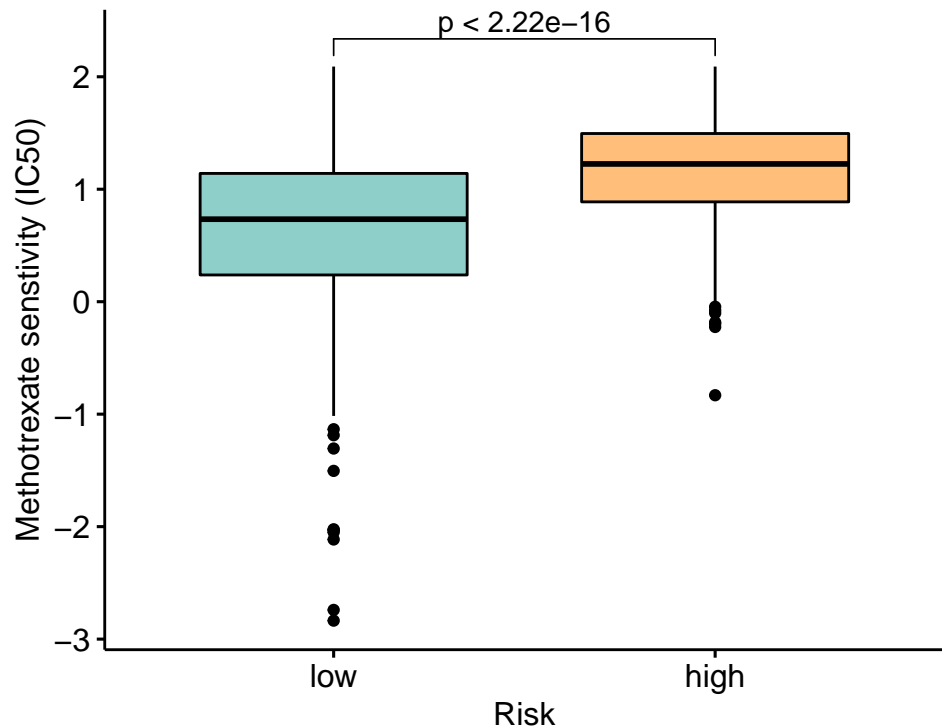

Supplement: Supplementary File 1 — 98 drugs were with significant differences in IC50 concentrations between high and low risk groups. [file DataSheet_1.zip › 1.durgSenstivity/durgSenstivity.Methotrexate.pdf]

Risk 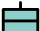 low 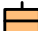 high

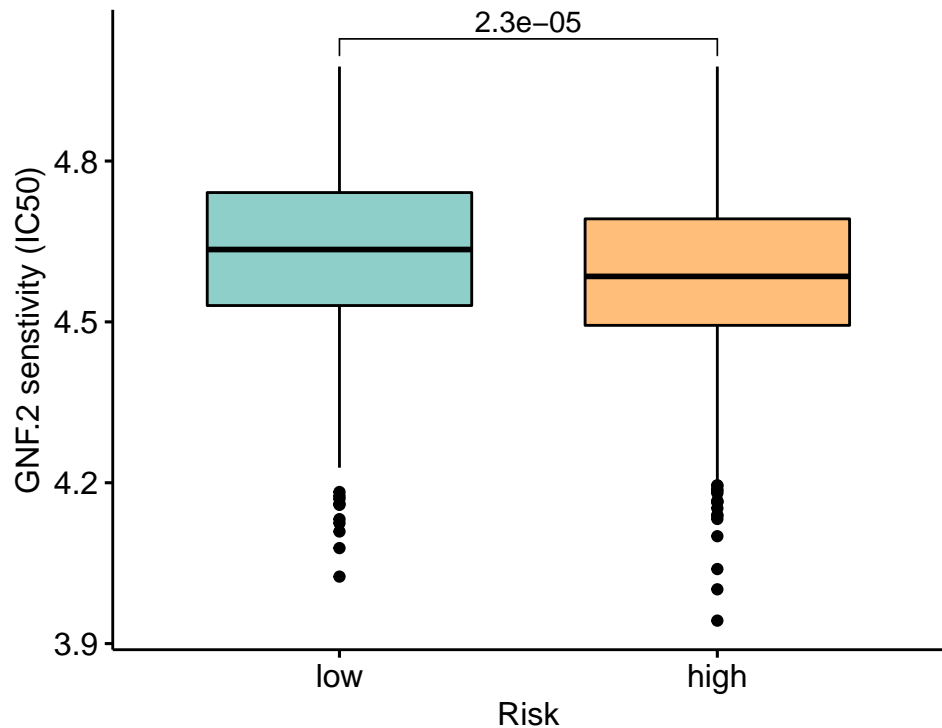

Supplement: Supplementary File 1 — 98 drugs were with significant differences in IC50 concentrations between high and low risk groups. [file DataSheet_1.zip › 1.durgSenstivity/durgSenstivity.GNF.2.pdf]

NVP.BEZ235 sensitivity (IC50)

Risk 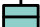 low 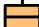 high

$6.3e-08$

low

high

Risk

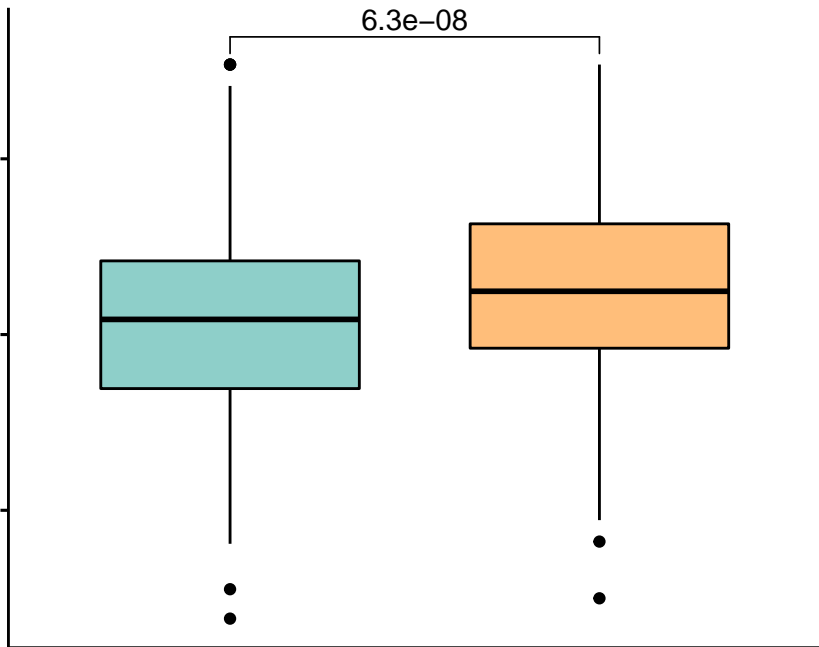

Supplement: Supplementary File 1 — 98 drugs were with significant differences in IC50 concentrations between high and low risk groups. [file DataSheet_1.zip › 1.durgSenstivity/durgSenstivity.NVP.BEZ235.pdf]

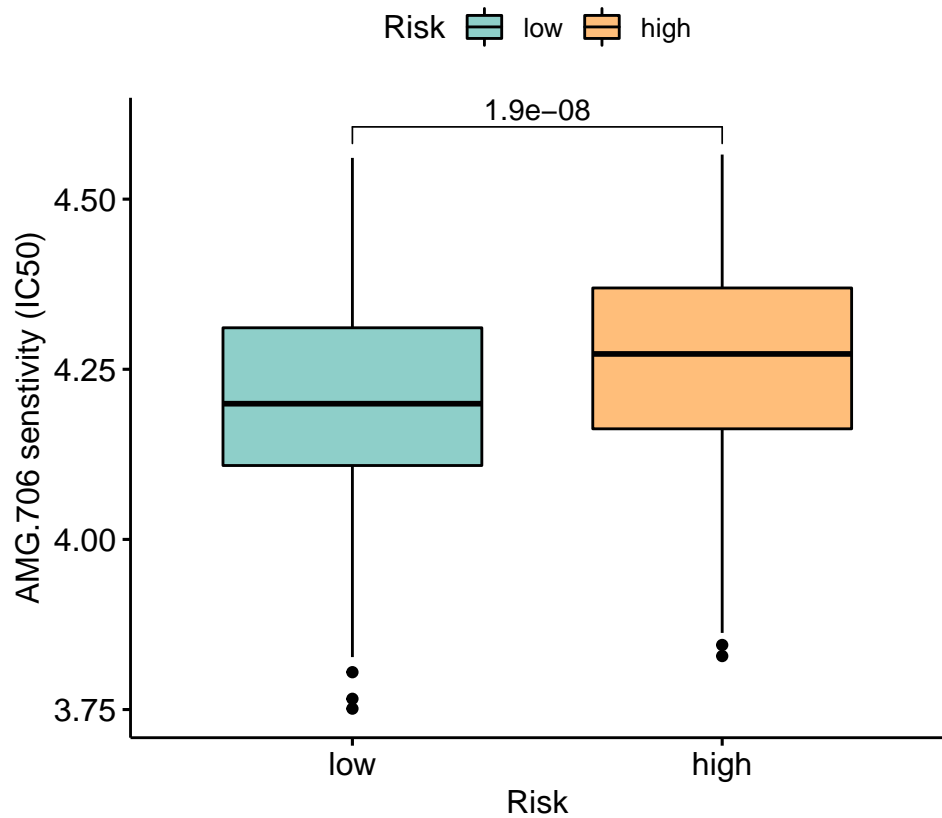

Supplement: Supplementary File 1 — 98 drugs were with significant differences in IC50 concentrations between high and low risk groups. [file DataSheet_1.zip › 1.durgSenstivity/durgSenstivity.AMG.706.pdf]

Risk 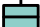 low 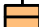 high

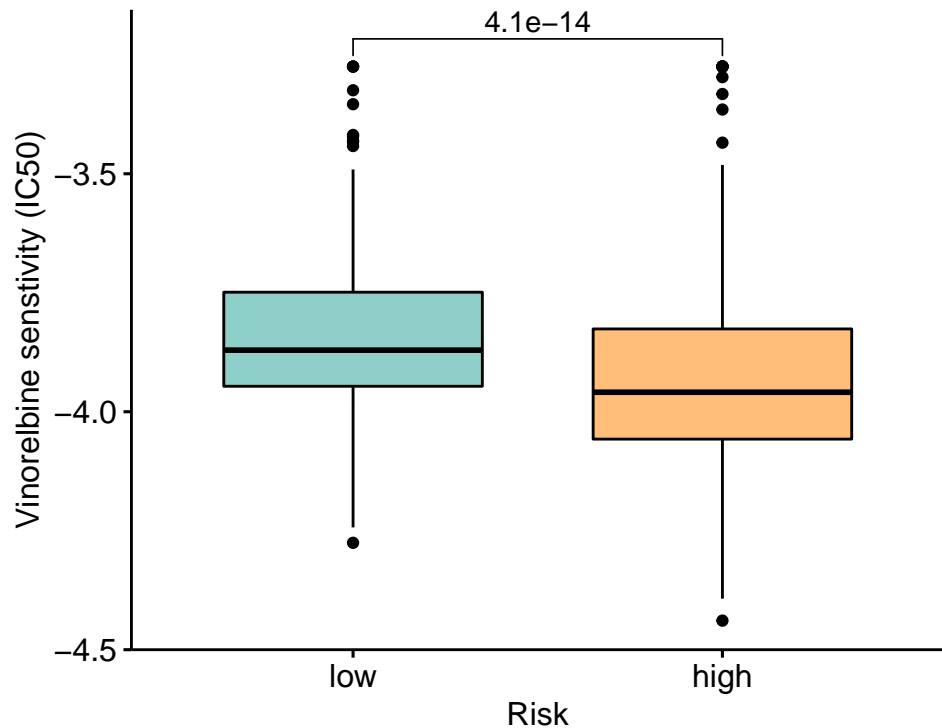

Supplement: Supplementary File 1 — 98 drugs were with significant differences in IC50 concentrations between high and low risk groups. [file DataSheet_1.zip › 1.durgSenstivity/durgSenstivity.Vinorelbine.pdf]

Risk 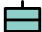 low 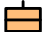 high

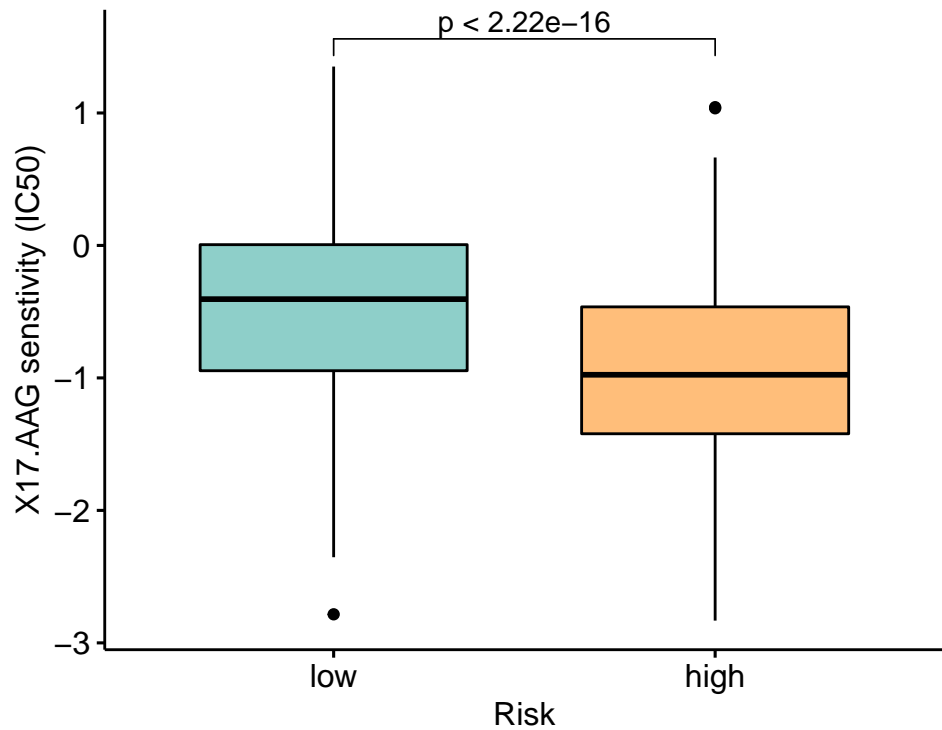

Supplement: Supplementary File 1 — 98 drugs were with significant differences in IC50 concentrations between high and low risk groups. [file DataSheet_1.zip › 1.durgSenstivity/durgSenstivity.X17.AAG.pdf]

Risk 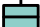 low 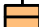 high

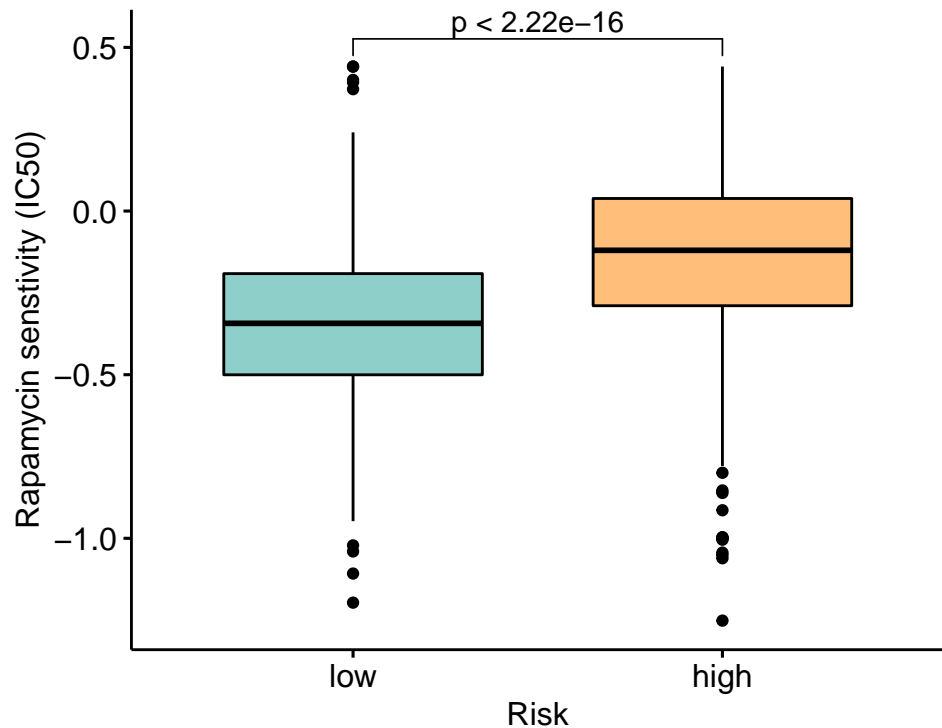

Supplement: Supplementary File 1 — 98 drugs were with significant differences in IC50 concentrations between high and low risk groups. [file DataSheet_1.zip › 1.durgSenstivity/durgSenstivity.Rapamycin.pdf]

Risk 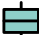 low 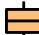 high

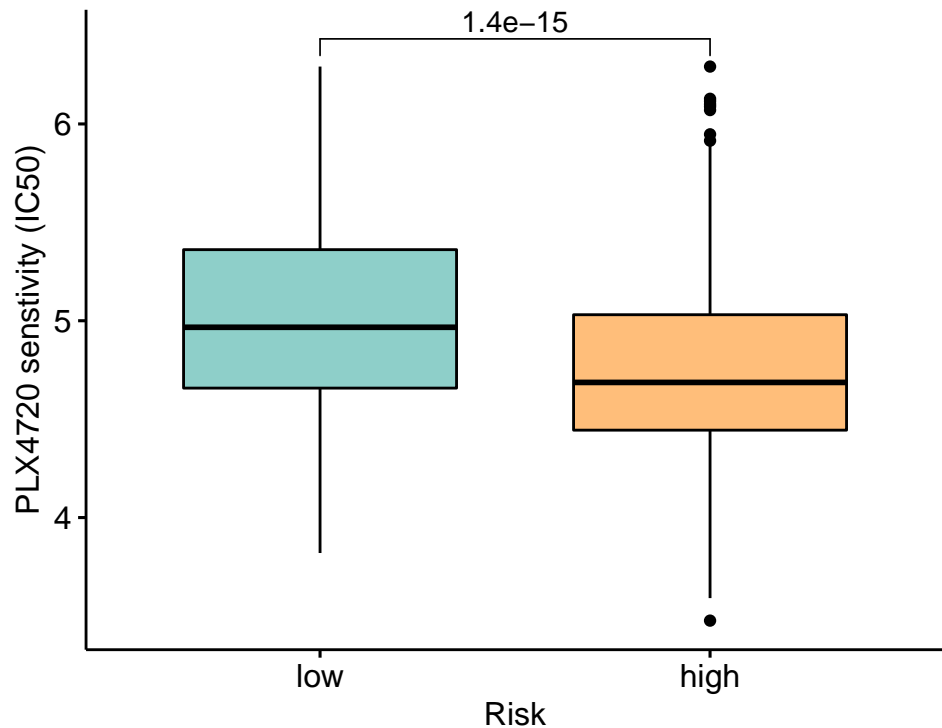

Supplement: Supplementary File 1 — 98 drugs were with significant differences in IC50 concentrations between high and low risk groups. [file DataSheet_1.zip › 1.durgSenstivity/durgSenstivity.PLX4720.pdf]

Risk 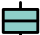 low 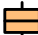 high

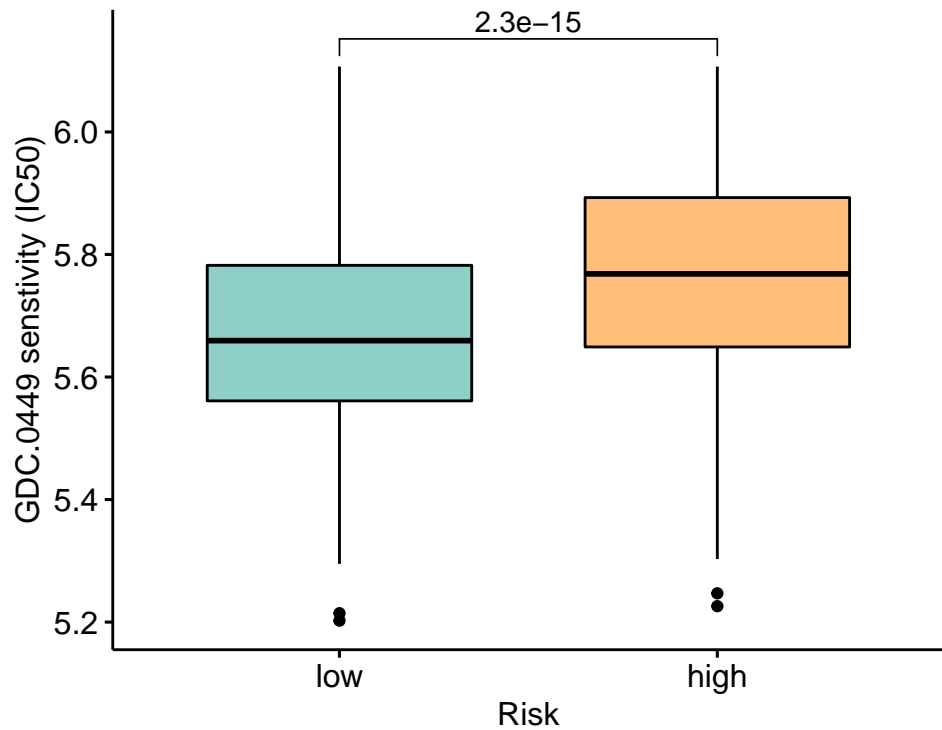

Supplement: Supplementary File 1 — 98 drugs were with significant differences in IC50 concentrations between high and low risk groups. [file DataSheet_1.zip › 1.durgSenstivity/durgSenstivity.GDC.0449.pdf]

Risk 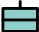 low 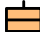 high

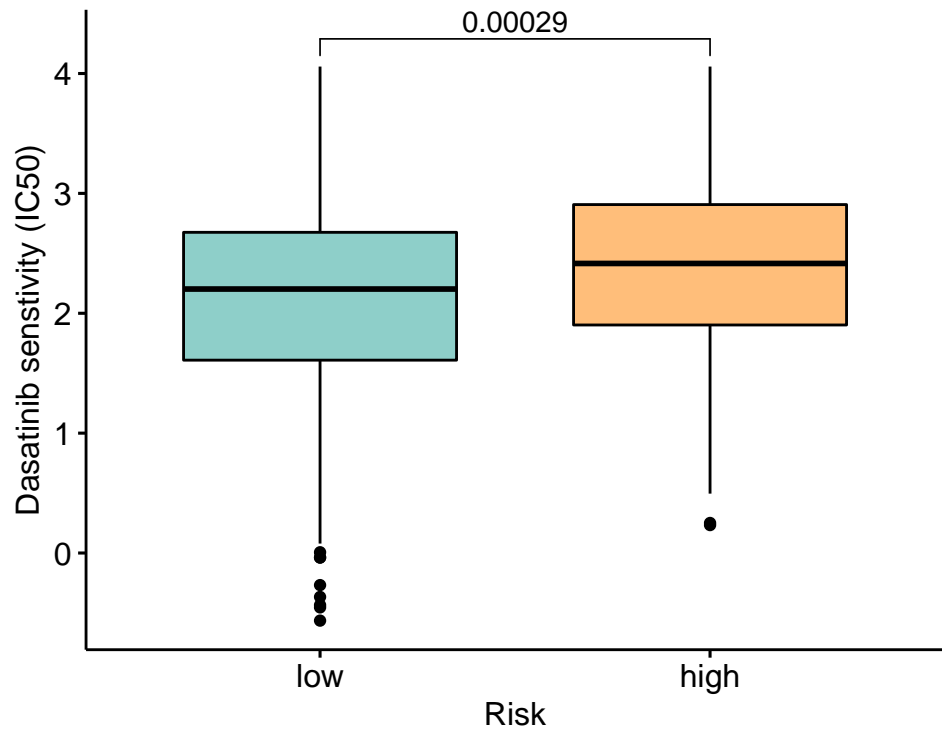

Supplement: Supplementary File 1 — 98 drugs were with significant differences in IC50 concentrations between high and low risk groups. [file DataSheet_1.zip › 1.durgSenstivity/durgSenstivity.Dasatinib.pdf]

Risk 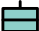 low 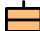 high

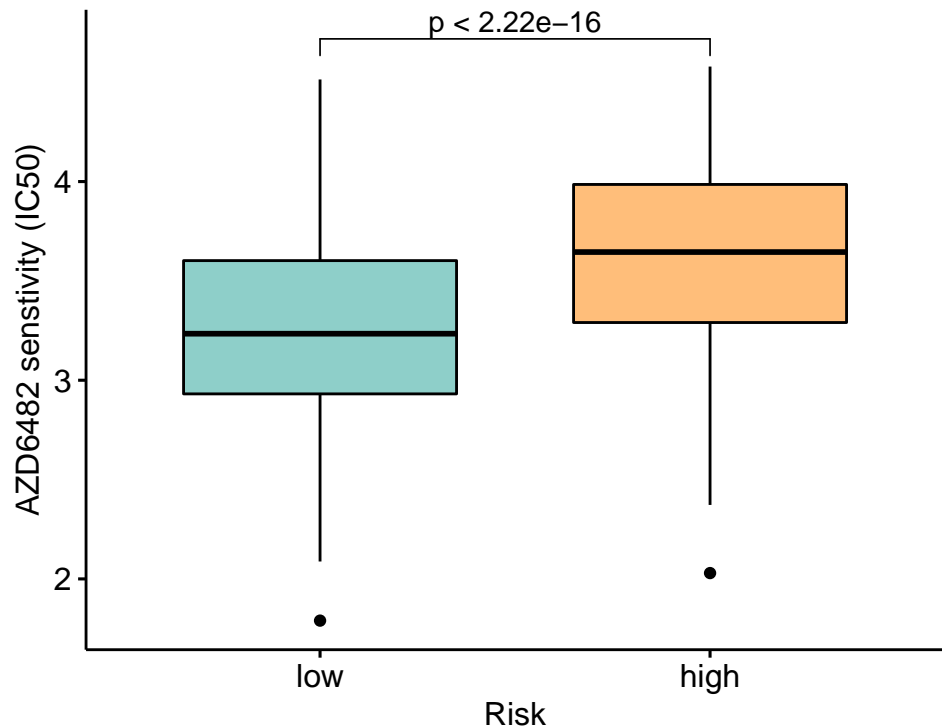

Supplement: Supplementary File 1 — 98 drugs were with significant differences in IC50 concentrations between high and low risk groups. [file DataSheet_1.zip › 1.durgSenstivity/durgSenstivity.AZD6482.pdf]

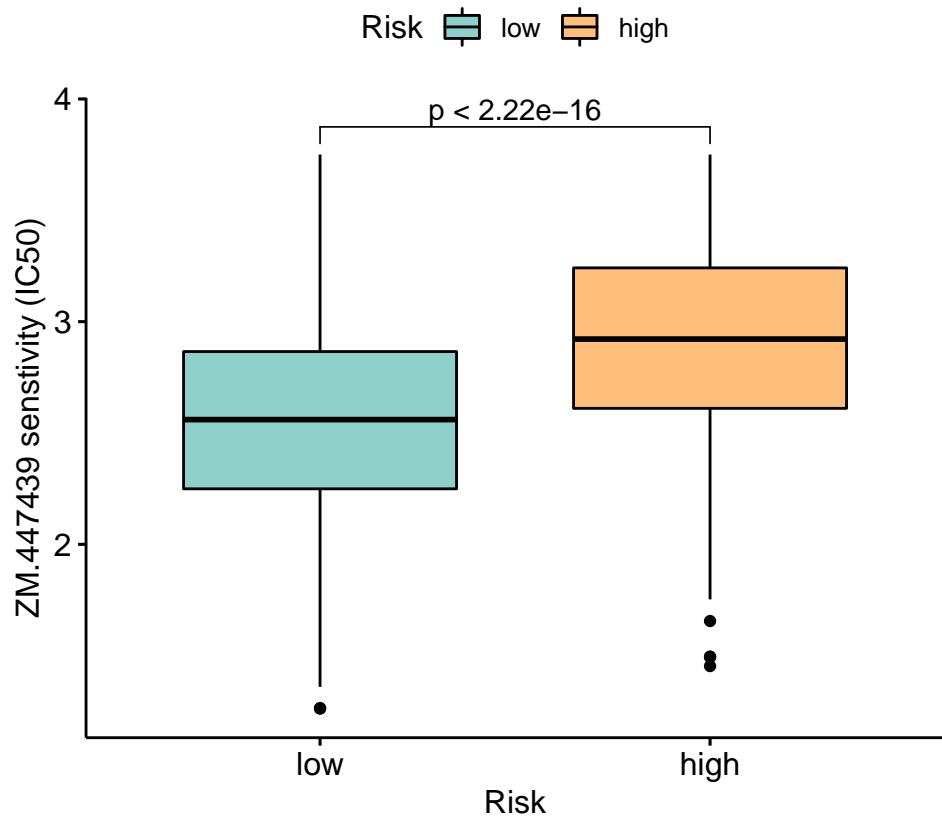

Supplement: Supplementary File 1 — 98 drugs were with significant differences in IC50 concentrations between high and low risk groups. [file DataSheet_1.zip › 1.durgSenstivity/durgSenstivity.ZM.447439.pdf]

Risk 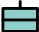 low 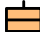 high

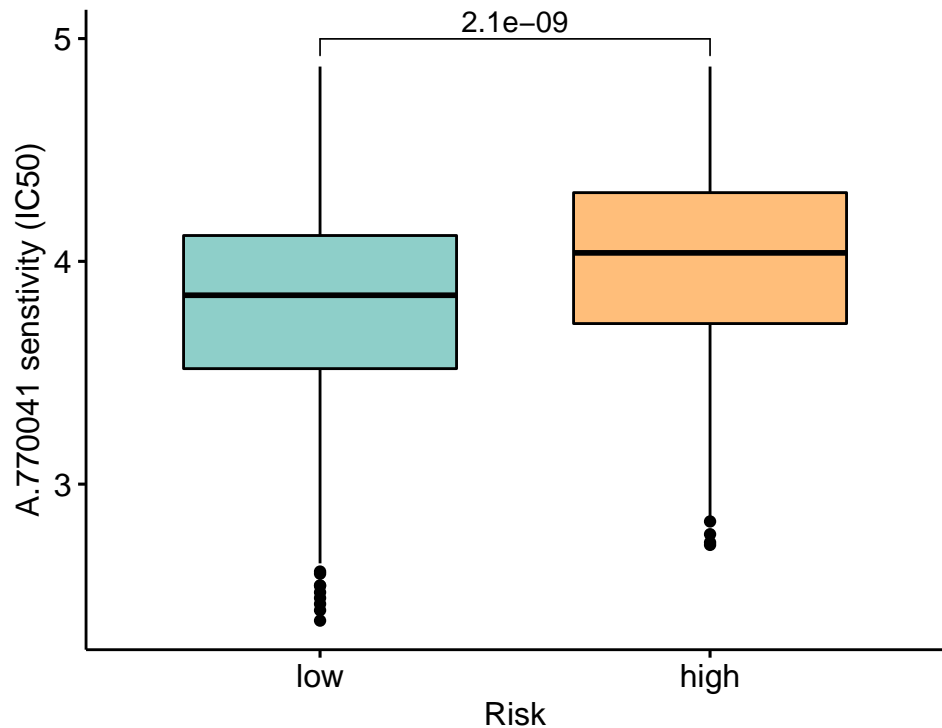

Supplement: Supplementary File 1 — 98 drugs were with significant differences in IC50 concentrations between high and low risk groups. [file DataSheet_1.zip › 1.durgSenstivity/durgSenstivity.A.770041.pdf]

Risk 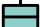 low 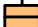 high

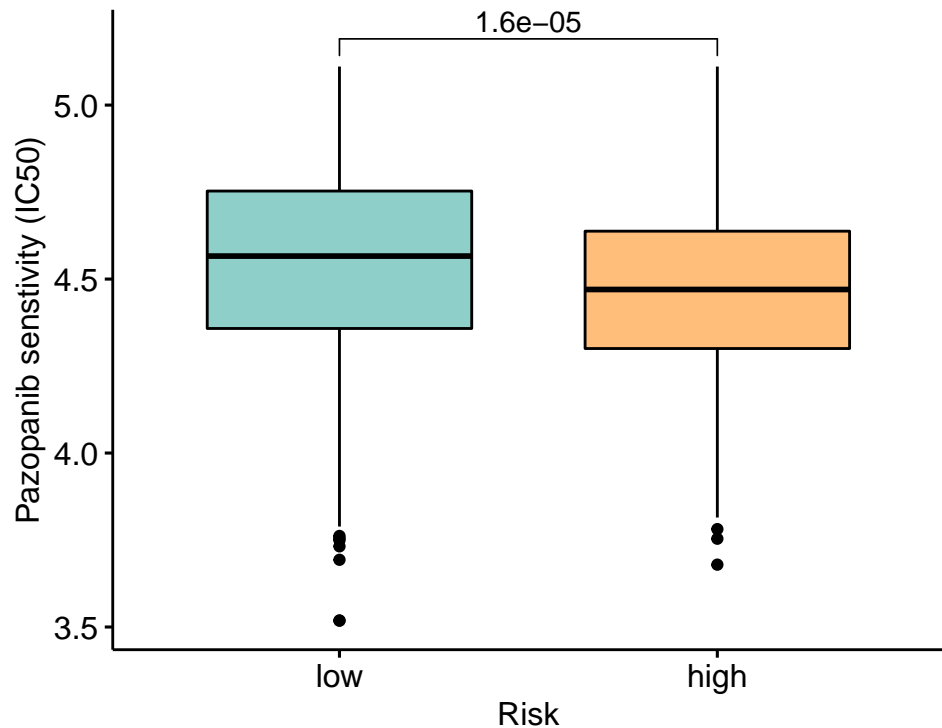

Supplement: Supplementary File 1 — 98 drugs were with significant differences in IC50 concentrations between high and low risk groups. [file DataSheet_1.zip › 1.durgSenstivity/durgSenstivity.Pazopanib.pdf]

Risk 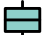 low 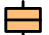 high

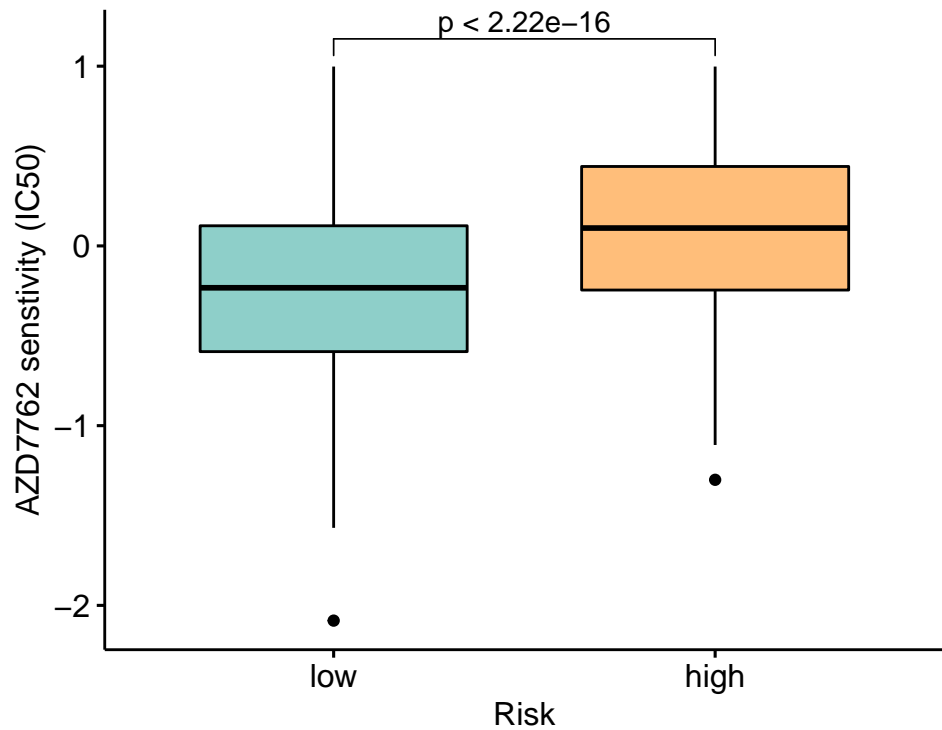

Supplement: Supplementary File 1 — 98 drugs were with significant differences in IC50 concentrations between high and low risk groups. [file DataSheet_1.zip › 1.durgSenstivity/durgSenstivity.AZD7762.pdf]

Risk 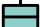 low 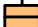 high

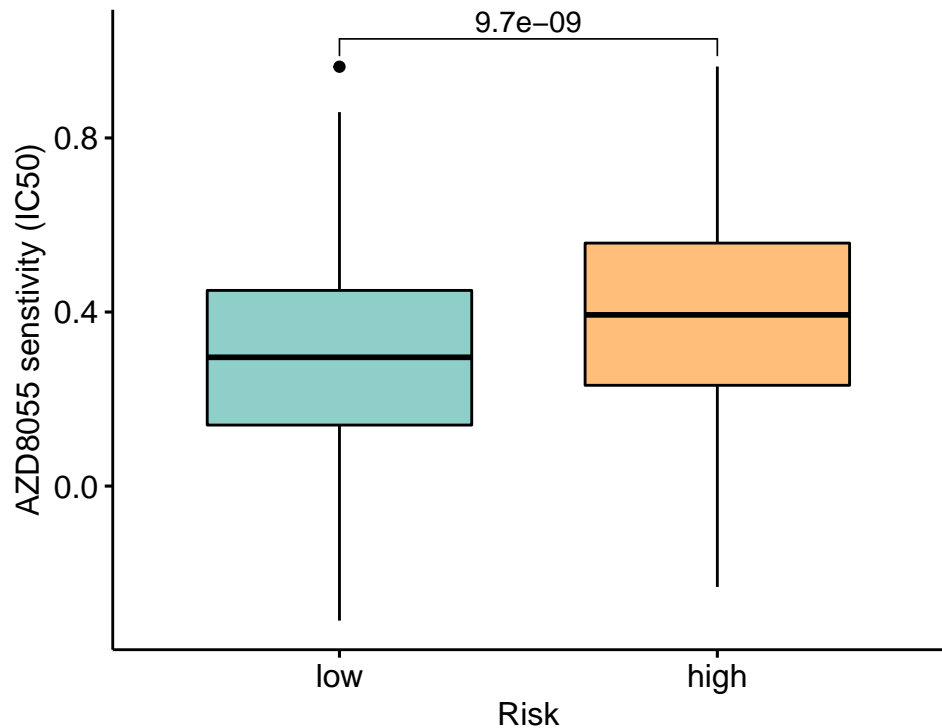

Supplement: Supplementary File 1 — 98 drugs were with significant differences in IC50 concentrations between high and low risk groups. [file DataSheet_1.zip › 1.durgSenstivity/durgSenstivity.AZD8055.pdf]

Risk 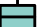 low 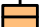 high

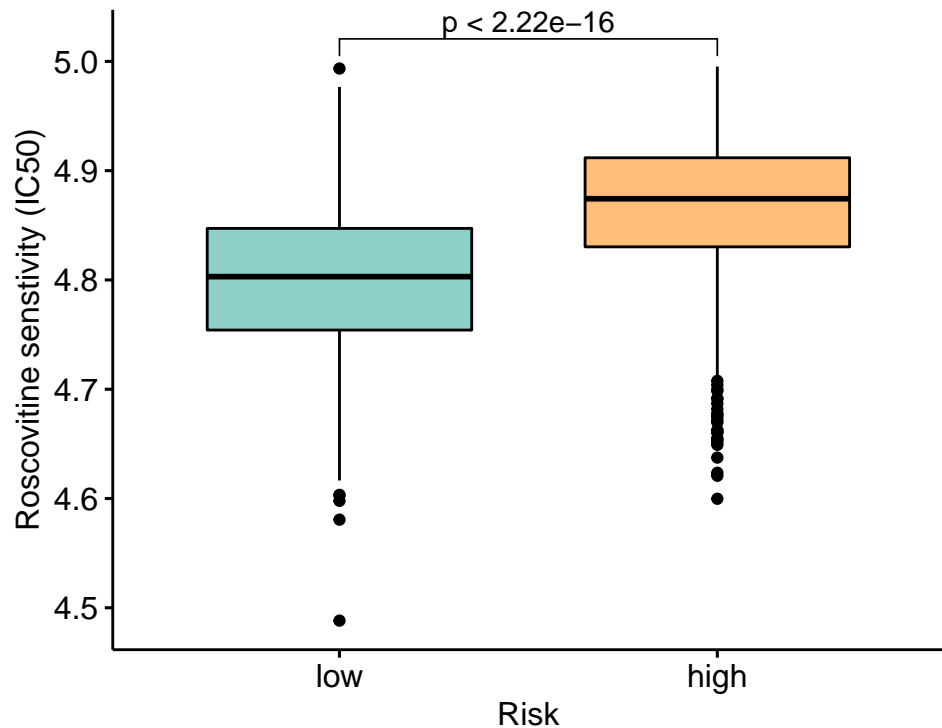

Supplement: Supplementary File 1 — 98 drugs were with significant differences in IC50 concentrations between high and low risk groups. [file DataSheet_1.zip › 1.durgSenstivity/durgSenstivity.Roscovitine.pdf]

Risk 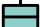 low 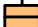 high

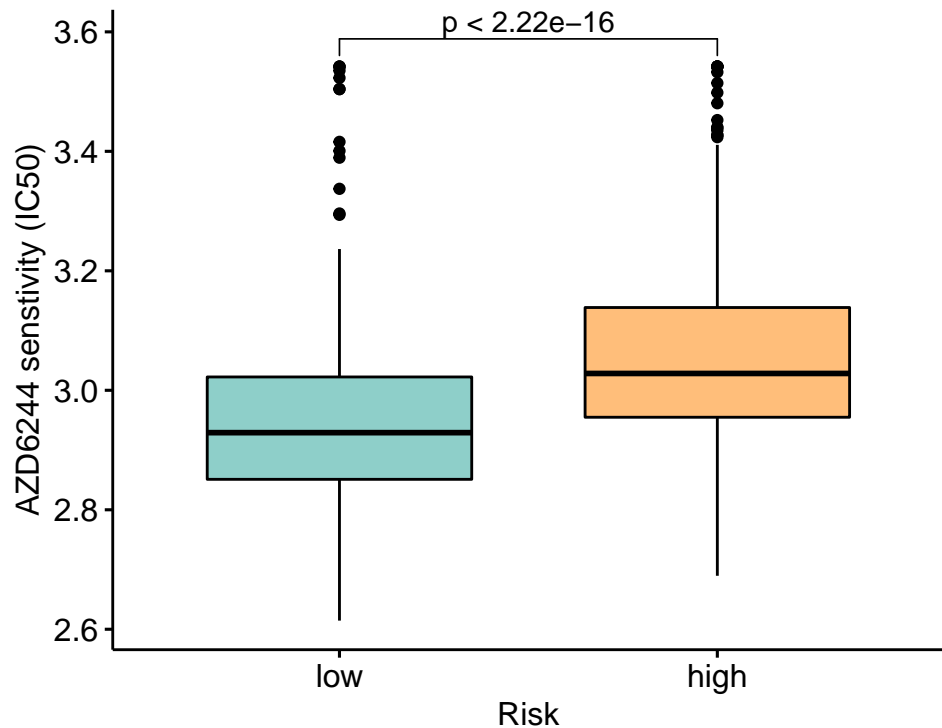

Supplement: Supplementary File 1 — 98 drugs were with significant differences in IC50 concentrations between high and low risk groups. [file DataSheet_1.zip › 1.durgSenstivity/durgSenstivity.AZD6244.pdf]

Risk 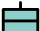 low 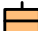 high

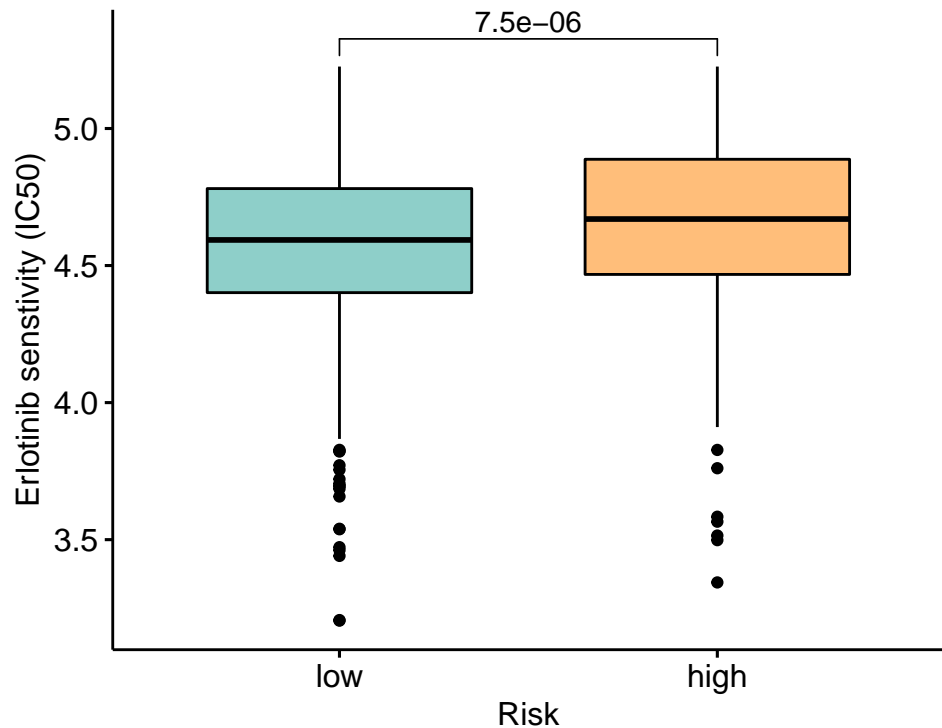

Supplement: Supplementary File 1 — 98 drugs were with significant differences in IC50 concentrations between high and low risk groups. [file DataSheet_1.zip › 1.durgSenstivity/durgSenstivity.Erlotinib.pdf]

Risk 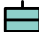 low 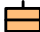 high

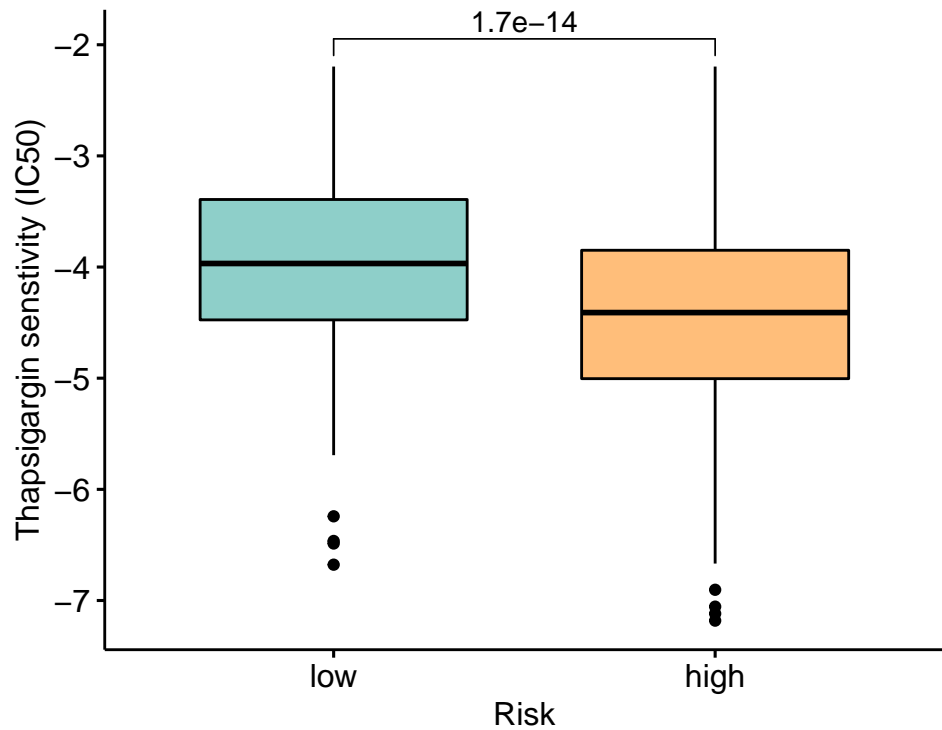

Supplement: Supplementary File 1 — 98 drugs were with significant differences in IC50 concentrations between high and low risk groups. [file DataSheet_1.zip › 1.durgSenstivity/durgSenstivity.Thapsigargin.pdf]

Risk 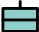 low 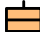 high

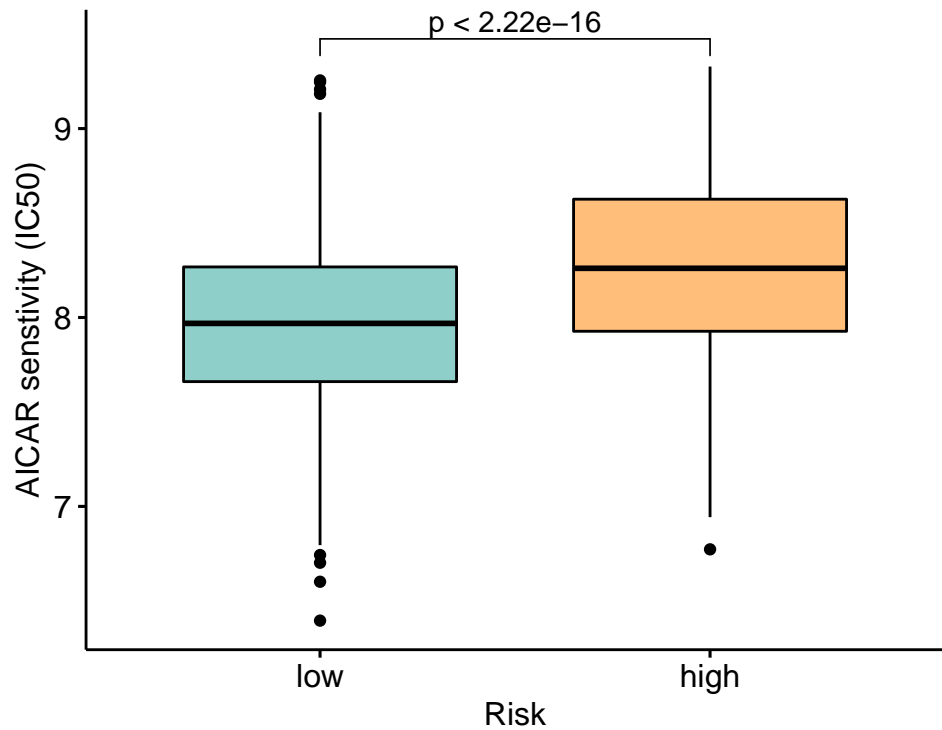

Supplement: Supplementary File 1 — 98 drugs were with significant differences in IC50 concentrations between high and low risk groups. [file DataSheet_1.zip › 1.durgSenstivity/durgSenstivity.AICAR.pdf]

Risk 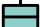 low 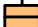 high

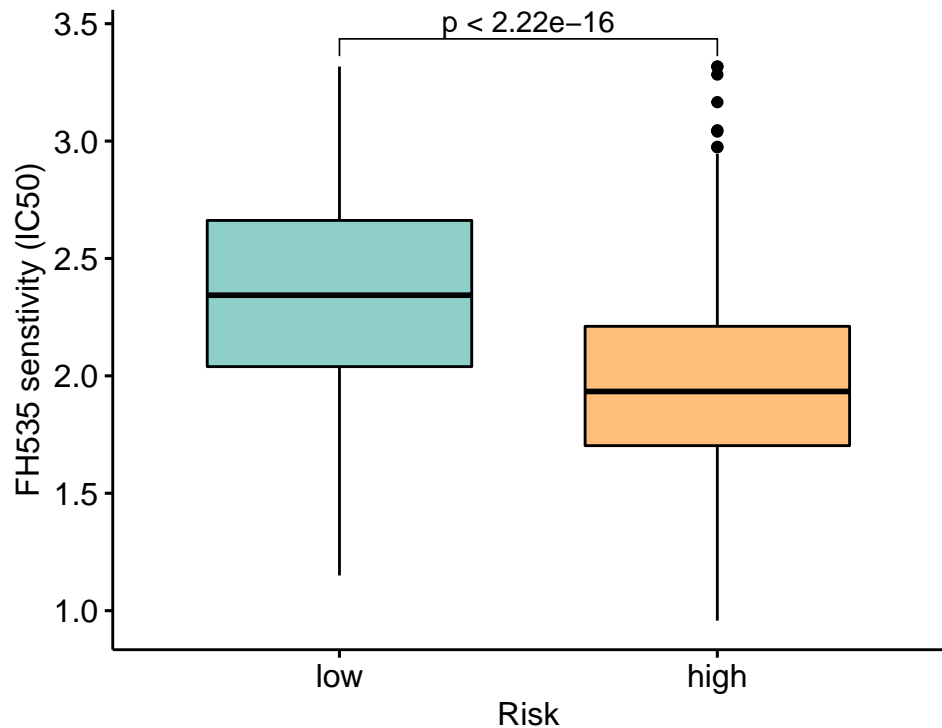

Supplement: Supplementary File 1 — 98 drugs were with significant differences in IC50 concentrations between high and low risk groups. [file DataSheet_1.zip › 1.durgSenstivity/durgSenstivity.FH535.pdf]

Risk 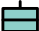 low 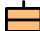 high

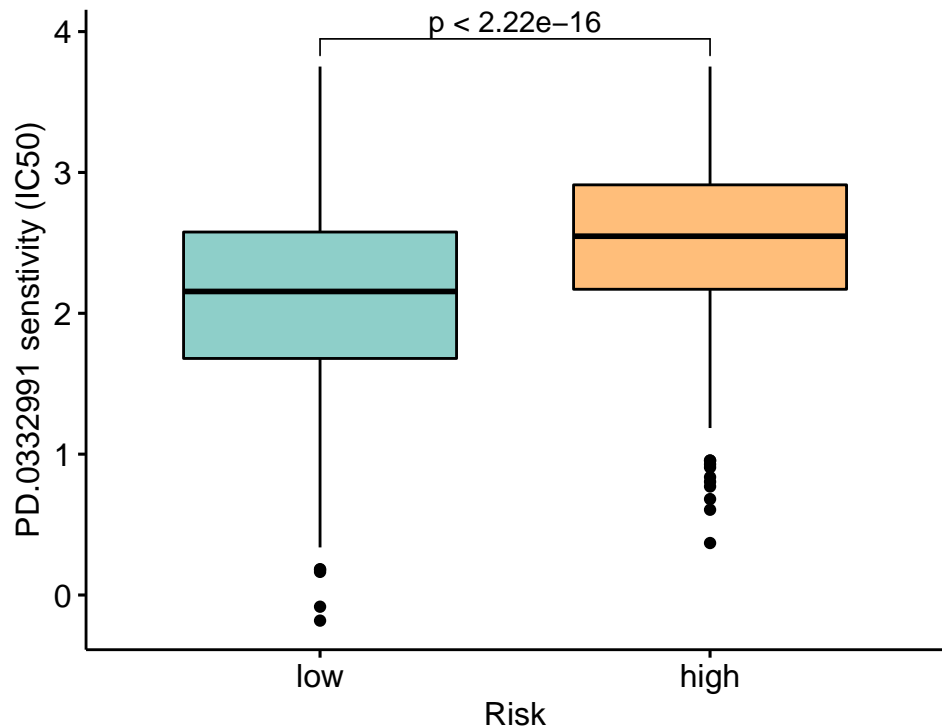

Supplement: Supplementary File 1 — 98 drugs were with significant differences in IC50 concentrations between high and low risk groups. [file DataSheet_1.zip › 1.durgSenstivity/durgSenstivity.PD.0332991.pdf]

Risk 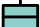 low 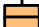 high

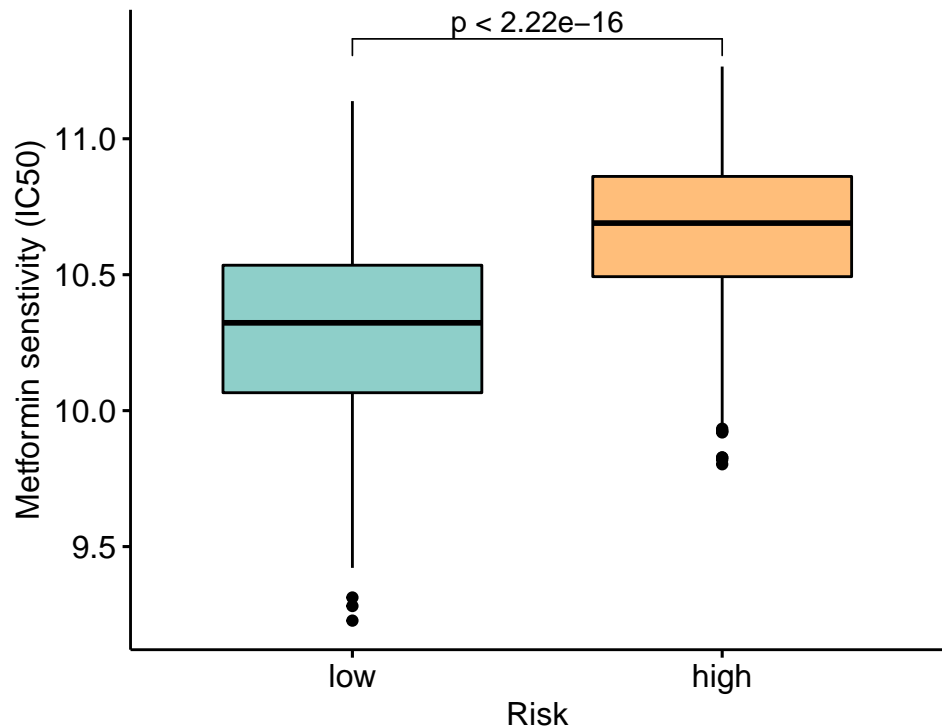

Supplement: Supplementary File 1 — 98 drugs were with significant differences in IC50 concentrations between high and low risk groups. [file DataSheet_1.zip › 1.durgSenstivity/durgSenstivity.Metformin.pdf]

Risk 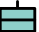 low 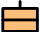 high

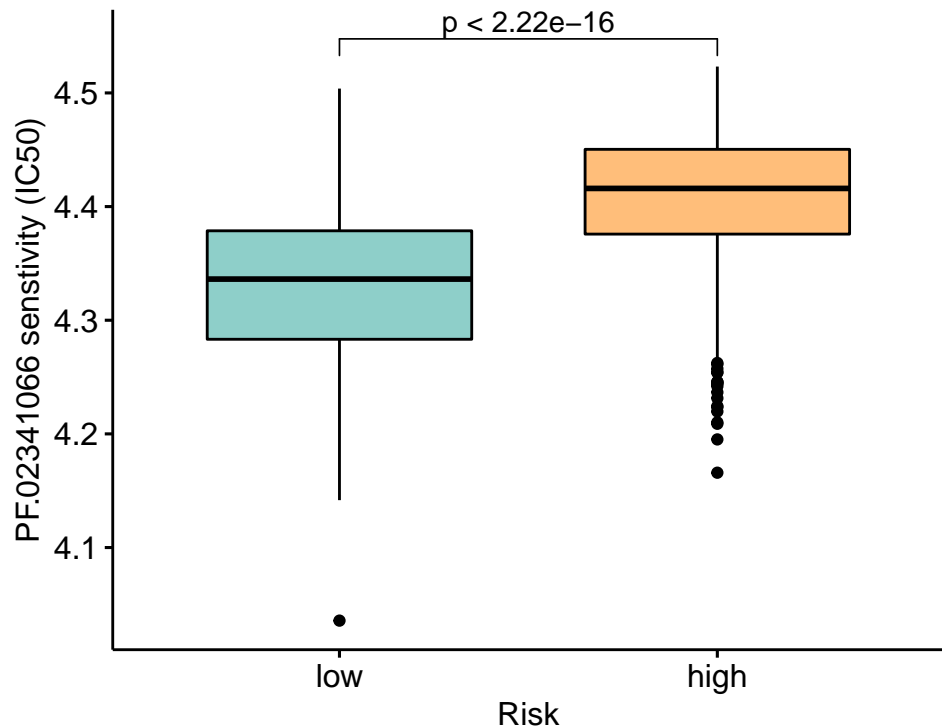

Supplement: Supplementary File 1 — 98 drugs were with significant differences in IC50 concentrations between high and low risk groups. [file DataSheet_1.zip › 1.durgSenstivity/durgSenstivity.PF.02341066.pdf]

Risk 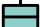 low 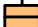 high

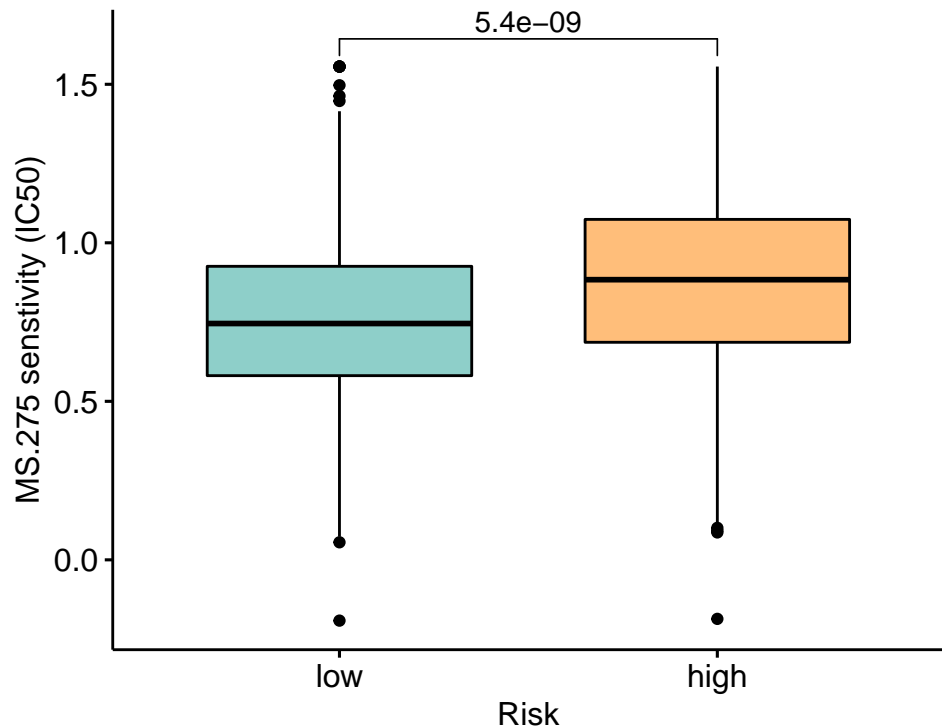

Supplement: Supplementary File 1 — 98 drugs were with significant differences in IC50 concentrations between high and low risk groups. [file DataSheet_1.zip › 1.durgSenstivity/durgSenstivity.MS.275.pdf]

Risk 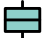 low 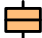 high

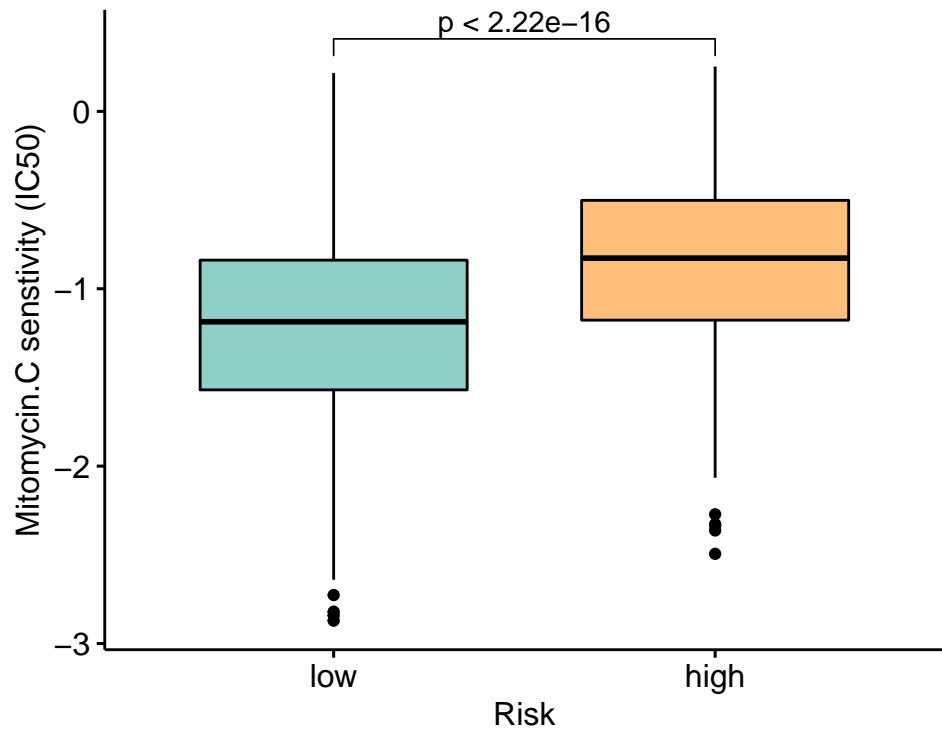

Supplement: Supplementary File 1 — 98 drugs were with significant differences in IC50 concentrations between high and low risk groups. [file DataSheet_1.zip › 1.durgSenstivity/durgSenstivity.Mitomycin.C.pdf]

Risk 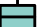 low 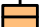 high

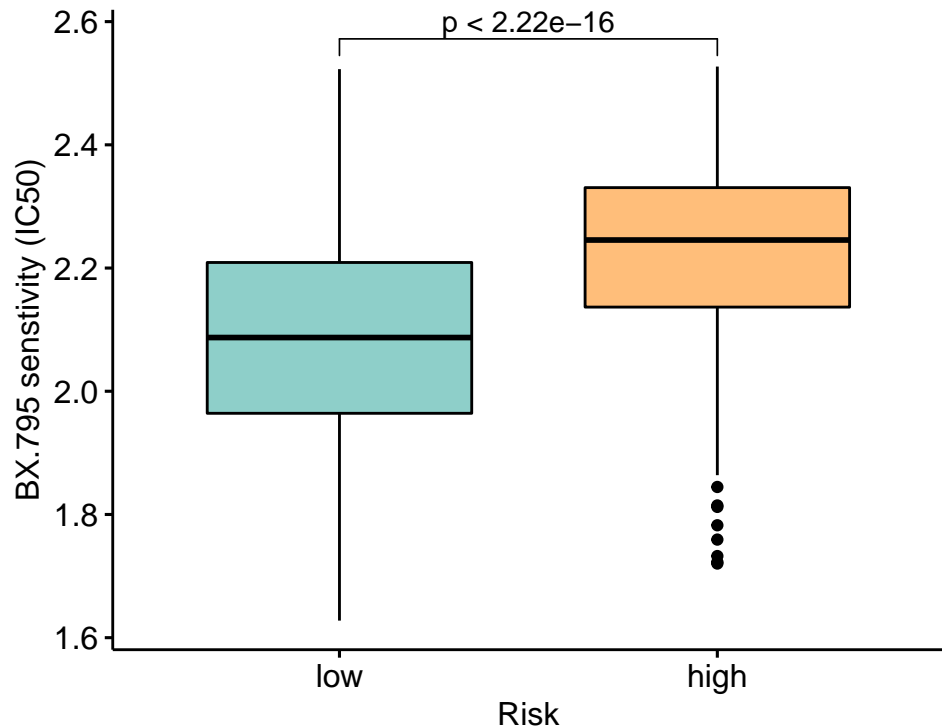

Supplement: Supplementary File 1 — 98 drugs were with significant differences in IC50 concentrations between high and low risk groups. [file DataSheet_1.zip › 1.durgSenstivity/durgSenstivity.BX.795.pdf]

Risk 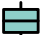 low 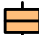 high

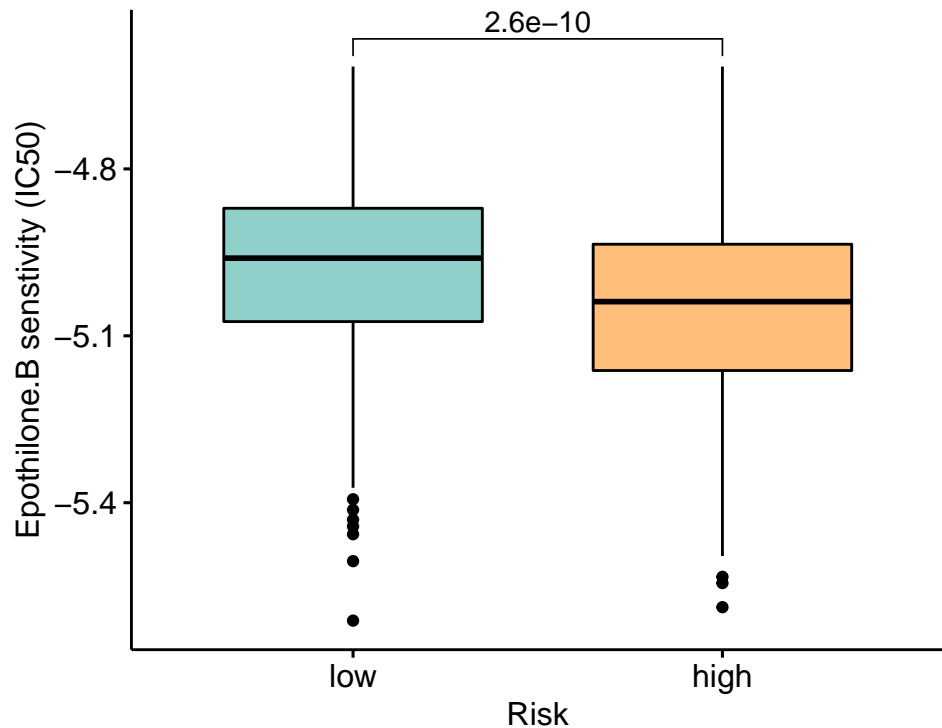

Supplement: Supplementary File 1 — 98 drugs were with significant differences in IC50 concentrations between high and low risk groups. [file DataSheet_1.zip › 1.durgSenstivity/durgSenstivity.Epothilone.B.pdf]

Risk 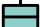 low 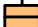 high

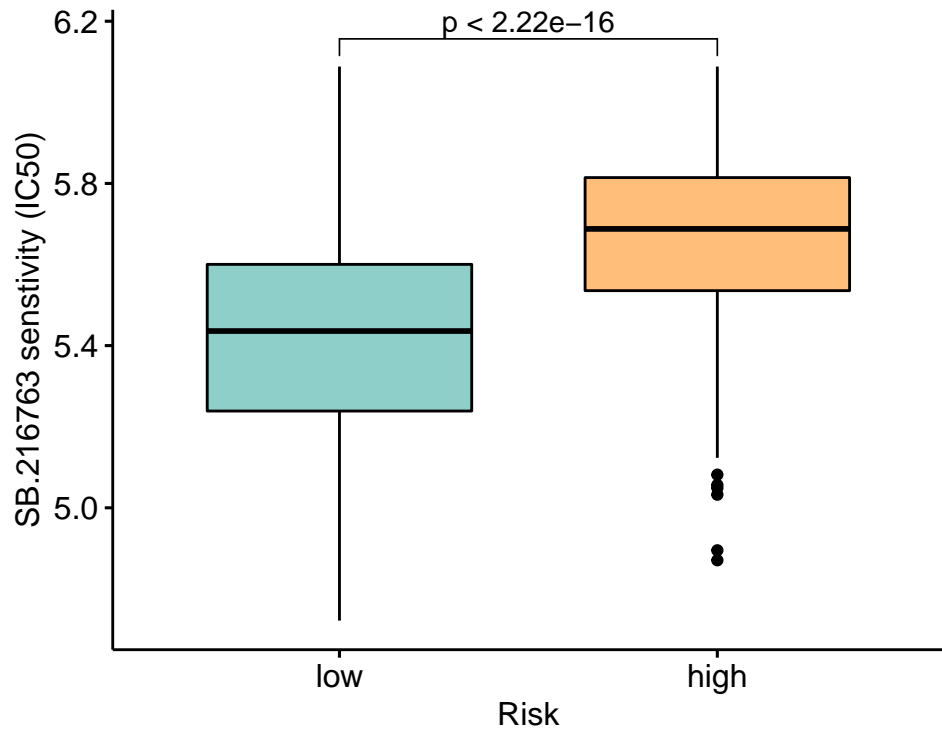

Supplement: Supplementary File 1 — 98 drugs were with significant differences in IC50 concentrations between high and low risk groups. [file DataSheet_1.zip › 1.durgSenstivity/durgSenstivity.SB.216763.pdf]

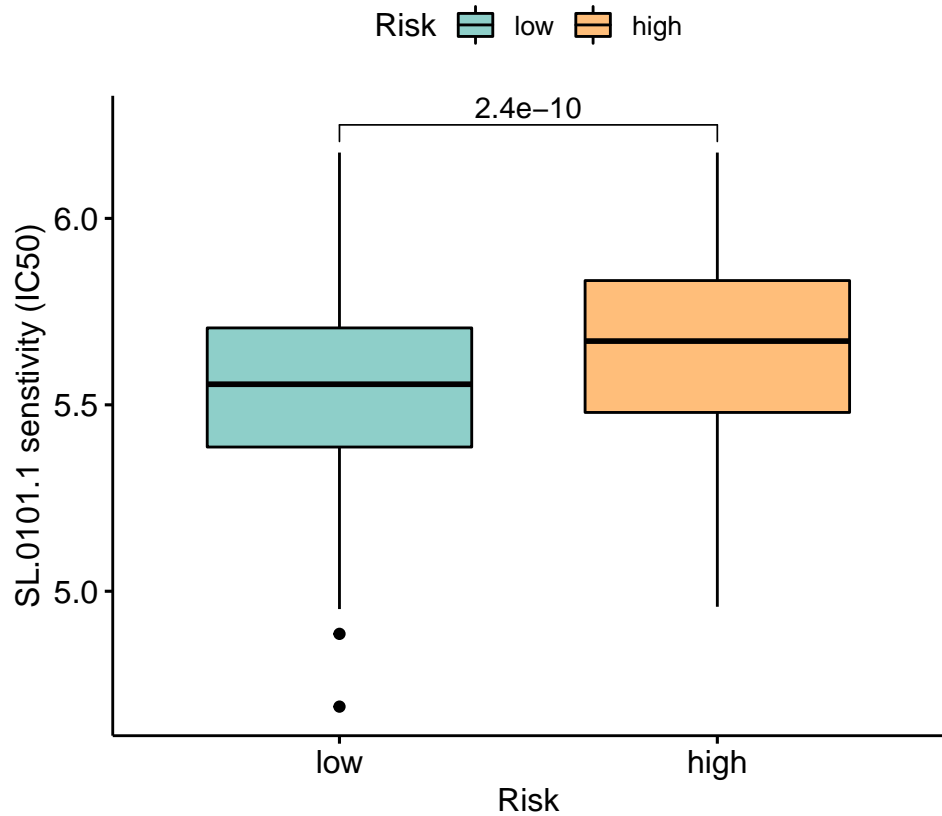

Supplement: Supplementary File 1 — 98 drugs were with significant differences in IC50 concentrations between high and low risk groups. [file DataSheet_1.zip › 1.durgSenstivity/durgSenstivity.SL.0101.1.pdf]

Risk 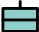 low 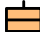 high

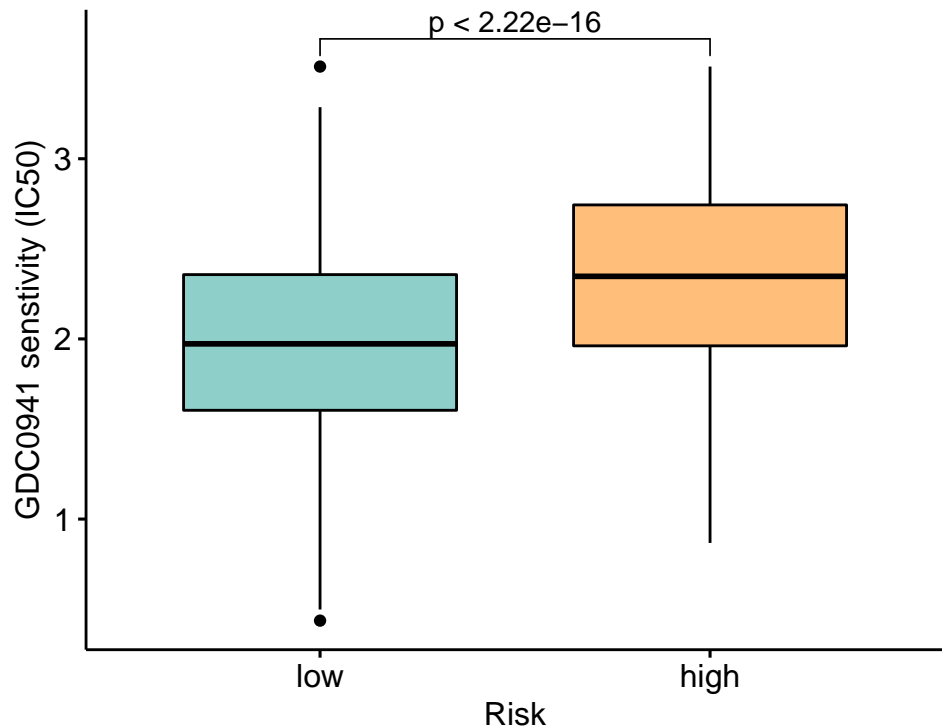

Supplement: Supplementary File 1 — 98 drugs were with significant differences in IC50 concentrations between high and low risk groups. [file DataSheet_1.zip › 1.durgSenstivity/durgSenstivity.GDC0941.pdf]

Risk 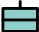 low 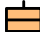 high

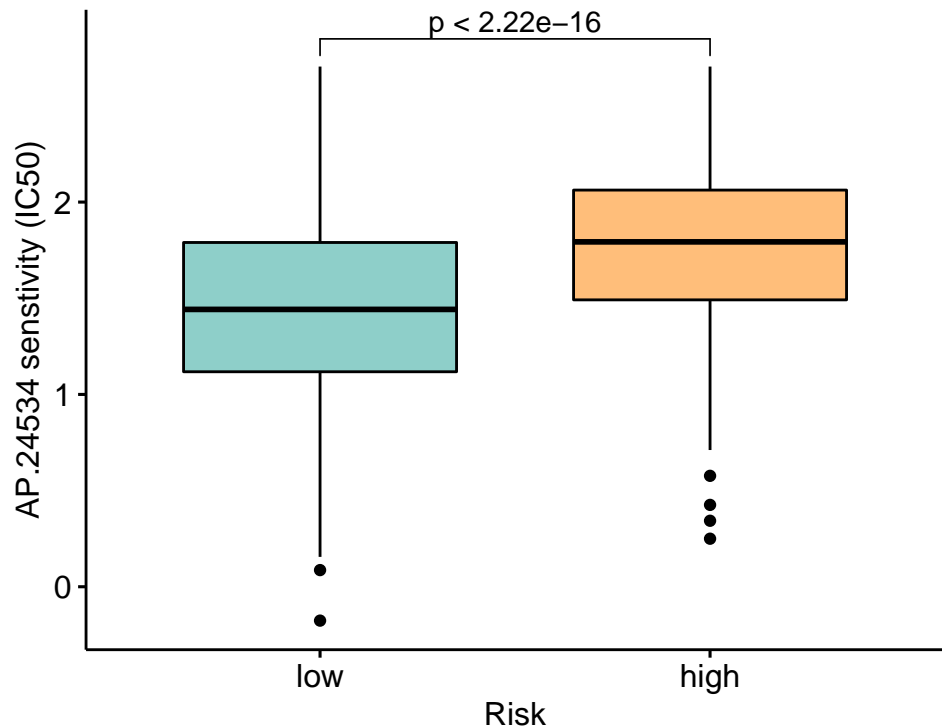

Supplement: Supplementary File 1 — 98 drugs were with significant differences in IC50 concentrations between high and low risk groups. [file DataSheet_1.zip › 1.durgSenstivity/durgSenstivity.AP.24534.pdf]

Risk 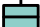 low 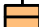 high

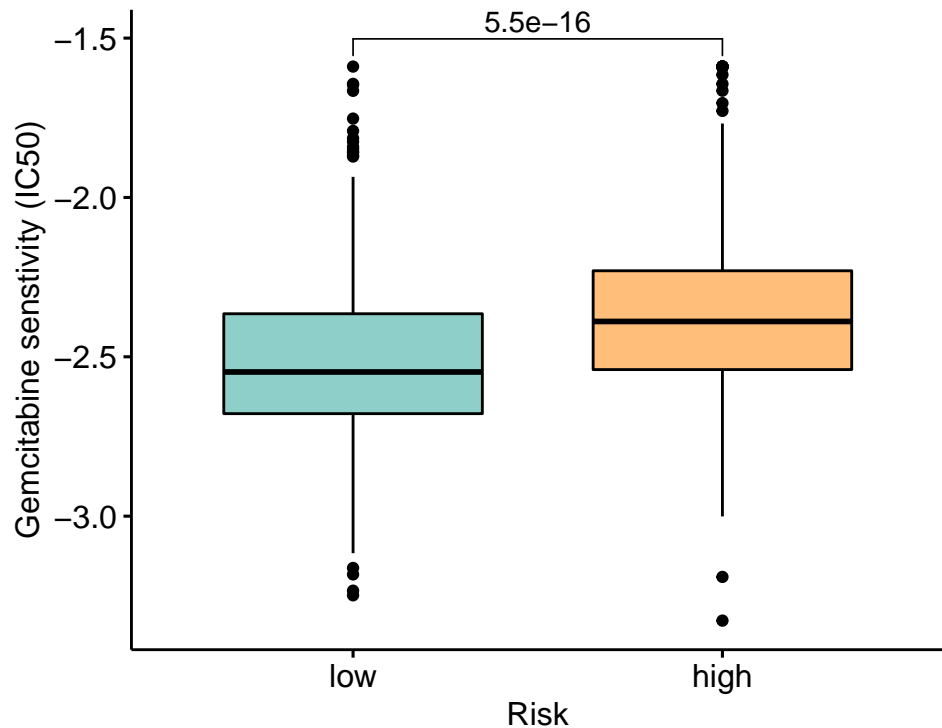

Supplement: Supplementary File 1 — 98 drugs were with significant differences in IC50 concentrations between high and low risk groups. [file DataSheet_1.zip › 1.durgSenstivity/durgSenstivity.Gemcitabine.pdf]

Risk 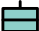 low 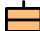 high

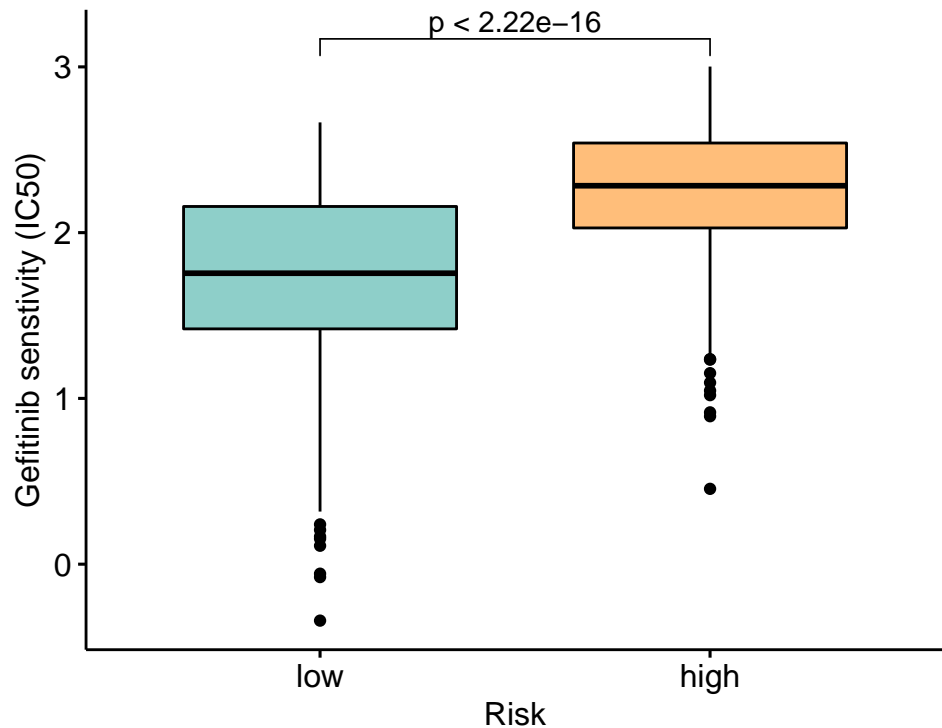

Supplement: Supplementary File 1 — 98 drugs were with significant differences in IC50 concentrations between high and low risk groups. [file DataSheet_1.zip › 1.durgSenstivity/durgSenstivity.Gefitinib.pdf]

Risk 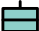 low 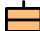 high

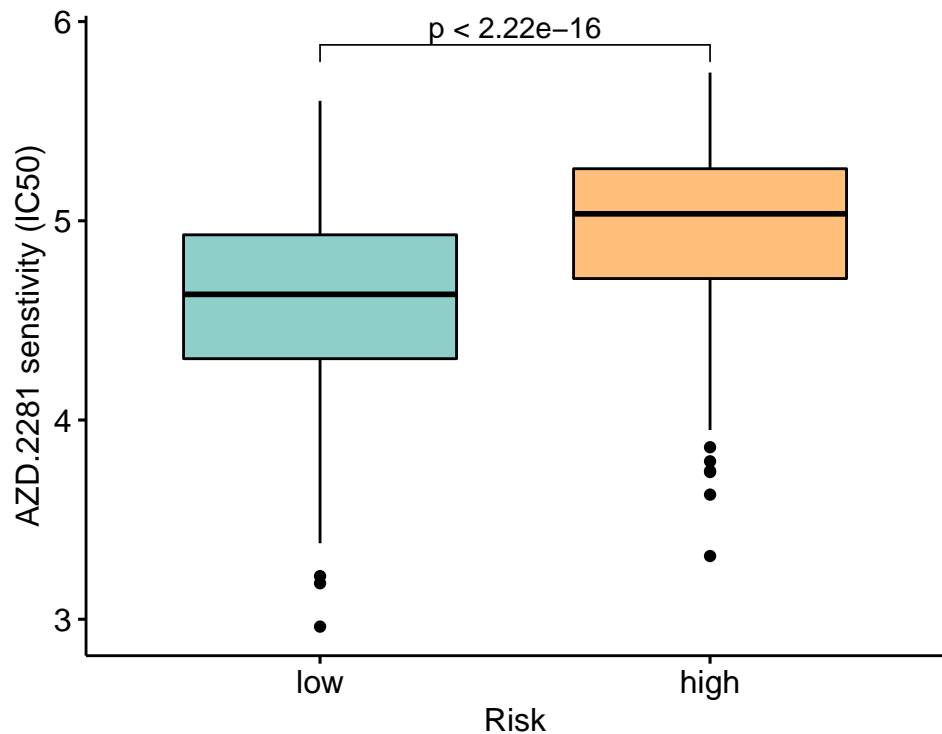

Supplement: Supplementary File 1 — 98 drugs were with significant differences in IC50 concentrations between high and low risk groups. [file DataSheet_1.zip › 1.durgSenstivity/durgSenstivity.AZD.2281.pdf]

Risk 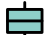 low 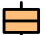 high

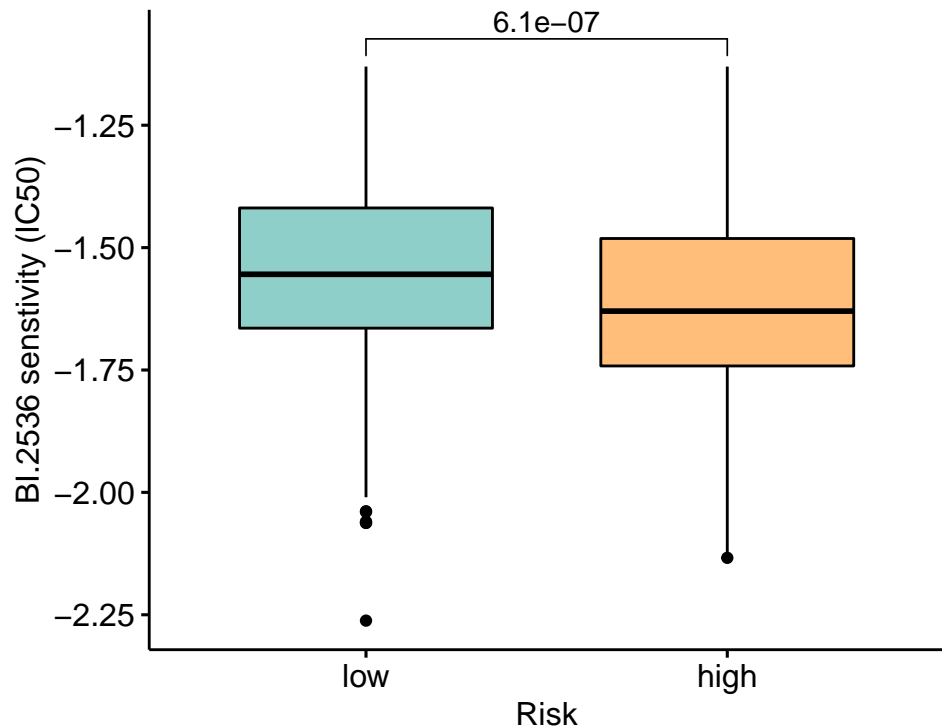

Supplement: Supplementary File 1 — 98 drugs were with significant differences in IC50 concentrations between high and low risk groups. [file DataSheet_1.zip › 1.durgSenstivity/durgSenstivity.BI.2536.pdf]

Risk 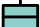 low 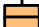 high

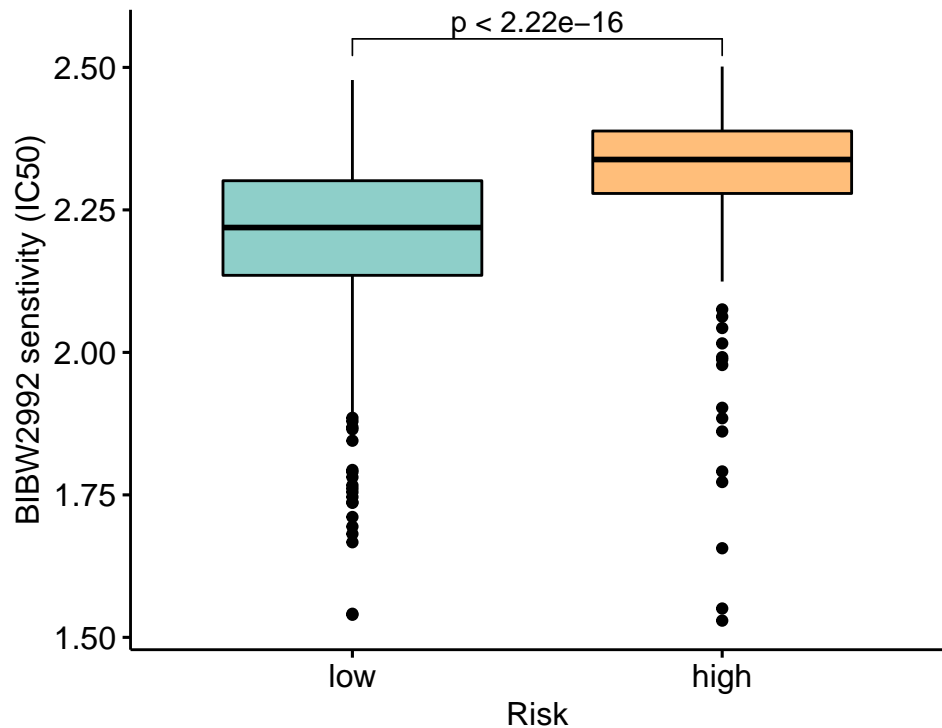

Supplement: Supplementary File 1 — 98 drugs were with significant differences in IC50 concentrations between high and low risk groups. [file DataSheet_1.zip › 1.durgSenstivity/durgSenstivity.BIBW2992.pdf]

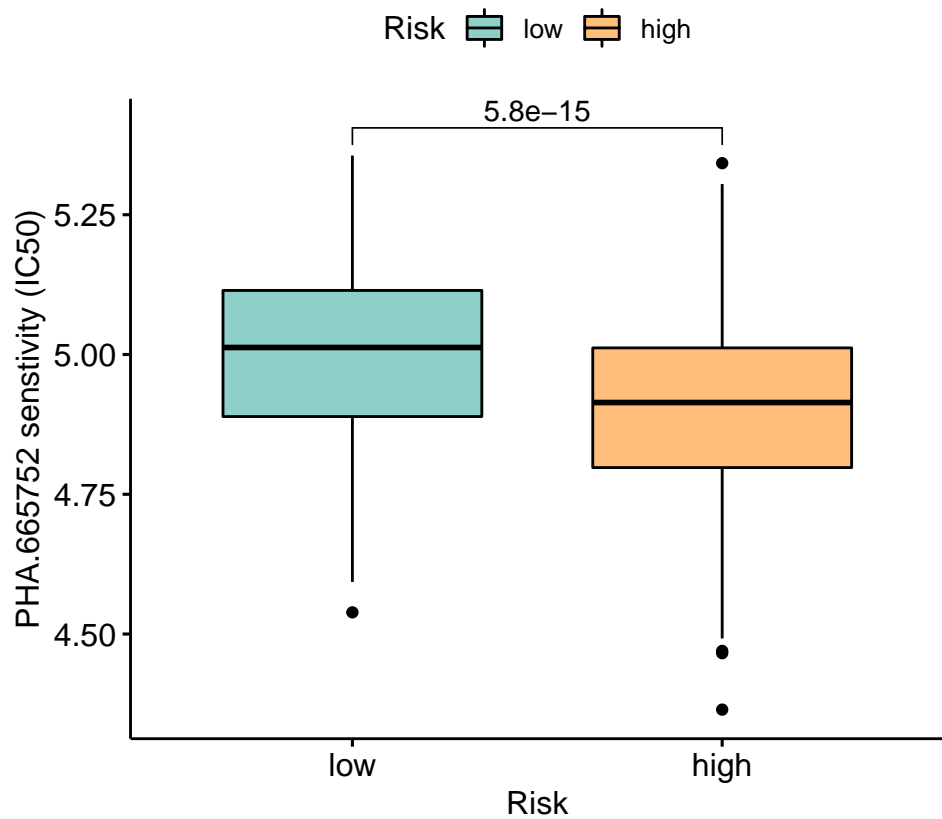

Supplement: Supplementary File 1 — 98 drugs were with significant differences in IC50 concentrations between high and low risk groups. [file DataSheet_1.zip › 1.durgSenstivity/durgSenstivity.PHA.665752.pdf]

Risk 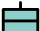 low 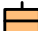 high

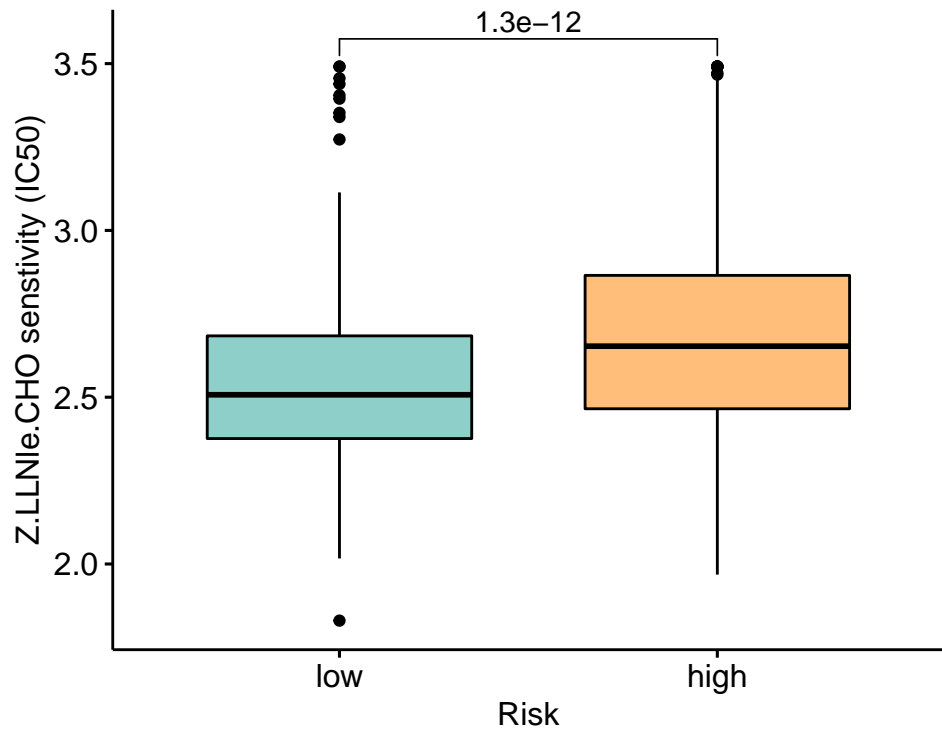

Supplement: Supplementary File 1 — 98 drugs were with significant differences in IC50 concentrations between high and low risk groups. [file DataSheet_1.zip › 1.durgSenstivity/durgSenstivity.Z.LLNle.CHO.pdf]

Risk 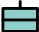 low 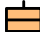 high

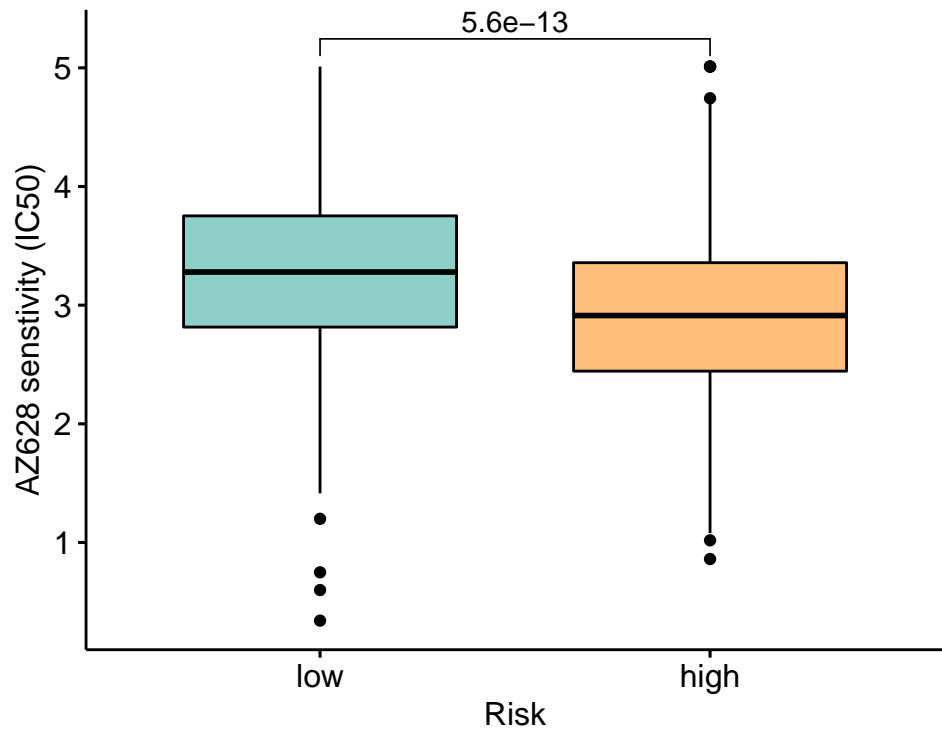

Supplement: Supplementary File 1 — 98 drugs were with significant differences in IC50 concentrations between high and low risk groups. [file DataSheet_1.zip › 1.durgSenstivity/durgSenstivity.AZ628.pdf]

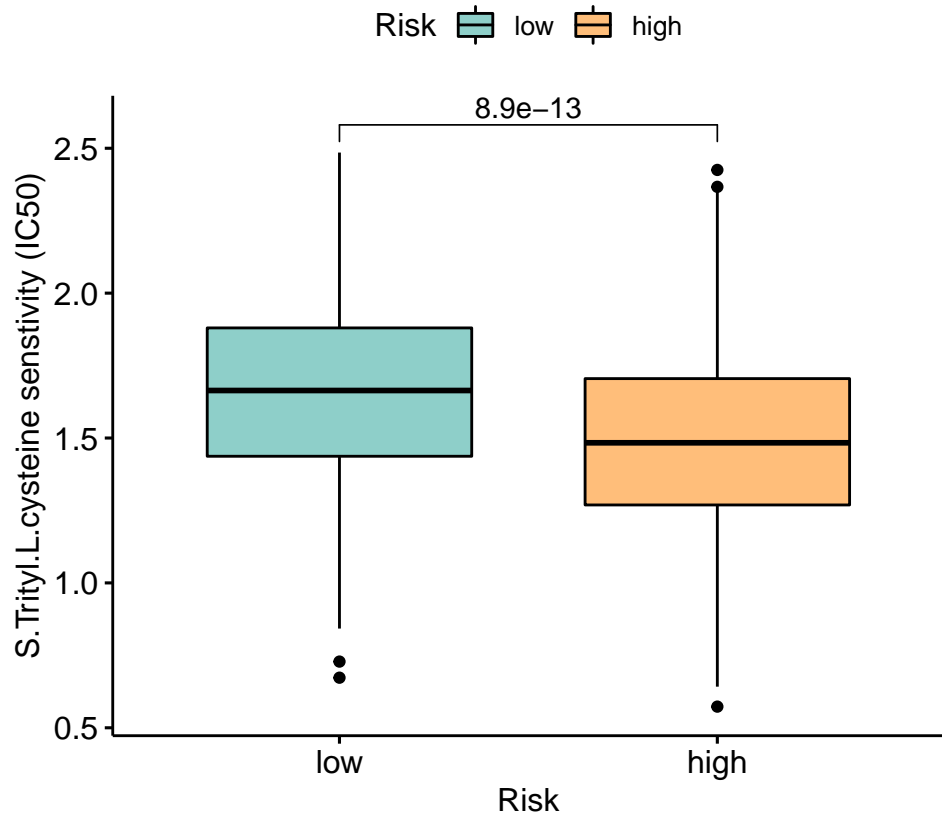

Supplement: Supplementary File 1 — 98 drugs were with significant differences in IC50 concentrations between high and low risk groups. [file DataSheet_1.zip › 1.durgSenstivity/durgSenstivity.S.Trityl.L.cysteine.pdf]

Risk 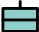 low 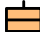 high

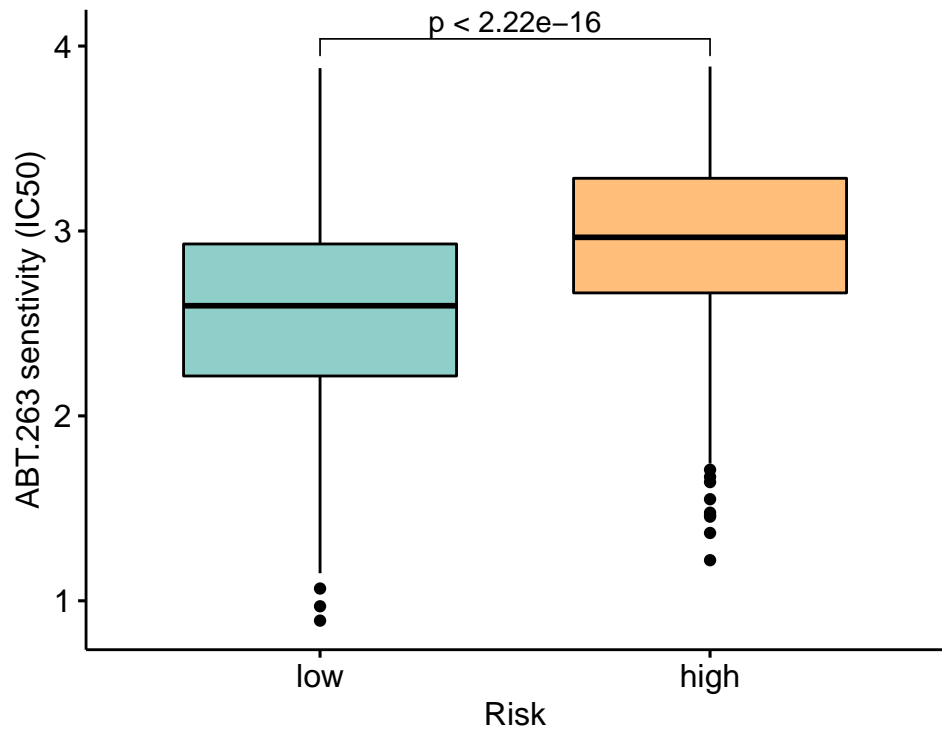

Supplement: Supplementary File 1 — 98 drugs were with significant differences in IC50 concentrations between high and low risk groups. [file DataSheet_1.zip › 1.durgSenstivity/durgSenstivity.ABT.263.pdf]

Risk 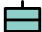 low 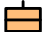 high

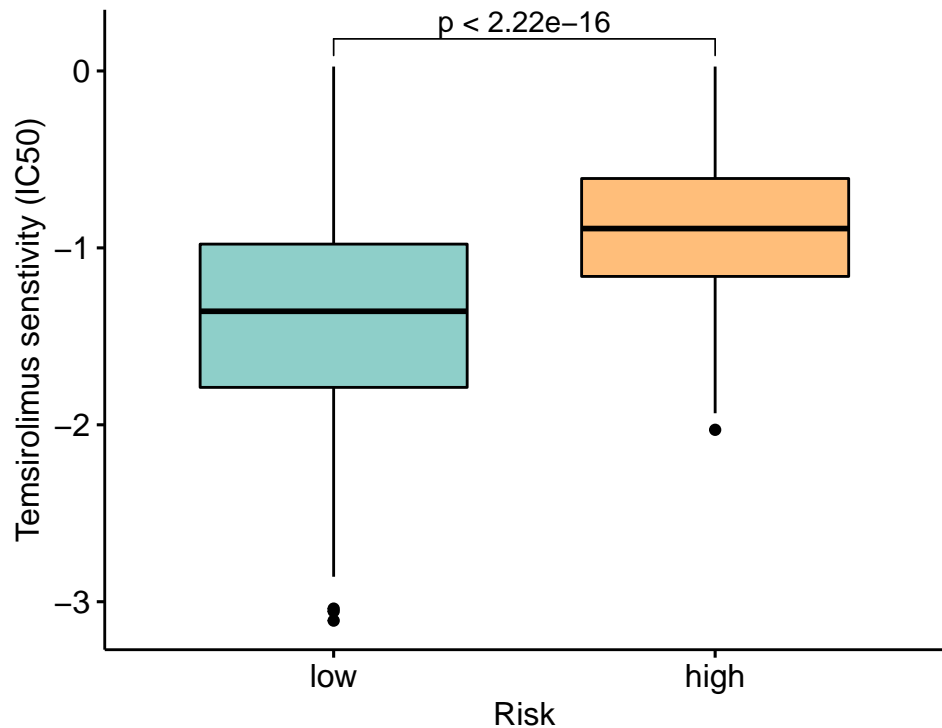

Supplement: Supplementary File 1 — 98 drugs were with significant differences in IC50 concentrations between high and low risk groups. [file DataSheet_1.zip › 1.durgSenstivity/durgSenstivity.Temsirolimus.pdf]

Risk 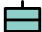 low 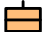 high

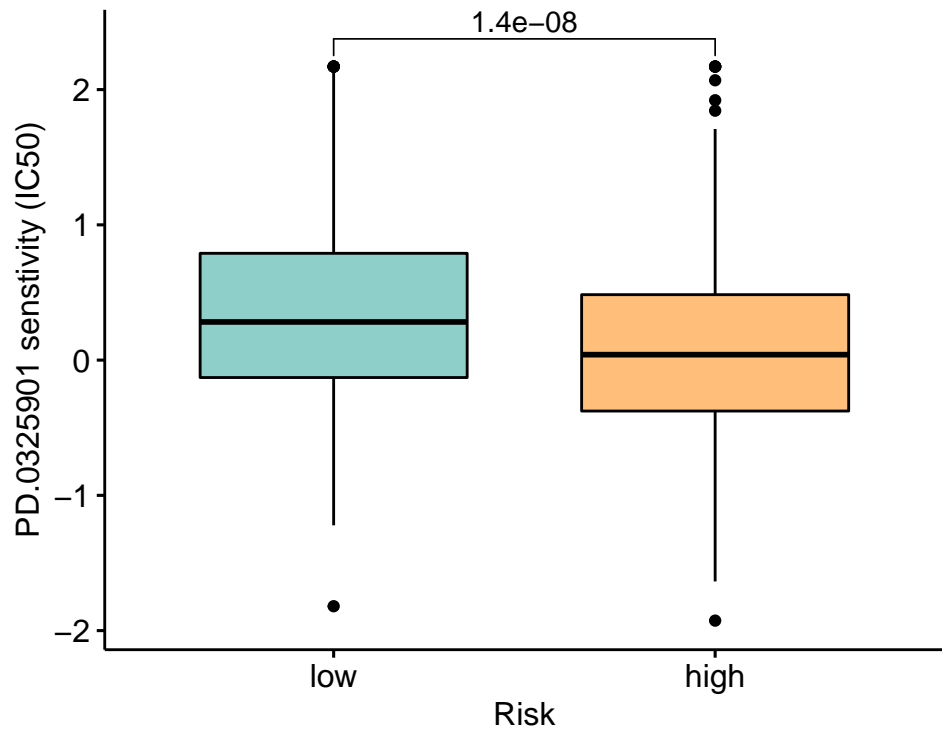

Supplement: Supplementary File 1 — 98 drugs were with significant differences in IC50 concentrations between high and low risk groups. [file DataSheet_1.zip › 1.durgSenstivity/durgSenstivity.PD.0325901.pdf]

Risk 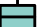 low 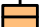 high

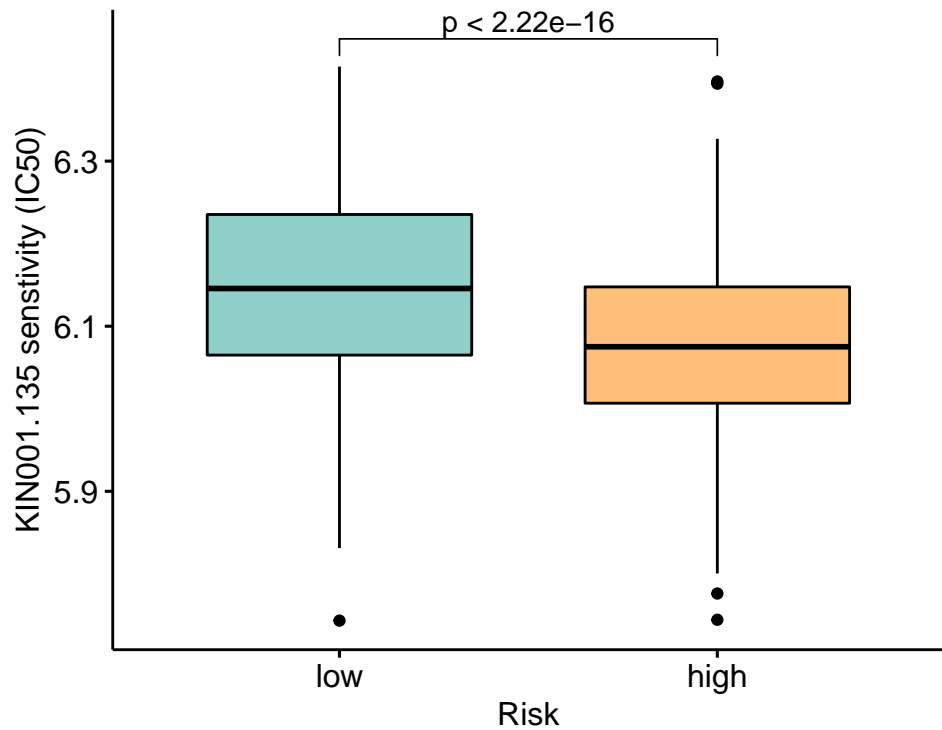

Supplement: Supplementary File 1 — 98 drugs were with significant differences in IC50 concentrations between high and low risk groups. [file DataSheet_1.zip › 1.durgSenstivity/durgSenstivity.KIN001.135.pdf]

AUY922 sensitivity (IC50)

Risk 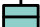 low 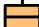 high

$1.9\text{e-}11$

low

high

Risk

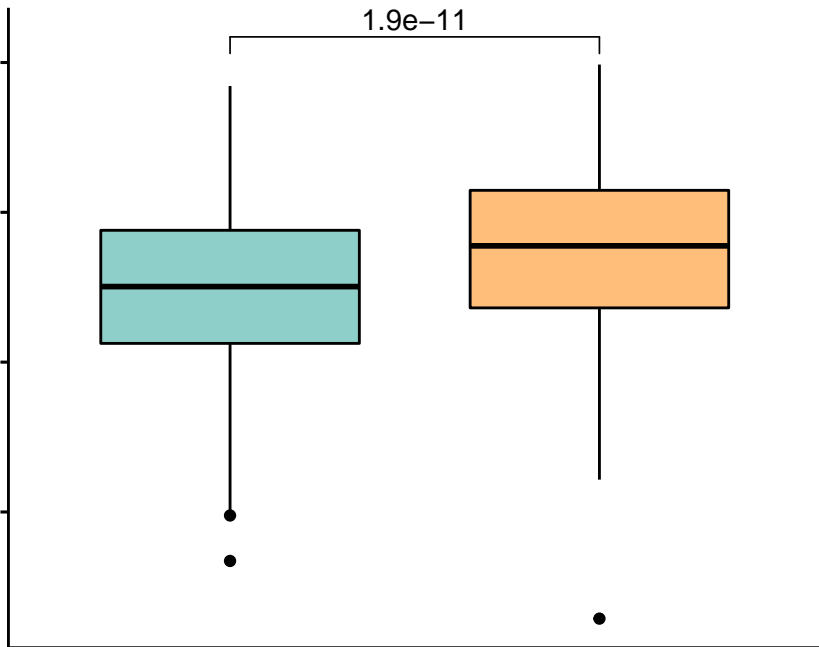

Supplement: Supplementary File 1 — 98 drugs were with significant differences in IC50 concentrations between high and low risk groups. [file DataSheet_1.zip › 1.durgSenstivity/durgSenstivity.AUY922.pdf]

BMS.708163 sensitivity (IC50)

Risk 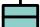 low 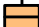 high

$1.8e-13$

low

high

Risk

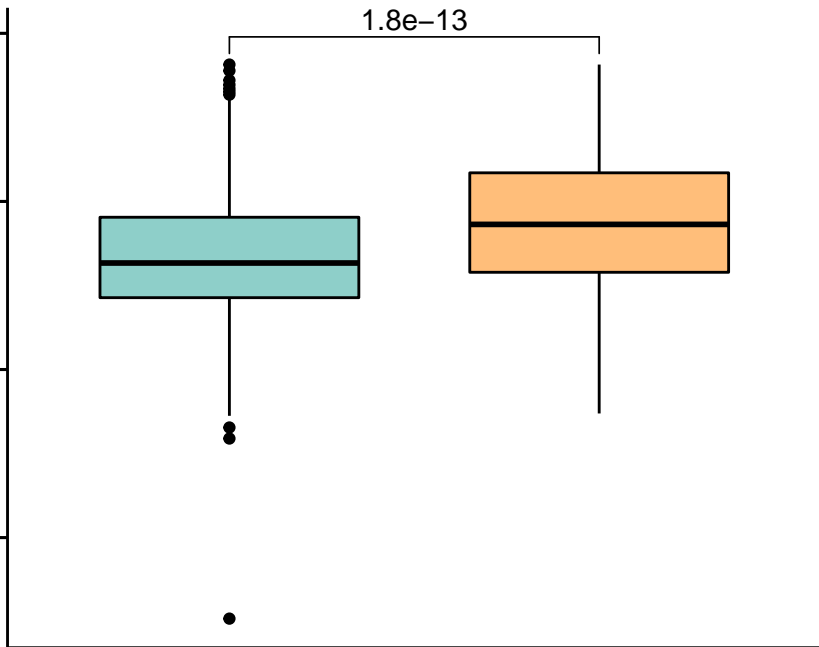

Supplement: Supplementary File 1 — 98 drugs were with significant differences in IC50 concentrations between high and low risk groups. [file DataSheet_1.zip › 1.durgSenstivity/durgSenstivity.BMS.708163.pdf]

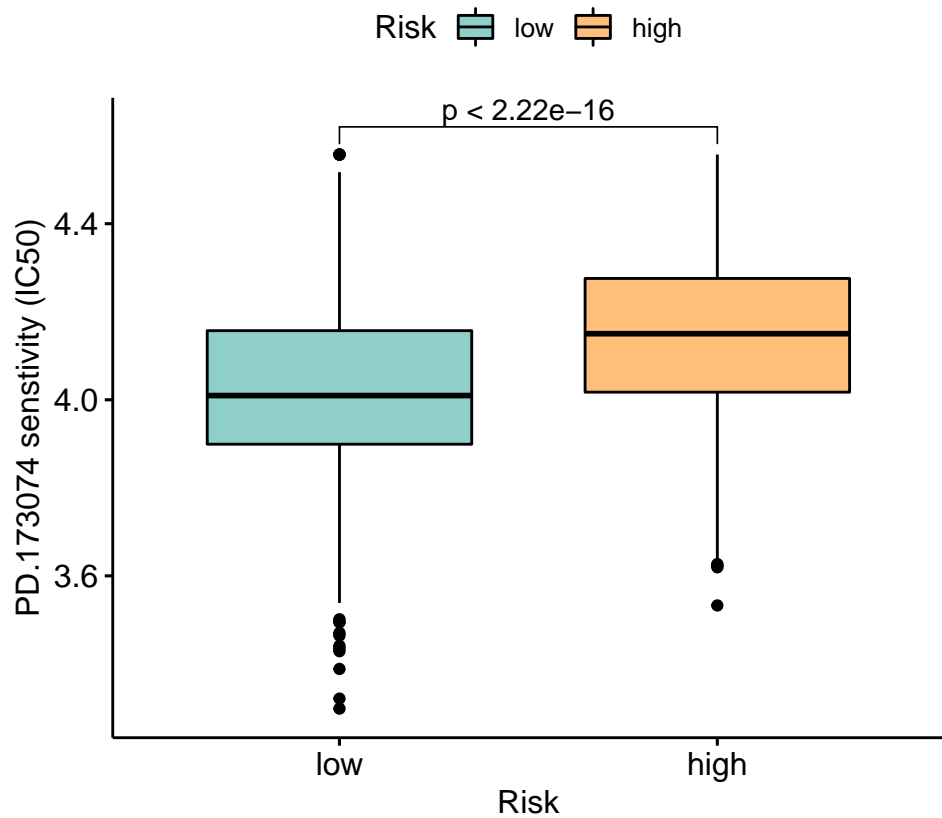

Supplement: Supplementary File 1 — 98 drugs were with significant differences in IC50 concentrations between high and low risk groups. [file DataSheet_1.zip › 1.durgSenstivity/durgSenstivity.PD.173074.pdf]
