# Supplementary material for: The role of cuproptosis-related gene in the classification and prognosis of melanoma
Source: Front Immunol. 2022 Oct 19;13:986214. doi: 10.3389/fimmu.2022.986214 (PMC9632664; doi:10.3389/fimmu.2022.986214)
Supplement: Supplementary Table 1 — Names of 13 cuproptosis-related genes [file Table_1.pdf]

id
FDX1
LIPT1
LIAS
DLD
DBT
GCSH
DLST
DLAT
PDHA1
PDHB
SLC31A1
ATP7A
ATP7B
