# Supplementary material for: The role of cuproptosis-related gene in the classification and prognosis of melanoma
Source: Front Immunol. 2022 Oct 19;13:986214. doi: 10.3389/fimmu.2022.986214 (PMC9632664; doi:10.3389/fimmu.2022.986214)
Supplement: Supplementary File 1 — 98 drugs were with significant differences in IC50 concentrations between high and low risk groups. [file durgSenstivity.Camptothecin.pdf › 1.durgSenstivity/durgSenstivity.Camptothecin.pdf]

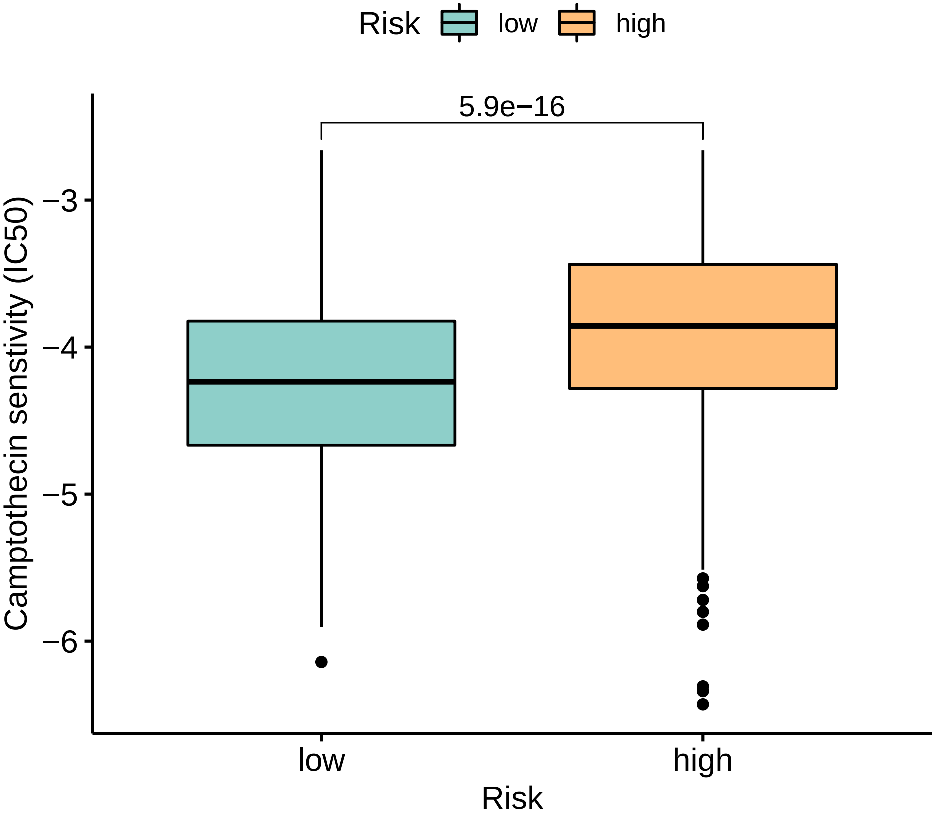

Risk
low
high
5.9e−16
−3
−4
−5
−6
Camptothecin senstivity (IC50)
low
high
Risk
